# Sedentary behaviour in non-ambulant children and young people with physical disabilities: a systematic search and review protocol

**DOI:** 10.1136/bmjopen-2021-053077

**Published:** 2021-12-03

**Authors:** Marilyn Bradbury, Ciara O'Brien, Nathan Giles, Sally Fenton, Sue Neilson, Joan L Duda

**Affiliations:** 1 Research and Innovation, Birmingham Community Healthcare NHS Foundation Trust, Aston, UK; 2 School of Sport, Exercise and Rehabilitation Sciences, University of Birmingham, Birmingham, UK; 3 School of Psychology, University of Surrey, Guildford, Surrey, UK; 4 Public and Patient Involvement Representative (Volunteer), Birmingham Community Healthcare NHS Foundation Trust, Aston, Birmingham, UK; 5 School of Nursing, University of Birmingham, Birmingham, UK

**Keywords:** developmental neurology & neurodisability, rehabilitation medicine, social medicine

## Abstract

**Introduction:**

Non-ambulant children and young people with physical disabilities are at high risk of experiencing negative health outcomes associated with sedentary time. A previous scoping review summarising evidence relating to sedentary behaviours of children with physical disabilities identified the need for validated methods of measuring physical activity of children who use wheelchairs and evaluation of interventions to reduce sedentary time. The scoping review did not assess the quality of evidence relating to this topic, therefore its validity remains unclear. No reviews focussing on non-ambulant children and young people up to the age of 25 years have been undertaken.

The objectives of this systematic search and review are to:

1. Identify all peer-reviewed articles relating to sedentary behaviour of non-ambulant children and young people.
2. Categorise the articles according to study design and four subquestions relating to (i) measurement, (ii) patterns, (iii) associated risks and (iv) interventions to reduce sedentary time or behaviour.
3. Critically appraise quality of the articles using established critical appraisal tools.
4. Summarise the evidence for each subquestion. Describe its cumulative strength and identify knowledge gaps.

**Methods and analysis:**

This protocol was developed using the Preferred Reporting Items for Systematic Review and Meta-Analysis Protocols (PRISMA-P) checklist. The research questions, inclusion/exclusion criteria and search terms have been developed a priori using the ‘Population, Concept and Context’ framework. Online databases will be systematically searched to identify peer-reviewed articles published between 1996 and 2021. Two reviewers will screen, categorise and critically appraise the articles. Data extraction and analysis will be verified by the second reviewer.

Results will be reported as a best evidence synthesis, with reference to the PRISMA checklist.

**Ethics and dissemination:**

Ethical approval is not required. The review will be submitted to an appropriate peer-reviewed journal.

**Registration:**

The review is registered on the Open Science Framework database. DOI: https://doi.org/10.17605/OSF.IO/SQXJB. Any protocol amendments will be recorded in the Open Science Framework database.

**Strengths and limitations of this study**

- This review systematically identifies evidence relating to sedentary behaviours of non-ambulant children and young people using a robust and comprehensive search strategy.
- The review builds on previously published literature through the addition of an assessment of the quality of the included research.
- A wide age range is incorporated, allowing consideration of how sedentary behaviour changes on the journey from childhood into adulthood.
- A limitation is that only peer-reviewed literature will be included.
- A limitation is that only studies which are written in English will be included.

## Introduction

Sedentary behaviour is defined as ‘any waking behaviour characterised by an energy expenditure ≤1.5 metabolic equivalents (METs) while in a sitting or reclining posture’.^1^ A large body of evidence from a variety of study designs suggests that reducing sedentary time is associated with lower health risk in children and young people aged 5–17 years.^2^ This body of evidence includes studies measuring both sedentary time and specific behaviours such as television watching. In children, time spent watching television has been associated with increased body mass index, reduced physical fitness, low self-esteem and reduced academic achievement.^2^

Children with disabilities are more sedentary than their able bodied peers.^3^ Lower levels of gross motor function^4–6^ and increasing age^6–8^ are linked to increased sedentary behaviours in children with disabilities. Young people who are non-ambulant (ie, unable to walk or can walk short distances using a body support walker (‘therapeutic walking’)), are therefore at greatest risk of experiencing negative health outcomes associated with sedentary time. Young people in this group may have conditions such as (but not limited to) cerebral palsy, spina bifida, muscular dystrophy, irreversible traumatic injuries or rare conditions.

A scoping review of sedentary behaviour and children with physical disabilities has recently summarised research relating to measurement of sedentary time, sedentary behaviour patterns and interventions to reduce or break up time spent sedentary.^3^ The review identified that there is a lack of research evaluating interventions to decrease the sedentary behaviours of children with physical disabilities. In addition, the need for research that focuses on improving our understanding of sedentary behaviour patterns in children who are non-ambulant, including the validity of using accelerometers to measure sedentary time in this group, was recognised. To date, no reviews incorporating a quality assessment of research relating to this topic or focussing specifically on non-ambulant children and young people have been undertaken. This review builds on that of Ganz *et al*,^3^ by focusing specifically on non-ambulant children and young people, incorporating critical evaluation of the quality of the evidence, considering risks associated with sedentary time or behaviour for this population and searching a wider range of databases. Contemporary work that has been published since the searches for the scoping review were undertaken will also be captured. The present review is intended to inform the development of an intervention which aims to reduce sedentary behaviour in non-ambulant young people with long-term disabilities. The targeted age range for the planned intervention includes young people aged 12–25 years. The present review will include research relating to a wide age range, from birth to 25 years. This extends the age range of the recent scoping review to include 18–25 year olds and summarises the evidence throughout children’s development. Covering this wide age range provides a broad perspective that allows an in-depth understanding of children’s behaviour, taking into consideration how sedentary behaviour changes throughout the journey from childhood into adulthood. This captures key transition points that may have an impact on sedentary behaviour such as leaving school and moving from children’s to adult’s health services.

The objectives of the review are to:

1. Identify all peer-reviewed journal articles relating to measurement of sedentary time, patterns of sedentary behaviour and interventions to decrease sedentary time or behaviours for non-ambulant children and young people.
2. Categorise the articles according to study design and four subquestions relating to (1) measurement, (2) patterns, (3) associated risks and (4) interventions to reduce sedentary time or behaviour for non-ambulant children and young people.
3. Critically appraise the quality of the articles using appropriate tools to evaluate the study design.
4. Produce a best evidence synthesis,^9 10^ which summarises the evidence for each subquestion and describes its cumulative strength, based on the hierarchy of evidence and the quality ratings assigned following critical appraisal. Identify knowledge gaps for this specific population.

The overarching research question is ‘What is the quality of the evidence relating to sedentary behaviour in non-ambulant children and young people?’ The sub-questions are:

1. Do validated measures of sedentary time or behaviour of non-ambulant children and young people exist?
2. Have patterns of sedentary time or behaviour of non-ambulant children and young people been described?
3. Have risks associated with sedentary time or behaviour been explored or quantified for non-ambulant children and young people?
4. What evidence-based interventions to reduce sedentary behaviour or decrease and break up sedentary time in this population have been evaluated and what is the strength of this evidence?

Grant and Booth^10^ have described the typology of the systematic search and review. They highlight how this type of review combines a systematic search method with a critical review analysis. This review typology is used to answer broad questions and often incorporates multiple study designs. The analysis results in a best evidence synthesis to describe what is known, recommendations for practice and limitations of the evidence. A systematic search and review typology is being adopted for this review because the topic is too broad to undertake a traditional systematic review, which should focus on only one specific question. The incorporation of several subquestions and the inclusion of studies from multiple paradigms with widely varying study designs makes a systematic review methodology inappropriate.^10^

## Methods and analysis

### Search strategy

Databases searched by Ganz *et al*,^3^ were CINAHL, MEDLINE, ERIC, EMBASE and SPORTDiscus. The electronic databases searched in this review include the following additional databases, on a variety of platforms to expand the breadth of the search: The Allied and Complementary Medicine Database (AMED), APA PsycINFO, Applied Social Sciences Index & Abstracts, Child development and adolescent studies, CINAHL Plus, Cochrane Library, EMBASE: Excerpta, ERIC, MEDLINE (R), Nursing and Allied Health Database, Scopus, SPORTDiscus and Web of Science.

The searches for this review will be limited to publication dates between January 1996 and September 2021, as the majority of the relevant publications identified by Ganz *et al*,^3^ were published after 2013 (72%), with the earliest included paper being published in 1996. Searches were undertaken by Ganz *et al*,^3^ in November 2018, so new papers published between November 2018 and September 2021 will be screened in this review. Reference lists of eligible articles will also be screened for any relevant articles that may have been missed in the database searches.

Traditional tools used to produce search strategies in systematic reviews include the PICO tool (Population, Intervention, Comparison, Outcome), which is ideally suited to quantitative studies and the SPIDER tool (Sample, Phenomenon of Interest, Design, Evaluation, Research type), which is suitable for qualitative and mixed-methods research.^11^ In this review, quantitative, qualitative, mixed-methods studies and reviews are all relevant. Therefore, the ‘Population, Concept and Context’ (PCC) framework recommended for use in scoping reviews, has been adopted.^12^ The PCC framework is more appropriate than the PICO tool to capture the wide range of study designs associated with multiple subquestions. The inclusion and exclusion criteria developed using the PCC framework is outlined in table 1 below.

**Table 1:** Inclusion and exclusion criteria

PCC framework element	Inclusion	Exclusion
**Population**	Participants with long-term physical disabilities. AND A proportion of the participants are non-ambulant. The proportion of non-ambulant participants is clear. For participants with cerebral palsy, children and young people in GMFCS levels IV–V are considered non-ambulant. AND Article includes participants under 25 years.	Participants with intellectual disabilities, visual or hearing impairments and no physical disability. OR Participants with a temporary lack of ambulation due to injury or short-term illness. OR The article includes ambulant participants only, or the proportion of non-ambulant participants is unclear. For participants with cerebral palsy, children and young people in GMFCS levels I–-III are considered ambulant. OR All participants in the article are 25 and over
**Concept**	Article explores measurement of, patterns of or risks related to sedentary behaviour. OR Article evaluates feasibility or effectiveness of an intervention to decrease or break up sedentary time. Any outcome measured in studies matching the inclusion criteria will be accepted.	Papers relating to physical activity that have not measured or explored patterns or correlates of sedentary time or behaviour specifically.
**Context**	Peer reviewed journal articles (quantitative, qualitative, mixed methods), scoping reviews, systematic reviews, theses / dissertations AND Published in the English language. AND Published between January 1996 and September 2021.	Grey literature, abstracts, conference proceedings, conference papers, conference review, protocols, commentaries, opinion articles, literature reviews (not systematic), narrative reviews, books or chapters. OR Published in languages other than English. OR Published before 1996 or after September 2021.

GMFCS, Gross Motor Function Classification System; PCC, Population, Concept, Context.

As quality rather than scope of the evidence is the focus of this review, grey literature has been excluded. The search strategy and examples of the search terms used in two of the databases (one which offers subject heading searches and a second which does not) are shown in table 2. Please refer to online supplemental file 1 for the full search strategy. Development of the search strategy terms was guided by those adopted by Ganz *et al*,^3^ however, additional terms have been included to ensure the search is specific to the overarching research question and the subquestions outlined above. Iterative cycles of trying different terms were carried out in order to decide on the terms that yielded the most appropriate results and to ensure the terms were suitable across the wide range of databases.

10.1136/bmjopen-2021-053077.supp1 Supplementary data

**Table 2:** Search strategy using PCC framework

Database	Population	Concept	Limits applied
**AMED—The Allied and Complementary Medicine Database** **Via EBSCOhost**	‘physical disabilit*’ (KW) OR ‘physically disabled’ (KW) OR ‘non-ambulant’ (KW) OR ‘non-ambulatory’ (KW) OR wheelchair* (KW) OR ‘limited mobility’ (KW) OR ‘immobil*’ (KW) OR ‘mobility impairment’ (KW) OR ‘neuromusculoskeletal disabilit*’ (KW) OR amputee* (KW) OR ‘cerebral pals*’ OR diplegi* (KW) OR quadripleg* (KW) OR tetrapleg* (KW) OR ‘muscular dystroph*’ OR ‘spina bifida’ (KW) OR ‘spinal dysraphism’ (KW) OR ‘neural tube defect*’ (KW) OR parapleg* (KW) paralys* (KW) OR ‘spinal cord injur*’ OR handicap* (KW) OR ‘hereditary motor sensory neuropath*’ (KW) OR ‘charcot-marie-tooth’ (KW) OR ‘spinal muscular atroph*’ (KW) AND minors (KW) OR pediatric (KW) OR paediatrics (KW) OR pediatric (KW) OR paediatric (KW) OR child (KW) OR children (KW) OR baby (KW) OR childhood (KW) OR babies (KW) OR infant (KW) OR infants (KW) OR infancy (KW) OR school (KW) OR preschool (KW) OR adolescen* (KW) OR teen* (KW) OR youth (KW) OR ‘young adult*’ (KW) OR ‘young person*’ (KW) OR ‘young people’ (KW) OR toddler* (KW) OR juvenile* (KW)	sedentary (KW) OR ‘screen time’ (KW) OR screentime (KW) OR ‘screen-time’ (KW) OR ‘screen use’ (KW) OR ‘screen exposure’ (KW) OR (television or TV) NEAR/3 watch* OR ‘physical inactivity’ (KW) OR ‘physically inactive’ (KW) OR sitting (KW)	Published date: 1996–2021 Language: English
**EMBASE: Excerpta Medica via Ovid**	‘physical disabilit*’ (KW) OR ‘physically disabled’ (KW) OR ‘non-ambulant’ (KW) OR ‘non-ambulatory’ (KW) OR wheelchair* (KW) OR ‘limited mobility’ (KW) OR ‘immobil*’ (KW) OR ‘mobility impairment’ (KW) OR ‘neuromusculoskeletal disability’ (KW) (NB wouldn’t accept ‘neuromusculoskeletal disabilit*’ OR amputee* (KW) OR ‘cerebral pals*’ OR diplegi* (KW) OR quadripleg* (KW) OR tetrapleg* (KW) OR ‘muscular dystroph*’ OR ‘spina bifida’ (KW) OR ‘spinal dysraphism’ (KW) OR ‘neural tube defect*’ (KW) OR parapleg* (KW) paralys* (KW) OR ‘spinal cord injur*’ OR handicap* (KW) OR ‘hereditary motor sensory neuropath*’ (KW) OR ‘charcot-marie-tooth’ (KW) OR ‘spinal muscular atroph*’ (KW) OR Physical disability (SH) (exp) OR Wheelchair (SH) (exp) OR Limited Mobility (SH) (exp) OR Cerebral palsy (SH) (exp) (KW) OR Amputee (SH) (exp) OR Muscular dystrophy (SH) (exp) OR Duchenne Muscular Dystrophy (SH) (exp) OR Spina Bifida (SH) exp) OR Spinal Dysraphism (SH) (exp) OR Neural Tube Defect (SH) (exp) OR Paraplegia (SH) (exp) OR Paralysis (SH) (exp) OR Spastic paraplegia (SH) (exp) OR Quadriplegia (SH) (exp) OR Spinal cord injury (SH) (exp) OR Hereditary Motor Sensory Neuropathy (SH) (exp) OR Muscular Atrophy, Spinal (SH) (exp) AND minors (KW) OR pediatric (KW) OR paediatrics (KW) OR pediatric (KW) OR paediatric (KW) OR child (KW) OR children (KW) OR baby (KW) OR childhood (KW) OR babies (KW) OR infant (KW) OR infants (KW) OR infancy (KW) OR school (KW) OR preschool (KW) OR adolescen* (KW) OR teen* (KW) OR youth (KW) OR ‘young adult*’ (KW) OR ‘young person*’ (KW) OR ‘young people’ (KW) OR toddler* (KW) OR juvenile* (KW) OR Young Adult (SH) (exp) OR Adolescent (SH) (exp) OR (SH) (exp) OR Infant (SH) (exp) OR Child (SH) (exp)	sedentary (KW) OR ‘screen time’ (KW) OR screentime (KW) OR ‘screen-time’ (KW) OR ‘screen use’ (KW) OR ‘screen exposure’ (KW) OR (television or TV) adj3 watch* OR ‘physical inactivity’ (KW) OR ‘physically inactive’ (KW) OR Sedentary lifestyle (SH) (exp) OR Sedentary time (SH) (exp) OR Screen time (SH) (exp) OR Television (SH) (exp) OR Television viewing (SH) (exp) OR Sitting (SH) (exp) OR Physical Inactivity (SH) (exp)	Publication year: 1996–2021 English language

NB where subject headings were not exploded, this is because this option was not available.

exp, exploded; KW, keyword in title or abstract; PCC, Population, Concept and Context; SH, subject heading.

Searches for each group of terms defining the ‘Population’ and the ‘Concept’ (table 1) will be conducted on separate lines in the databases, with individual terms combined as shown in table 2. The results from these searches will then be combined using AND. The limits outlined in table 2 were applied to the search results prior to exporting citations to Endnote reference management software.^13^

Final searches will be conducted in September 2021. Our planned timelines for the study are as follows: screening of papers for inclusion will be carried out between September and November 2021, appraisal of included articles and data extraction between December and April 2022 and production of the best evidence synthesis and writing of the review article between May and July 2022.

### Management of study records

All citations from the individual databases searched will be exported into a bibliographic management software programme.^13^ Records from the individual database searches will be combined and duplicates removed. The titles and abstracts will be screened for relevance by two independent reviewers using the inclusion and exclusion criteria outlined in table 1. Articles will be rated as relevant, not relevant or not known. Articles will be rated as not known if there is insufficient information in the abstract to decide its relevance, or if the reviewer is uncertain of whether or not it fits the inclusion criteria. Full-text articles will be obtained for all articles deemed to be ‘relevant’ or ‘not known’ following screening. Any discrepancies between the reviewers in relation to the inclusion of articles will be discussed following review of the full text and a consensus agreed. A third reviewer will be available to review full-text articles for which agreement cannot be reached between the first and second reviewers.

### Assessing quality of the evidence

The internal validity of each article will be assessed using an appropriate tool. Quality and risk of bias will therefore be assessed at study, but not outcome level. Full-text articles will be evaluated by two independent reviewers. An overall rating of the quality of each article (good, fair, poor or unclear) will be assigned by both reviewers, based on a risk of bias assessment using a collection of critical appraisal tools. If there are disagreements between the two reviewers that cannot be reconciled through discussion, the third reviewer will be consulted. Any articles rated as ‘unclear’ by either reviewer will also be discussed by the two independent reviewers and if required referred to the third reviewer to rate the quality of the article as good, fair or poor.

The Cochrane handbook explains that studies at high risk of bias should be given reduced weight in comparison to studies at low risk of bias.^14^ The flaws that exist in studies with a high risk of bias cannot be disregarded in the best evidence synthesis we will produce. It is important to find a balance between bias and precision. Including all studies in the review, regardless of their quality offers a high level of precision but serious risk of bias. Only including high-quality studies with low risk of bias reduces the precision of the evidence synthesis. The Cochrane Handbook suggests four strategies authors can select from to present their results meaningfully.^14^ These strategies relate to systematic reviews of interventions and therefore focus on management of quantitative data from randomised control trials in meta-analysis. The scoping review previously undertaken gives an indication of the designs of studies that are likely to be included in this review.^3^ Thirteen articles in the existing review included non-ambulant participants. Their designs were described as cross-sectional or cross-sectional survey, longitudinal, pre–post test, prospective cohort and validation studies. As they are not randomised control trials of interventions, the strategies recommended in the Cochrane handbook are therefore difficult to apply in this review. The strategy we will adopt is as follows; to minimise serious risk of bias, articles rated as being poor quality will be excluded from this review. Articles rated as good or fair quality will be accepted for data extraction and inclusion in the evidence synthesis but their quality rating will be taken into account.

Reviewers will independently categorise each article by sub question and design.

The work of Ganz *et al*^3^ gives an indication of the types of critical appraisal tools that will be required to assess risk of bias. The following critical appraisal tools will therefore be used to evaluate the quality of these studies:

1. The Quality Assessment Tool for Observational Cohort and Cross-Sectional Studies.^15^
2. The Quality Assessment Tool for Before–After (Pre–Post) Studies With No Control Group.^15^

If additional systematic reviews, randomised control trials, mixed-methods studies or qualitative studies are identified, the following tools will be used to assess quality and risk of bias:

1. The CASP Systematic Review Checklist.^16^
2. The Cochrane Collaboration’s tool for assessing risk of bias in randomised trials.^17^
3. The Mixed Methods Appraisal Tool.^18^
4. The CASP Qualitative Checklist.^16^

### Data extraction

One reviewer will undertake data extraction for included articles. The second reviewer will check the accuracy of this data extraction. Table 3 shows the data items which will be extracted from each article. If the data items listed below are not included in the article, the corresponding author will be contacted twice to request this information. If no response is received, it will be marked as unavailable.

**Table 3:** Data extraction items

Data item								
**Author (year) (ref)** **Country of origin**	Participants*	**Objectives**	**Design**	**Intervention (subquestion three studies only)**	**Methods†**	**Results (specific to sedentary behaviour)**	**Critical appraisal tool**	**Quality of article (good, fair, poor)**

*Participant data will include total number of participants, number of non-ambulant participants, diagnosis, age range, mean age and SD (if available). GMFCS level/level of impairment of participants if available.

†Methods will include type of measure or data collection method (eg, gas analysis, accelerometer make and model), patient report (survey, interview, validated patient-reported outcome measure), protocol (eg, placement of accelerometer, accelerometer cut point used, wear protocol), data reduction if applicable (eg, cut-points), outcome variable used in study if applicable (eg, sedentary time, sedentary behaviour (total or min/day)).

GMFCS, Gross Motor Function Classification System.

## Results

The search results will be presented in a Preferred Reporting Items for Systematic Reviews and Meta-Analyses (PRISMA) flowchart. The flowchart will show the number of articles identified, the number of duplications and articles included and excluded at each stage, the reasons for exclusions and number of articles excluded for each reason. Four tables will be produced to summarise the evidence from included articles, one for each of the sub questions, containing the data items described in table 3.

### Public and patient involvement

Three public and patient involvement representatives have reviewed this protocol and provided feedback. They will also follow the progress of the review, having oversight of and providing feedback on our processes of screening the papers, assessing quality of relevant articles and disseminating the results. One of these three representatives has also kindly agreed to act as the third reviewer.

### Best evidence synthesis

Slavin^9^ describes how producing a best evidence synthesis allows reviewers propose conclusions or hypotheses about what we can learn from a body of evidence in relation to a particular topic in situations where there are studies carried out by a variety of research teams, involving different populations and methods to examine the same phenomenon. There may also be a lack of consensus in the conclusions of these studies. The best evidence synthesis consists of a critical examination of evidence such as this, producing justified conclusions and transparently discussing any evidence that contradicts these conclusions.

In this review, the strength of the cumulative evidence for each subquestion will be described by summarising what is known, for which age group and diagnoses, and with what level of confidence (considering the hierarchy of evidence and quality rating). Diagnosis, age of onset and cognitive abilities of the children included in the research are likely to vary widely and will be an important consideration when summarising what is known. An assessment of the heterogeneity of the evidence and whether there is any potential for evidence synthesis will be undertaken and described. Cultural and economic differences between countries will be an important consideration in relation to the external validity or transferability of findings from individual studies. Any knowledge gaps will be identified and potential directions for future research will be discussed for each sub question.

## Ethics and dissemination

Ethical approval is not required to undertake this review. The review will be submitted for peer review in an appropriate journal.

## Supplementary Material

Author's manuscript
