# Supplementary material for: Sedentary behaviour in non-ambulant children and young people with physical disabilities: a systematic search and review protocol
Source: BMJ Open. 2021 Dec 3;11(12):e053077. doi: 10.1136/bmjopen-2021-053077 (PMC8647535; doi:10.1136/bmjopen-2021-053077)
Supplement: Supplementary data [file bmjopen-2021-053077supp001.pdf]

Sedentary Behaviour in Non-Ambulant Children and Young People with Physical Disabilities: A Systematic Search and Review Protocol

Search strategy developed using "Population, Concept, Context" framework.

Database	Population	Concept	Limits applied
**AMED - The Allied and Complementary Medicine Database Via EBSCOhost**	"physical disabilit*" (KW) OR "physically disabled" (KW) OR "non-ambulant" (KW) OR "non-ambulatory" (KW) OR wheelchair* (KW) OR "limited mobility" (KW) OR "immobil*"(KW) OR "mobility impairment" (KW) OR "neuromusculoskeletal disabilit*" (KW) OR amputee* (KW) OR "cerebral pals*" OR diplegi* (KW) OR quadripleg* (KW) OR tetrapleg* (KW) OR "muscular dystroph*" OR "spina bifida" (KW) OR "spinal dysraphism" (KW) OR "neural tube defect*" (KW) OR parapleg* (KW) paralys* (KW) OR "spinal cord injur*" OR handicap* (KW) OR "hereditary motor sensory neuropath*" (KW) OR "charcot-marie-tooth" (KW) OR "spinal muscular atroph*" (KW)<br><br>AND<br><br>minors (KW) OR pediatric (KW) OR paediatrics (KW) OR pediatric (KW) OR paediatric (KW) OR child (KW) OR children (KW) OR baby (KW) OR childhood (KW) OR babies (KW) OR infant (KW) OR infants (KW) OR infancy (KW) OR school (KW) OR preschool (KW) OR adolescen* (KW) OR teen* (KW) OR youth (KW) OR "young adult*" (KW) OR "young person*" (KW) OR "young people" (KW) OR toddler* (KW) OR juvenile* (KW)	sedentary (KW) OR "screen time" (KW) OR screentime (KW) OR "screen-time" (KW) OR "screen use" (KW) OR "screen exposure" (KW) OR (television or TV) NEAR/3 watch* OR "physical inactivity" (KW) OR "physically inactive" (KW) OR sitting (KW)	Published date: 1996 – 2021 Language: English
**Applied Social Sciences Index & Abstracts (ASSIA) Via ProQuest**			Publication date: 1996-2021 English language
**Child development and adolescent studies Via EBSCOhost**			Publication date: 1996 – 2021
**ERIC Via EBSCOhost**			Date Published: Jan 1996 – 2021 English Language
**SPORTDiscus Via EBSCOhost**			Publication Date: Jan 1996 – 2021 English Language
**APA PsycINFO Via Ovid**	"physical disabilit*" (KW) OR "physically disabled" (KW) OR "non-ambulant" (KW) OR "non-ambulatory" (KW) OR wheelchair* (KW) OR "limited mobility" (KW) OR "immobil*"(KW) OR "mobility impairment" (KW) OR "neuromusculoskeletal disability" (KW) (NB wouldn't accept "neuromusculoskeletal disabilit*") OR amputee*	sedentary (KW) OR "screen time" (KW) OR screentime (KW) OR "screen-time" (KW) OR "screen use" (KW) OR	Publication Year: 1996 – 2021 English language

	(KW) OR “cerebral pals*” OR diplegi* (KW) OR quadripleg* (KW) OR tetrapleg* (KW) OR “muscular dystroph*” OR “spina bifida” (KW) OR “spinal dysraphism” (KW) OR “neural tube defect*” (KW) OR parapleg* (KW) paralys* (KW) OR “spinal cord injur*” OR handicap* (KW) OR “hereditary motor sensory neuropath*” (KW) OR “charcot-marie-tooth” (KW) OR "spinal muscular atroph*" (KW)<br>OR “Physical disabilities (Attitudes toward)” (SH) (exp) OR Mobility aids (SH) (exp) OR Amputation (SH) (exp) OR Cerebral Palsy (SH) (exp) OR Quadriplegia (SH) (exp) OR Muscular Dystrophy (SH) (exp) OR Spina Bifida (SH) (exp) OR Paralysis (SH) (exp) OR Spinal Cord Injuries (SH) (exp)<br>OR Paragplegia (SH) (exp) OR Charcot-Marie-Tooth Disease (SH) (exp)<br><br>AND<br><br>minors OR pediatric OR paediatrics OR pediatrics OR paediatric OR child OR children OR baby OR childhood OR babies OR infant OR infants OR infancy OR school OR preschool OR adolescen* OR teen* OR youth OR "young adult*" OR "young person*" OR "young people" OR toddler* OR juvenile*	"screen exposure" (KW) OR (television or TV) adj3 watch* OR "physical inactivity" (KW) OR "physically inactive" (KW) OR sitting (KW) OR Sedentary behavior (SH) (exp) OR Television viewing (SH) (exp) OR Television (SH) (exp) OR Screen Time (SH) (exp)	
**CINAHL Plus via EBSCOhost**	“physical disabilit*” (KW) OR “physically disabled” (KW) OR “non-ambulant” (KW) OR “non-ambulatory” (KW) OR wheelchair* (KW) OR “limited mobility” (KW) OR “immobil*”(KW) OR “mobility impairment” (KW) OR “neuromusculoskeletal disabilit*” (KW) OR amputee* (KW) OR “cerebral pals*” OR diplegi* (KW) OR quadripleg* (KW) OR tetrapleg* (KW) OR “muscular dystroph*” OR “spina bifida” (KW) OR “spinal dysraphism” (KW) OR “neural tube defect*” (KW) OR parapleg* (KW) paralys* (KW) OR “spinal cord injur*” OR handicapped (KW) OR “hereditary motor sensory neuropath*” (KW) OR “charcot-marie-tooth” (KW) OR "spinal muscular atroph*" (KW) OR Immobility (SH) OR Wheelchairs (SH) (exp) OR Amputees (SH) (exp) OR Cerebral palsy (SH) OR Muscular Dystrophy (SH) (exp) OR Spina Bifida (SH) OR Neural Tube Defects (SH) (exp) OR Paraplegia (SH) (exp) OR Spastic Paraplegia, Hereditary (SH) OR Spinal Cord Injuries (SH) (exp) OR Quadriplegia (SH) (exp) OR Neuropathies, Hereditary motor and sensory (exp) OR Charcot-Marie-Tooth Disease (SH) OR Muscular Atrophy, Spinal (SH) (exp)<br><br>AND<br><br>minors (KW) OR pediatric (KW) OR paediatrics (KW) OR pediatric (KW) OR paediatric (KW) OR child (KW) OR	sedentary (KW) OR “screen time" (KW) OR screentime (KW) OR "screen-time" (KW) OR "screen use" (KW) OR "screen exposure" (KW) OR "(television or TV) near/3 watch* OR "physical inactivity" (KW) OR "physically inactive" (KW) OR Life Style, Sedentary (SH) (exp) OR Television (SH) OR Screen Time (SH) OR Sitting (SH)	Publication date: 1996 – 2021<br>Language: English

	children (KW) OR baby (KW) OR childhood (KW) OR babies (KW) OR infant (KW) OR infants (KW) OR infancy (KW) OR school (KW) OR preschool (KW) OR adolescen* (KW) OR teen* (KW) OR youth (KW) OR "young adult*" (KW) OR "young person*" (KW) OR "young people" (KW) OR toddler* (KW) OR juvenile* (KW) OR Infant, Newborn (SH) (exp) OR Minors (Legal) (SH) (exp) OR Child (SH) (exp) OR Adolescence (SH) (exp)		
**Cochrane Library**<br><br>**Via Cochrane library website**	Searched title and abstract only for:<br>“physical disability” OR “physical disabilities” OR “physically disabled” OR “non-ambulant” OR “non-ambulatory” OR wheelchair* OR “limited mobility” OR immobilit* OR “mobility impairment” OR “neuromusculoskeletal disabilit*” OR “neuromusculoskeletal disability” OR amputee* OR “cerebral palsy” OR diplegi* OR quadripleg* OR tetrapleg* OR “muscular dystrophy” OR “spina bifida” OR “spinal dysraphism” OR “neural tube defect” OR “neural tube defects” OR parapleg* OR paralys* OR “spinal cord injury” OR “spinal cord injuries” OR handicap* OR “hereditary motor sensory neuropathy” OR “hereditary motor sensory neuropathies” OR “charcot-marie-tooth” OR “spinal muscular atrophy” OR “spinal muscular atrophies” OR<br>Mobility Limitation (MeSH) (exp) OR Amputees (MeSH) (exp) OR<br>OR Wheelchairs (MeSH) (exp) OR<br>Cerebral Palsy (MeSH) (exp)<br>OR Muscular Dystrophies (MeSH) (exp)<br>OR Spinal Dysraphism (MeSH) (exp)<br>OR Neural Tube Defects (MeSH) (exp) OR Spinal Cord Injuries (MeSH) OR Paraplegia (MeSH) (exp) OR Quadriplegia (MeSH) (exp) OR Paralysis (MeSH) (exp) OR Spastic Paraplegia, Hereditary (MeSH) (exp) OR Hereditary Sensory and Motor Neuropathy (MeSH) (exp) OR Charvot-Marie-Tooth Disease (MeSH) (exp) OR Muscular Atrohy, Spinal (MeSH) (exp)<br><br>AND<br><br>minors OR pediatric OR paediatrics OR pediatrics OR paediatric OR child OR children OR baby OR childhood OR babies OR infant OR infants OR infancy OR school OR preschool OR adolescen* OR teen* OR youth OR "young adult" OR “young adults” OR "young person" OR "young people" OR toddler* OR juvenile*) OR Young Adult (MeSH) OR Adolescent (MeSH) OR (MeSH) OR Infant (MeSH) OR Child (MeSH)	Searched title and abstract only for:<br>sedentary (KW) OR “screen time" (KW) OR screentime (KW) OR "screen-time" (KW) OR "screen use" (KW) OR "screen exposure" (KW) OR (television or TV) near/3 watch* OR "physical inactivity" (KW) OR "physically inactive" (KW) OR Sedentary Behavior (MeSH) (exp) OR Television (MeSH) (exp) OR Sitting position (MeSH) (exp) OR Screen Time (MeSH) (exp)	Cochrane Library publication date:<br>Jan 1996 and 2021<br><br>Content type:<br>Cochrane reviews<br>Cochrane trials
**EMBASE: Excerpta Medica via Ovid**	“physical disabilit*” (KW) OR “physically disabled” (KW) OR “non-ambulant” (KW) OR “non-ambulatory” (KW) OR wheelchair* (KW) OR “limited mobility” (KW) OR	sedentary (KW) OR “screen time" (KW) OR screentime (KW)	Publication year: 1996 – 2021

	"immobil*"(KW) OR "mobility impairment" (KW) OR "neuromusculoskeletal disability" (KW) (NB wouldn't accept "neuromusculoskeletal disabilit*" OR amputee* (KW) OR "cerebral pals*" OR diplegi* (KW) OR quadripleg* (KW) OR tetrapleg* (KW) OR "muscular dystroph*" OR "spina bifida" (KW) OR "spinal dysraphism" (KW) OR "neural tube defect*" (KW) OR parapleg* (KW) paralys* (KW) OR "spinal cord injur*" OR handicap* (KW) OR "hereditary motor sensory neuropath*" (KW) OR "charcot-marie-tooth" (KW) OR "spinal muscular atroph*" (KW) OR Physical disability (SH) (exp) OR Wheelchair (SH) (exp) OR Limited Mobility (SH) (exp) OR Cerebral palsy (SH) (exp) (KW) OR Amputee (SH) (exp) OR Muscular dystrophy (SH) (exp) OR Duchenne Muscular Dystrophy (SH) (exp) OR Spina Bifida (SH) exp) OR Spinal Dysraphism (SH) (exp) OR Neural Tube Defect (SH) (exp) OR Paraplegia (SH) (exp) OR Paralysis (SH) (exp) OR Spastic paraplegia (SH) (exp) OR Quadriplegia (SH) (exp) OR Spinal cord injury (SH) (exp) OR Hereditary Motor Sensory Neuropathy (SH) (exp) OR Muscular Atrophy, Spinal (SH) (exp)<br><br>AND<br><br>minors (KW) OR pediatric (KW) OR paediatrics (KW) OR pediatric (KW) OR paediatric (KW) OR child (KW) OR children (KW) OR baby (KW) OR childhood (KW) OR babies (KW) OR infant (KW) OR infants (KW) OR infancy (KW) OR school (KW) OR preschool (KW) OR adolescen* (KW) OR teen* (KW) OR youth (KW) OR "young adult*" (KW) OR "young person*" (KW) OR "young people" (KW) OR toddler* (KW) OR juvenile* (KW) OR Young Adult (SH) (exp) OR Adolescent (SH) (exp) OR (SH) (exp) OR Infant (SH) (exp) OR Child (SH) (exp)	OR "screen-time" (KW) OR "screen use" (KW) OR "screen exposure" (KW) OR (television or TV) adj3 watch* OR "physical inactivity" (KW) OR "physically inactive" (KW) OR Sedentary lifestyle (SH) (exp) OR Sedentary time (SH) (exp) OR Screen time (SH) (exp) OR Television (SH) (exp) OR Television viewing (SH) (exp) OR Sitting (SH) (exp) OR Physical Inactivity (SH) (exp)	English language
**MEDLINE (R) Via Ovid**	"physical disabilit*" (KW) OR "physically disabled" (KW) OR "non-ambulant" (KW) OR "non-ambulatory" (KW) OR wheelchair* (KW) OR "limited mobility" (KW) OR "immobil*"(KW) OR "mobility impairment" (KW) OR "neuromusculoskeletal disabilit*" (KW) OR amputee* (KW) OR "cerebral pals*" OR diplegi* (KW) OR quadripleg* (KW) OR tetrapleg* (KW) OR "muscular dystroph*" OR "spina bifida" (KW) OR "spinal dysraphism" (KW) OR "neural tube defect*" (KW) OR parapleg* (KW) paralys* (KW) OR "spinal cord injur*" OR handicap* (KW) OR "hereditary motor sensory neuropath*" (KW) OR "charcot-marie-tooth" (KW) OR "spinal muscular atroph*" (KW) OR Wheelchairs (SH) (exp) OR Mobility Limitation (SH) (exp) OR Cerebral palsy (SH) (exp) (KW) OR Amputees (SH) (exp) OR Muscular dystrophies (SH) (exp) OR Spinal Dysraphism (SH) (exp) (KW) OR Neural Tube Defects (SH) (exp) OR	sedentary (KW) OR "screen time" (KW) OR screentime (KW) OR "screen-time" (KW) OR "screen use" (KW) OR "screen exposure" (KW) OR (television or TV) adj3 watch* OR "physical inactivity" (KW) OR "physically inactive" (KW) Sedentary Behavior (SH) (exp) OR Television (SH) (exp) OR Screen Time (SH) (exp) OR	Publication Year: 1996 – 2021 English language

	Paraplegia (SH) (exp) OR Paralysis (SH) (exp) OR Spastic paraplegia, hereditary (SH) (exp) OR Quadriplegia (SH) (exp) OR Spinal cord injuries (SH) (exp) OR Hereditary Sensory and Motor Neuropathy (SH) (exp) OR Charcot-Marie-Tooth Disease (SH) (exp) OR Muscular Atrophy, Spinal (SH) (exp)<br><br>AND<br><br>minors (KW) OR pediatric (KW) OR paediatrics (KW) OR pediatric (KW) OR paediatric (KW) OR child (KW) OR children (KW) OR baby (KW) OR childhood (KW) OR babies (KW) OR infant (KW) OR infants (KW) OR infancy (KW) OR school (KW) OR preschool (KW) OR adolescen* (KW) OR teen* (KW) OR youth (KW) OR "young adult*" (KW) OR "young person*" (KW) OR "young people" (KW) OR toddler* (KW) OR juvenile* (KW) OR Minors (SH) (exp) OR Child (SH) (exp) OR Child, Preschool (SH) (exp) OR Infant, Newborn (SH) (exp) OR Infant (SH) (exp) OR Adolescent (SH) (exp) OR Young Adult (SH) (exp)	Sitting Position (SH) (exp) OR	
**Nursing and Allied Health Database Via ProQuest**	"physical disabilit*" (KW) OR "physically disabled" (KW) OR "non-ambulant" (KW) OR "non-ambulatory" (KW) OR wheelchair* (KW) OR "limited mobility" (KW) OR "immobil*"(KW) OR "mobility impairment" (KW) OR "neuromusculoskeletal disabilit*" (KW) OR amputee* (KW) OR "cerebral pals*" OR diplegi* (KW) OR quadripleg* (KW) OR tetrapleg* (KW) OR "muscular dystroph*" OR "spina bifida" (KW) OR "spinal dysraphism" (KW) OR "neural tube defect*" (KW) OR parapleg* (KW) paralys* (KW) OR "spinal cord injur*" OR handicap* (KW) OR "hereditary motor sensory neuropath*" (KW) OR "charcot-marie-tooth" (KW) OR "spinal muscular atroph*" (KW) OR Neural Tube Defects (MeSH) OR Spastic Paraplegia, Hereditary (MeSH) OR Spina Bifida Occulta (MeSH) OR Amputees (MeSH) OR Quadriplegia (MeSH) OR Spina Bifida Cystica (MeSH) OR Charcot-Marie-Tooth Disease (MeSH) OR Mobility Limitation (MeSH) OR Paraplegia (MeSH) OR Muscular Dystrophies (MeSH) OR Spinal Cord Injuries (MeSH) OR Wheelchairs (MeSH) OR Muscular Atrophy, Spinal (MeSH) OR Spinal Dysraphism (MeSH) OR Hereditary Sensory & Motor Neuropathy (MeSH) OR Muscular Dystrophy, Duchenne (MeSH) OR Paralysis (MeSH) OR Cerebral Palsy (MeSH)<br><br>AND<br><br>minors (KW) OR pediatric (KW) OR paediatrics (KW) OR pediatric (KW) OR paediatric (KW) OR child (KW) OR children (KW) OR baby (KW) OR childhood (KW) OR	sedentary (KW) OR "screen time" (KW) OR screentime (KW) OR "screen-time" (KW) OR "screen use" (KW) OR "screen exposure" (KW) OR (television or TV) NEAR/3 watch* (KW) OR "physical inactivity" (KW) OR "physically inactive" (KW) OR sitting (KW) OR Sedentary lifestyle (MeSH) OR Television (MeSH)	Publication date: 01/01/1996 – 2021 English language only

	babies (KW) OR infant (KW) OR infants (KW) OR infancy (KW) OR school (KW) OR preschool (KW) OR adolescen* (KW) OR teen* (KW) OR youth (KW) OR "young adult*" (KW) OR "young person*" (KW) OR "young people" (KW) OR toddler* (KW) OR juvenile* (KW) OR Young Adult (MeSH) OR Adolescent (MeSH) OR (MeSH) OR Infant (MeSH) OR Child (MeSH)		
**Scopus**	Title and abstract search only (not key words).<br>“physical disability*” (KW) OR “physically disabled” (KW) OR “non-ambulant” (KW) OR “non-ambulatory” (KW) OR wheelchair* (KW) OR “limited mobility” (KW) OR “immobil*”(KW) OR “mobility impairment” (KW) OR “neuromusculoskeletal disabilit*” (KW) OR amputee* (KW) OR “cerebral pals*” OR diplegi* (KW) OR quadripleg* (KW) OR tetrapleg* (KW) OR “muscular dystroph*” OR “spina bifida” (KW) OR “spinal dysraphism” (KW) OR “neural tube defect*” (KW) OR parapleg* (KW) paralys* (KW) OR “spinal cord injur*” OR handicap* (KW) OR “hereditary motor sensory neuropath*” (KW) OR “charcot-marie-tooth” (KW) OR "spinal muscular atroph*" (KW)<br><br>AND<br><br>minors (KW) OR pediatric (KW) OR paediatrics (KW) OR pediatric (KW) OR paediatric (KW) OR child (KW) OR children (KW) OR baby (KW) OR childhood (KW) OR babies (KW) OR infant (KW) OR infants (KW) OR infancy (KW) OR school (KW) OR preschool (KW) OR adolescen* (KW) OR teen* (KW) OR youth (KW) OR "young adult*" (KW) OR "young person*" (KW) OR "young people" (KW) OR toddler* (KW) OR juvenile* (KW)	Title and abstract search only (not key words).<br>sedentary (KW) OR “screen time" (KW) OR screentime (KW) OR "screen-time" (KW) OR "screen use" (KW) OR "screen exposure" (KW) OR (television or TV) NEAR/3 watch* OR "physical inactivity" (KW) OR "physically inactive" (KW) OR sitting	Publication year: 1996-2021 English language
**Web of Science Core Collection**	Searched Title and Abstract for:<br>“physical disabilit*” OR “physically disabled” OR “non-ambulant” OR “non-ambulatory” OR wheelchair* OR “limited mobility” OR immobil* OR “mobility impairment” OR “neuromusculoskeletal disabilit*” OR amputee* OR “cerebral pals*” OR diplegi* OR quadripleg* OR tetrapleg* OR “muscular dystroph*” OR “spina bifida” OR “spinal dysraphism” OR “neural tube defect*” OR parapleg* OR paralys* OR “spinal cord injur*” OR handicap* OR “hereditary motor sensory neuropath*” OR “charcot-marie-tooth” OR “spinal muscular atroph*”<br><br>AND<br><br>minors OR pediatric OR paediatrics OR pediatrics OR paediatric OR child OR children OR baby OR childhood OR babies OR infant OR infants OR infancy OR school OR	Searched Title and Abstract for:<br>sedentary OR “screen time" OR screentime OR "screen-time" OR "screen use" OR "screen exposure" OR (television or TV) NEAR/3 watch* OR "physical inactivity" OR "physically inactive" OR sitting	Custom year range 1996-2021 English language

Bradbury M, *et al. BMJ Open* 2021; 11:e053077. doi: 10.1136/bmjopen-2021-053077

	preschool OR adolescen* OR teen* OR youth OR "young adult*" OR "young person*" OR "young people" OR toddler* OR juvenile*		
