# Supplementary material for: Sedentary behaviour in non-ambulant children and young people with physical disabilities: a systematic search and review protocol
Source: BMJ Open. 2021 Dec 3;11(12):e053077. doi: 10.1136/bmjopen-2021-053077 (PMC8647535; doi:10.1136/bmjopen-2021-053077)
Supplement: Author's manuscript [file bmjopen-2021-053077.draft_revisions.pdf]

BMJ Open is committed to open peer review. As part of this commitment we make the peer review history of every article we publish publicly available.

When an article is published we post the peer reviewers' comments and the authors' responses online. We also post the versions of the paper that were used during peer review. These are the versions that the peer review comments apply to.

The versions of the paper that follow are the versions that were submitted during the peer review process. They are not the versions of record or the final published versions. They should not be cited or distributed as the published version of this manuscript.

BMJ Open is an open access journal and the full, final, typeset and author-corrected version of record of the manuscript is available on our site with no access controls, subscription charges or pay-per-view fees (http://bmjopen.bmj.com).

If you have any questions on BMJ Open's open peer review process please email info.bmjopen@bmj.com

BMJ Open

Sedentary Behaviour in Non-Ambulant Children and Young People with Physical Disabilities: A Systematic Search and Review Protocol

Journal:	*BMJ Open*
Manuscript ID	bmjopen-2021-053077
Article Type:	Protocol
Date Submitted by the Author:	11-Jun-2021
Complete List of Authors:	Bradbury, Marilyn; University of Birmingham, School of Sport, Exercise and Rehabilitation Sciences; Birmingham Community Healthcare NHS Foundation Trust, Research and Innovation<br>O'Brien, Ciara; University of Surrey, School of Psychology<br>Giles, Nathan<br>Fenton, Sally; University of Birmingham, School of Sport, Exercise and Rehabilitation Sciences<br>Neilson, Sue; University of Birmingham, School of Nursing<br>Duda, Joan; University of Birmingham, School of Sport, Exercise & Rehabilitation Sciences
Keywords:	Developmental neurology & neurodisability < PAEDIATRICS, REHABILITATION MEDICINE, SOCIAL MEDICINE

SCHOLARONE™
Manuscripts

I, the Submitting Author has the right to grant and does grant on behalf of all authors of the Work (as defined in the below author licence), an exclusive licence and/or a non-exclusive licence for contributions from authors who are: i) UK Crown employees; ii) where BMJ has agreed a CC-BY licence shall apply, and/or iii) in accordance with the terms applicable for US Federal Government officers or employees acting as part of their official duties; on a worldwide, perpetual, irrevocable, royalty-free basis to BMJ Publishing Group Ltd ("BMJ") its licensees and where the relevant Journal is co-owned by BMJ to the co-owners of the Journal, to publish the Work in this journal and any other BMJ products and to exploit all rights, as set out in our licence.

The Submitting Author accepts and understands that any supply made under these terms is made by BMJ to the Submitting Author unless you are acting as an employee on behalf of your employer or a postgraduate student of an affiliated institution which is paying any applicable article publishing charge ("APC") for Open Access articles. Where the Submitting Author wishes to make the Work available on an Open Access basis (and intends to pay the relevant APC), the terms of reuse of such Open Access shall be governed by a Creative Commons licence – details of these licences and which Creative Commons licence will apply to this Work are set out in our licence referred to above.

Other than as permitted in any relevant BMJ Author's Self Archiving Policies, I confirm this Work has not been accepted for publication elsewhere, is not being considered for publication elsewhere and does not duplicate material already published. I confirm all authors consent to publication of this Work and authorise the granting of this licence.

For peer review only

TITLE: SEDENTARY BEHAVIOUR IN NON-AMBULANT CHILDREN AND YOUNG PEOPLE WITH PHYSICAL DISABILITIES: A SYSTEMATIC SEARCH AND REVIEW PROTOCOL

AUTHOR INFORMATION

Corresponding author: Marilyn Bradbury

Affiliations: School of Sport, Exercise and Rehabilitation Sciences, University of Birmingham, Birmingham Community Healthcare NHS Foundation Trust.

Postal address: Research and Innovation, Birmingham Community Healthcare NHS Foundation Trust, Trust Headquarters, 3 Priestley Wharf, 20 Holt Street, Birmingham, B7 4BN, United Kingdom

email: marilyn.bradbury1@nhs.net

ORCID ID: 0000-0001-6000-7229

Ciara O'Brien, School of Psychology, University of Surrey, Surrey, UK. ciara.o'brien@surrey.ac.uk

ORCID ID: 0000-0002-2215-6001

Nathan Giles, Lay member of the DoMore Study Steering Group. Birmingham, UK. NathanGiles20@outlook.com,

Sally A.M. Fenton, School of Sport, Exercise and Rehabilitation Sciences, University of Birmingham, Birmingham, UK S.A.M.Fenton@bham.ac.uk

Sue Neilson, School of Nursing, University of Birmingham, Birmingham, UK. S.J.Neilson@bham.ac.uk

Joan L. Duda, School of Sport, Exercise and Rehabilitation Sciences, University of Birmingham, Birmingham, UK J.L.DUDA@bham.ac.uk

Word count: 2, 383

For peer review only

ABSTRACT

Introduction:

Non-ambulant children and young people with physical disabilities are at high risk of experiencing negative health outcomes associated with sedentary time. A scoping review summarising evidence relating to sedentary behaviours of children with physical disabilities identified the need for validated methods of measuring physical activity of children who use wheelchairs and evaluation of interventions to reduce sedentary time. The review did not assess the quality of evidence relating to this topic, therefore its validity remains unclear. No reviews focussing on non-ambulant children and young people up to the age of 25 years have been undertaken.

The objectives of this review are to:

- Identify all peer reviewed articles relating to sedentary behaviour of non-ambulant children and young people.
- Categorise the articles according to study design and 4 sub questions relating to 1) measurement 2) patterns 3) associated risks and 4) interventions to reduce sedentary time or behaviour.
- Critically appraise quality of the articles using established critical appraisal tools.
- Summarise the evidence relating to sedentary behaviour of non-ambulant children and young people in a best evidence synthesis and identify knowledge gaps.

Methods and Analysis:

This protocol was developed using the PRISMA-P checklist. The research questions, inclusion / exclusion criteria and search terms have been developed using the "Population, Concept and Context" framework. Online databases were searched to identify peer reviewed articles published between 1996 and 2020. Two reviewers will screen, categorise and critically appraise the articles. Data extraction and analysis will be verified by the second reviewer.

Results will be reported as a best evidence synthesis, with reference to the PRISMA checklist.

Ethics and Dissemination:

Ethical approval is not required. The review will be submitted to an appropriate peer reviewed journal.

Registration

The review is registered on the Open Science Framework database.

KEYWORDS

Sedentary behaviour, physical disability, children, young people, wheelchair, non-ambulant, systematic search and review.

ARTICLE SUMMARY

STRENGTHS AND LIMITATIONS OF THIS STUDY

For peer review only - http://bmjopen.bmj.com/site/about/guidelines.xhtml

1. Detailed inclusion and exclusion criteria developed using the "Population, Concept and Context" framework.
2. A broad range of databases will be searched, crossing several disciplines.
3. Data extraction will be undertaken by one reviewer. The second reviewer will verify the accuracy of data extraction.
4. Published critical appraisal tools will be used to assess risk of bias for each included study, but risk of bias for outcomes across multiple studies will not be assessed.
5. An assessment of the potential for quantitative synthesis of evidence will be carried out.

INTRODUCTION

Sedentary behaviour is defined as "any waking behaviour characterized by an energy expenditure ≤1.5 metabolic equivalents (METs) while in a sitting or reclining posture",[1]. A large body of evidence from a variety of study designs suggests that reducing sedentary time is associated with lower health risk in children and young people aged 5-17 years,[2]. This body of evidence includes studies measuring both sedentary time and specific behaviours such as television watching. In children, time spent watching television has been associated with increased body mass index, reduced physical fitness, low self-esteem and reduced academic achievement,[2].

Children with disabilities are more sedentary than their able bodied peers,[3]. Lower levels of gross motor function,[4-6] and increasing age,[6-8] , are linked to increased sedentary behaviours in children with disabilities. Young people who are non-ambulant (i.e., unable to walk, or can walk short distances using a body support walker ["therapeutic walking"]), are therefore at greatest risk of experiencing negative health outcomes associated with sedentary time. Young people in this group may have conditions such as (but not limited to) cerebral palsy, spina bifida, muscular dystrophy, irreversible traumatic injuries or rare conditions.

A scoping review of sedentary behaviour and children with physical disabilities has recently summarised research relating to measurement of sedentary time, sedentary behaviour patterns, and interventions to reduce or break up time spent sedentary,[3]. The review identified that there is a lack of research evaluating interventions to decrease the sedentary behaviours of children with physical disabilities. In addition, the need for research that focuses on improving our understanding of sedentary behaviour patterns in children who are non-ambulant, including the validity of using accelerometers to measure sedentary time in this group, was recognised. To date, no reviews incorporating a quality assessment of research relating to this topic or focussing specifically on non-ambulant children and young people have been undertaken. This review builds on that of Ganz et al., (2020),[3] by focusing specifically on non-ambulant children and young people, incorporating critical evaluation of the quality of the evidence, considering risks associated with sedentary time or behaviour for this population and searching a wider range of databases. Contemporary work that has been published since the searches for the scoping review were undertaken will also be captured. The present review is intended to inform the development of an intervention which aims to reduce sedentary behaviour in non-ambulant young people with long-term disabilities. The targeted age range for the planned intervention includes young people aged 12 - 25 years. The present review will include research relating to a wide age range, from birth to 25 years. This extends the age range of the recent scoping review to include 18-25 year olds and summarises the evidence throughout

For peer review only - http://bmjopen.bmj.com/site/about/guidelines.xhtml

children's development; providing a broad perspective that allows an in depth understanding of children's behaviour prior to them reaching the age range our intervention targets.

The objectives of the review are to:

1) Identify all peer reviewed journal articles relating to measurement of sedentary time, patterns of sedentary behaviour and interventions to decrease sedentary time or behaviours for non-ambulant children and young people.
2) Categorise the articles according to study design and 4 sub questions relating to 1) measurement 2) patterns 3) associated risks and 4) interventions to reduce sedentary time or behaviour for non-ambulant children and young people.
3) Critically appraise the quality of the articles using appropriate tools to evaluate the study design.
4) Produce a best evidence synthesis to summarise the evidence and identify knowledge gaps for this specific population.

The overarching research question is "What is the quality of the evidence relating to sedentary behaviour in non-ambulant children and young people?". The sub-questions are:

1) Do validated measures of sedentary time or behaviour of non-ambulant children and young people exist?
2) Have patterns of sedentary time or behaviour of non-ambulant children and young people been described?
3) Have risks associated with sedentary time or behaviour been explored or quantified for non-ambulant children and young people?
4) What evidence-based interventions to reduce sedentary behaviour or decrease and break up sedentary time in this population have been evaluated and how effective are they?

A systematic search and review will be undertaken because the topic is too broad to undertake a traditional systematic review, which should focus on only one specific question. The incorporation of several sub-questions and the inclusion of studies from multiple paradigms with widely varying study designs makes a systematic review methodology inappropriate,[9].

For peer review only

METHODS AND ANALYSIS

Search Strategy

Databases searched by Ganz et al., (2020),[3] were CINAHL, MEDLINE, ERIC, EMBASE and SPORTDiscus. The electronic databases searched in this review include the following additional databases on a variety of platforms to expand the breadth of the search: The Allied and Complementary Medicine Database (AMED), APA PsycINFO, Applied Social Sciences Index & Abstracts (ASSIA), Child development and adolescent studies, CINAHL Plus, Cochrane Library, EMBASE: Excerpta, ERIC, MEDLINE (R), Nursing and Allied Health Database, Scopus, SPORTDiscus and Web of Science.

The searches for this review have been limited to publication dates between January 1996 – December 2020, as the majority of the relevant publications identified by Ganz et al.,[3] were published after 2013 (72%), with the earliest included paper being published in 1996. Searches were

For peer review only - http://bmjopen.bmj.com/site/about/guidelines.xhtml

undertaken by Ganz et al.,[3] in November 2018, so new papers published between November 2018 and December 2020 will be screened in this review. Reference lists of eligible articles will also be screened for any relevant articles that may have been missed in the database searches.

Traditional tools used to produce search strategies in systematic reviews include the PICO tool (which is ideally suited to quantitative studies) and the SPIDER tool (which is suitable for qualitative and mixed methods research),[10]. In this review, quantitative, qualitative, mixed methods studies and reviews are all relevant. Therefore, the "Population, Concept and Context" (PCC) framework recommended for use in scoping reviews, has been adopted,[11]. The PCC framework is more appropriate than the PICO tool to capture the wide range of study designs associated with multiple sub-questions. The inclusion and exclusion criteria developed using the PCC framework is outlined in Table 1 below.

Table 1 Inclusion and exclusion criteria. PCC = Population, Concept, Context. GMFCS = Gross Motor Function Classification System.

PCC framework element	Inclusion	Exclusion
**Population**	Participants with long term physical disabilities.<br>AND<br>A proportion of the participants are non-ambulant. The proportion of non-ambulant participants is clear.<br>For participants with cerebral palsy, children and young people in Gross Motor Function Classification System (GMFCS) levels IV-V are considered non-ambulant.<br>AND<br>Article includes participants under 25 years.	Participants with intellectual disabilities, visual or hearing impairments and no physical disability.<br>OR<br>Participants with a temporary lack of ambulation due to injury or short-term illness.<br>OR<br>The article includes ambulant participants only, or the proportion of non-ambulant participants is unclear.<br>For participants with cerebral palsy, children and young people in GMFCS levels I-III are considered ambulant.<br>OR<br>All participants in the article are 25 and over
**Concept**	Article explores measurement of, patterns of, or risks related to sedentary behaviour.<br>OR<br>Article evaluates feasibility or effectiveness of an intervention to decrease or break up sedentary time.	Papers relating to physical activity that have not measured or explored patterns or correlates of sedentary time or behaviour specifically.

For peer review only - http://bmjopen.bmj.com/site/about/guidelines.xhtml

	NB any outcome measured in studies matching the inclusion criteria will be accepted.	
**Context**	Peer reviewed journal articles (quantitative, qualitative, mixed methods), scoping reviews, systematic reviews, theses / dissertations<br><br>AND<br><br>Published in the English language.<br><br>AND<br><br>Published between January 1996 and November / December 2020 (see supplemental material for exact database search dates).	Grey literature, conference abstracts, conference proceedings, conference papers, conference review, protocols, commentaries, opinion articles, literature reviews (not systematic), narrative reviews, books or chapters.<br><br>OR<br><br>Published in languages other than English.<br><br>OR<br><br>Published before 1996 or after September 2020.

As quality rather than scope of the evidence is the focus of this review, grey literature has been excluded. The search strategy and examples of the search terms used in two of the databases (one which offers subject heading searches and a second which does not) are shown in Table 2. Please refer to the supplementary material for the full search strategy. Development of the search strategy terms was guided by those adopted by Ganz et al., (2020),[3], however, additional terms have been included to ensure the search is specific to the overarching research question and the sub-questions outlined above.

Table 2. Search strategy using PCC framework. exp = exploded; SH = subject heading; KW = keyword in title or abstract. NB where subject headings were not exploded, this is because this option was not available.

Database	Population	Concept	Limits applied
**AMED - The Allied and Complementary Medicine Database**<br><br>**Via EBSCOhost**<br><br>**Search date 20/11/2020**	"physical disabilit*" (KW) OR "physically disabled" (KW) OR "non-ambulant" (KW) OR "non-ambulatory" (KW) OR wheelchair* (KW) OR "limited mobility" (KW) OR "immobil*"(KW) OR "mobility impairment" (KW) OR "neuromusculoskeletal disabilit*" (KW) OR amputee* (KW) OR "cerebral pals*" OR diplegi* (KW) OR quadripleg* (KW) OR tetrapleg* (KW) OR "muscular dystroph*" OR "spina bifida" (KW) OR "spinal dysraphism" (KW) OR "neural tube defect*" (KW) OR parapleg* (KW) paralys* (KW) OR "spinal cord injur*" OR handicap* (KW) OR "hereditary motor sensory	sedentary (KW) OR "screen time" (KW) OR screentime (KW) OR "screen-time" (KW) OR "screen use" (KW) OR "screen exposure" (KW) OR (television or TV) NEAR/3 watch* OR "physical inactivity" (KW) OR	Published date: 1996 – 2020 Language: English

For peer review only - http://bmjopen.bmj.com/site/about/guidelines.xhtml

	neuropath*" (KW) OR "charcot-marie-tooth" (KW) OR "spinal muscular atroph*" (KW)<br><br>AND<br><br>minors (KW) OR pediatric (KW) OR paediatrics (KW) OR pediatric (KW) OR paediatric (KW) OR child (KW) OR children (KW) OR baby (KW) OR childhood (KW) OR babies (KW) OR infant (KW) OR infants (KW) OR infancy (KW) OR school (KW) OR preschool (KW) OR adolescen* (KW) OR teen* (KW) OR youth (KW) OR "young adult*" (KW) OR "young person*" (KW) OR "young people" (KW) OR toddler* (KW) OR juvenile* (KW)	"physically inactive" (KW) OR sitting (KW)	
**EMBASE: Excerpta Medica via Ovid**<br><br>**1974 to Nov 23rd 2020**<br><br>**Search date 25/11/2020**	"physical disabilit*" (KW) OR "physically disabled" (KW) OR "non-ambulant" (KW) OR "non-ambulatory" (KW) OR wheelchair* (KW) OR "limited mobility" (KW) OR "immobil*"(KW) OR "mobility impairment" (KW) OR "neuromusculoskeletal disability" (KW) (NB wouldn't accept "neuromusculoskeletal disabilit*" OR amputee* (KW) OR "cerebral pals*" OR diplegi* (KW) OR quadripleg* (KW) OR tetrapleg* (KW) OR "muscular dystroph*" OR "spina bifida" (KW) OR "spinal dysraphism" (KW) OR "neural tube defect*" (KW) OR parapleg* (KW) paralys* (KW) OR "spinal cord injur*" OR handicap* (KW) OR "hereditary motor sensory neuropath*" (KW) OR "charcot-marie-tooth" (KW) OR "spinal muscular atroph*" (KW) OR Physical disability (SH) (exp) OR Wheelchair (SH) (exp) OR Limited Mobility (SH) (exp) OR Cerebral palsy (SH) (exp) (KW) OR Amputee (SH) (exp) OR Muscular dystrophy (SH) (exp) OR Duchenne Muscular Dystrophy (SH) (exp) OR Spina Bifida (SH) exp) OR Spinal Dysraphism (SH) (exp) OR Neural Tube Defect (SH) (exp) OR Paraplegia (SH) (exp) OR Paralysis (SH) (exp) OR Spastic paraplegia (SH) (exp) OR Quadriplegia (SH) (exp) OR Spinal cord injury (SH) (exp) OR Hereditary Motor Sensory Neuropathy (SH) (exp) OR Muscular Atrophy, Spinal (SH) (exp)<br><br>AND<br><br>minors (KW) OR pediatric (KW) OR paediatrics (KW) OR pediatric (KW) OR paediatric (KW) OR child (KW) OR children (KW) OR baby (KW) OR childhood (KW) OR babies (KW) OR infant (KW) OR infants (KW) OR infancy (KW) OR school (KW) OR preschool (KW) OR adolescen*	sedentary (KW) OR "screen time" (KW) OR screentime (KW) OR "screen-time" (KW) OR "screen use" (KW) OR "screen exposure" (KW) OR (television or TV) adj3 watch* OR "physical inactivity" (KW) OR "physically inactive" (KW) OR Sedentary lifestyle (SH) (exp) OR Sedentary time (SH) (exp) OR Screen time (SH) (exp) OR Television (SH) (exp) OR Television viewing (SH) (exp) OR Sitting (SH) (exp) OR Physical Inactivity (SH) (exp)	Publication year: 1996 – current English language

For peer review only - http://bmjopen.bmj.com/site/about/guidelines.xhtml

	(KW) OR teen* (KW) OR youth (KW) OR "young adult*" (KW) OR "young person*" (KW) OR "young people" (KW) OR toddler* (KW) OR juvenile* (KW) OR Young Adult (SH) (exp) OR Adolescent (SH) (exp) OR (SH) (exp) OR Infant (SH) (exp) OR Child (SH) (exp)		

Searches for each group of terms defining the "Population" and the "Concept" (Table 1) were conducted on separate lines in the databases, with individual terms combined as shown in Table 2. The results from these searches were then be combined using AND. The limits outlined in Table 2 were applied to the search results prior to exporting citations to Endnote reference management software,[12].

Preliminary searches were carried out Oct-Nov 2020 and final searches between November and December 2020 (see supplementary material for exact search date for each database). Screening of papers will commence in March 2021.

Management of Study Records

All citations from the individual databases searched will be exported into a bibliographic management software program,[12]. Records from the individual database searches will be combined and duplicates removed. The titles and abstracts will be screened for relevance by two independent reviewers using the inclusion and exclusion criteria outlined in Table 1. Articles will be rated as relevant, not relevant, or not known. Articles will be rated as not known if there is insufficient information in the abstract to decide its relevance, or if the reviewer is uncertain of whether or not it fits the inclusion criteria. Full text articles will be obtained for all articles deemed to be "relevant" or "not known" following screening. Any discrepancies between the reviewers in relation to the inclusion of articles will be discussed following review of the full text and a consensus agreed. A third reviewer will be available to review full text articles for which agreement cannot be reached between the first and second reviewers.

Assessing quality of the evidence.

The internal validity of each article will be assessed using an appropriate tool. Quality and risk of bias will, therefore be assessed at study, but not outcome level. Full text articles will be evaluated by two independent reviewers. An overall rating of the quality of each article (good, fair, poor or unclear) will be assigned by both reviewers, based on a risk of bias assessment using a collection of critical appraisal tools. If there are disagreements between the two reviewers that cannot be reconciled through discussion, the third reviewer will be consulted. Any articles rated as "unclear" by either reviewer will also be discussed by the two independent reviewers and if required referred to the third reviewer to rate the quality of the article as good, fair or poor.

The Cochrane handbook explains that studies at high risk of bias should be given reduced weight in comparison to studies at low risk of bias,[13]. The flaws that exist in studies with a high risk of bias cannot be disregarded in the best evidence synthesis we will produce. It is important to find a balance between bias and precision. Including all studies in the review, regardless of their quality offers a high level of precision but serious risk of bias. Only including high quality studies with

For peer review only - http://bmjopen.bmj.com/site/about/guidelines.xhtml

low risk of bias reduces the precision of the evidence synthesis. The Cochrane Handbook suggests four strategies authors can select from to present their results meaningfully,[13]. These strategies relate to systematic reviews of interventions and therefore focus on management of quantitative data from randomised control trials in meta-analysis. The scoping review previously undertaken gives an indication of the designs of studies that are likely to be included in this review,[3]. Thirteen articles in the existing review included non-ambulant participants. Their designs were described as: cross-sectional or cross-sectional survey, longitudinal, pre-post test, prospective cohort and validation studies. As they are not randomised control trials of interventions, the strategies recommended in the Cochrane handbook are therefore difficult to apply in this review. The strategy we will adopt is as follows; to minimise serious risk of bias, articles rated as being poor quality will be excluded from this review. Articles rated as good or fair quality will be accepted for data extraction and inclusion in the evidence synthesis but their quality rating will be taken into account.

Reviewers will independently categorise each article by sub question and design.

The work of Ganz et al.,[3] gives an indication of the types of critical appraisal tools that will be required to assess risk of bias. The following critical appraisal tools will therefore be used to evaluate the quality of these studies:

The Quality Assessment Tool for Observational Cohort and Cross-Sectional Studies,[14]

The Quality Assessment Tool for Before-After (Pre-Post) Studies With No Control Group,[14]

If additional systematic reviews, randomised control trials, mixed methods studies or qualitative studies are identified, the following tools will be used to assess quality and risk of bias:

The CASP Systematic Review Checklist,[15]

The Cochrane Collaboration's tool for assessing risk of bias in randomised trials,[16]

The Mixed Methods Appraisal Tool,[17]

The CASP Qualitative Checklist,[15]

Data Extraction

One reviewer will undertake data extraction for included articles. The second reviewer will check the accuracy of this data extraction. Table 3 shows the data items which will be extracted from each article. If the data items listed below are not included in the article, the corresponding author will be contacted twice to request this information. If no response is received, it will be marked as unavailable.

Table 3. Data extraction items.

Data Item								
Author (year) (ref)	Participants*	Objectives	Design	Intervention (Sub-quesiton 3	Methods**	Results (specific to	Critical appraisal tool	Quality of article (good,

For peer review only - http://bmjopen.bmj.com/site/about/guidelines.xhtml

Country of origin?				studies only)		sedentary behaviour)		fair, poor)

*Participant data will include total number of participants, number of non-ambulant participants, diagnosis, age range, mean age and standard deviation. GMFCS level / level of impairment of participants if available.
**Methods will include type of measure or data collection method (e.g. gas analysis, accelerometer make and model, patient report (survey, interview, validated patient reported outcome measure), protocol (e.g. placement of accelerometer, accelerometer cut point used, wear protocol), data reduction if applicable (e.g. cut-points), outcome variable used in study if applicable (e.g. Sedentary time, sedentary behaviour (total or min/day)

Results
The search results will be presented in a PRISMA flowchart. The flowchart will show the number of articles identified, the number of duplications and articles included and excluded at each stage, the reasons for exclusions and number of articles excluded for each reason. Four tables will be produced to summarise the evidence from included articles, one for each of the sub questions, containing the data items described in Table 3.

Public and Patient Involvement

Three public and patient involvement representatives have reviewed this protocol and provided feedback. They will also follow the progress of the review, having oversight of and providing feedback on our processes of screening the papers, assessing quality of relevant articles and disseminating the results. One of these three representatives has also kindly agreed to act as the third reviewer.

Best evidence synthesis

The strength of the cumulative evidence for each sub question will be described by summarising what is known, for which age group and diagnoses, and with what level of confidence (considering the hierarchy of evidence and quality rating). Diagnosis, age of onset and cognitive abilities of the children included in the research is likely to vary widely and will be an important consideration when summarising what is known. An assessment of the heterogeneity of the evidence and whether there is any potential for evidence synthesis will be undertaken and described. Cultural and economic differences between countries will be an important consideration in relation to the external validity or transferability of findings from individual studies. Any knowledge gaps will be identified and potential directions for future research will be discussed for each sub question.

ETHICS AND DISSEMINATION

Ethical approval is not required to undertake this review. The review will be submitted for peer review in an appropriate journal.

For peer review only - http://bmjopen.bmj.com/site/about/guidelines.xhtml

REGISTRATION

The review has been registered on the Open Science Framework database. DOI: https://doi.org/10.17605/OSF.IO/SQXJB. Any protocol amendments will be recorded in the Open Science Framework database.

ACKNOWLEDGMENTS

The authors would like to thank public and patient involvement representatives Roger Leek and Jacqueline Hawkins, and librarians Lynne Harris and Abimbola Alayo for their contributions.

CONTRIBUTORSHIP STATEMENT

Authors and contributors listed in the acknowledgements made the following contributions:
Marilyn Bradbury: Conceptualisation, methodology, writing – original draft, writing - review and editing, visualisation, project administration, funding acquisition.
Dr Ciara O'Brien: Methodology, writing - review and editing, project administration.
Nathan Giles: Writing – review and editing, project administration.
Dr. Sally Fenton: Conceptualisation, methodology, writing - review and editing, visualisation.
Dr. Sue Neilson: Conceptualisation, methodology, writing - review and editing, visualisation.
Prof Joan Duda: Conceptualisation, methodology, writing - review and editing, visualisation.
Public and patient involvement representatives, Roger Leek and Jacqueline Hawkins: Writing – review and editing.

COMPETING INTERESTS STATEMENT

All authors have completed the ICMJE uniform disclosure form at www.icmje.org/coi_disclosure.pdf and declare: Marilyn Bradbury had financial support from the National Institute of Health Research for the submitted work; no financial relationships with any organisations that might have an interest in the submitted work in the previous three years; no other relationships or activities that could appear to have influenced the submitted work.

FUNDING

This work was supported by the National Institute for Health Research (NIHR), grant number ICA-CDRF-2018-04-ST2-021. The funder has not been involved in the development of this protocol.

REFERENCES

1. Sedentary Behaviour Research Network. Letter to the Editor: Standardized use of the terms "sedentary" and "sedentary behaviours". *Appl Physiol Nutr Metab* Online [eLetter]. 27 April 2012. https://doi.org/10.1139/h2012-024.
2. Tremblay MS, LeBlanc AG, Kho ME, et al. Systematic review of sedentary behaviour and health indicators in school-aged children and youth. *Int J Behav Nutr Phys Act*. 2011;8(1):98.
3. Ganz F, Hammam N, Pritchard L. Sedentary behavior and children with physical disabilities: a scoping review. *Disabil Rehabil*. 2020:1-13.
4. Oftedal S, Bell KL, Davies PS, et al. Sedentary and Active Time in Toddlers with and without Cerebral Palsy. *Med Sci Sports Exerc*. 2015;47(10):2076-83.
5. Shkedy Rabani A, Harries N, Namoora I, et al. Duration and patterns of habitual physical activity in adolescents and young adults with cerebral palsy. *Dev Med Child Neurol*. 2014;56(7):673-80.

For peer review only - http://bmjopen.bmj.com/site/about/guidelines.xhtml

6. Keawutan P, Bell KL, Oftedal S, et al. Longitudinal physical activity and sedentary behaviour in preschool-aged children with cerebral palsy across all functional levels. *Dev Med Child Neurol*. 2017.
7. Maher A, Williams T, Olds T, et al. Physical and sedentary activity in adolescents with cerebral palsy. *Dev Med Child Neurol*. 2007;49(6):450.
8. Luther BL. Food and physical activity choices to prevent overweight in children with spina bifida. *Dissertation Abstracts International: Section B: The Sciences and Engineering*. 2010;71(6-B):3597.
9. Grant MJ, Booth A. A typology of reviews: an analysis of 14 review types and associated methodologies. *Health Info Libr J*. 2009;26(2):91-108.
10. Cooke A, Smith D, Booth A. Beyond PICO: the SPIDER tool for qualitative evidence synthesis. *Qual Health Res*. 2012;22(10):1435-43.
11. The Joanna Briggs Institute. The Joanna Briggs Institute reviewers' manual 2015: methodology for JBI scoping reviews: The Joanna Briggs Institute 2015: 10-11.
12. The Endnote Team. EndNote. EndNote X7 ed. Philadelphia, PA: Clarivate; 2013.
13. Boutron I, Page M, Higgins J, et al. Considering bias and conflicts of interest among the included studies. In: Higgins J, Thomas J, Chandler J, et al., eds. Cochrane Handbook for Systematic Reviews of Interventions Version 62 (updated February 2021): Cochrane 2021.
14. National Heart Lung and Blood Institute. Study Quality Assessment Tools: U.S Department of Health& Human Services; 2017 [Available from: https://www.nhlbi.nih.gov/health-topics/study-quality-assessment-tools. Date Accessed 01/04/2021.
15. Critical Appraisal Skills Programme. CASP Checklists: CASP UK; 2020 [Available from: http://www.casp-uk.net/casp-tools-checklists. Date Accessed 01/04/2021.
16. Higgins JPT, Altman DG, Gøtzsche PC, et al. The Cochrane Collaboration’s tool for assessing risk of bias in randomised trials. *British Medical Journal*. 2011;343:d5928.
17. Hong QN, Fàbregues Feijóo S, Bartlett G, et al. The Mixed Methods Appraisal Tool (MMAT) version 2018 for information professionals and researchers. *Education for information*. 2018.

EXCLUSIVE LICENCE

I, the Submitting Author has the right to grant and does grant on behalf of all authors of the Work (as defined in the below author licence), an exclusive licence and/or a non-exclusive licence for contributions from authors who are: i) UK Crown employees; ii) where BMJ has agreed a CC-BY licence shall apply, and/or iii) in accordance with the terms applicable for US Federal Government officers or employees acting as part of their official duties; on a worldwide, perpetual, irrevocable, royalty-free basis to BMJ Publishing Group Ltd (“BMJ”) its licensees and where the relevant Journal is co-owned by BMJ to the co-owners of the Journal, to publish the Work in BMJ Open and any other BMJ products and to exploit all rights, as set out in our licence.

The Submitting Author accepts and understands that any supply made under these terms is made by BMJ to the Submitting Author unless you are acting as an employee on behalf of your employer or a postgraduate student of an affiliated institution which is paying any applicable article publishing charge (“APC”) for Open Access articles. Where the Submitting Author wishes to make the Work available on an Open Access basis (and intends to pay the relevant APC), the terms of reuse of such Open Access shall be governed by a Creative Commons licence – details of these licences and which Creative Commons licence will apply to this Work are set out in our licence referred to above.

For peer review only - http://bmjopen.bmj.com/site/about/guidelines.xhtml

Sedentary Behaviour in Non-Ambulant Children and Young People with Physical Disabilities: A Systematic Search and Review Protocol

Search strategy developed using "Population, Concept, Context" framework including dates for individual database searches.

Database	Population	Concept	Limits applied
**AMED - The Allied and Complementary Medicine Database Via EBSCOhost**<br><br>**Search date 20/11/2020**	"physical disabilit*" (KW) OR "physically disabled" (KW) OR "non-ambulant" (KW) OR "non-ambulatory" (KW) OR wheelchair* (KW) OR "limited mobility" (KW) OR "immobil*"(KW) OR "mobility impairment" (KW) OR "neuromusculoskeletal disabilit*" (KW) OR amputee* (KW) OR "cerebral pals*" OR diplegi* (KW) OR quadripleg* (KW) OR tetrapleg* (KW) OR "muscular dystroph*" OR "spina bifida" (KW) OR "spinal dysraphism" (KW) OR "neural tube defect*" (KW) OR parapleg* (KW) paralys* (KW) OR "spinal cord injur*" OR handicap* (KW) OR "hereditary motor sensory neuropath*" (KW) OR "charcot-marie-tooth" (KW) OR "spinal muscular atroph*" (KW)<br><br>AND<br><br>minors (KW) OR pediatric (KW) OR paediatrics (KW) OR pediatric (KW) OR paediatric (KW) OR child (KW) OR children (KW) OR baby (KW) OR childhood (KW) OR babies (KW) OR infant (KW) OR infants (KW) OR infancy (KW) OR school (KW) OR preschool (KW) OR adolescen* (KW) OR teen* (KW) OR youth (KW) OR "young adult*" (KW) OR "young person*" (KW) OR "young people" (KW) OR toddler* (KW) OR juvenile* (KW)	sedentary (KW) OR "screen time" (KW) OR screentime (KW) OR "screen-time" (KW) OR "screen use" (KW) OR "screen exposure" (KW) OR (television or TV) NEAR/3 watch* OR "physical inactivity" (KW) OR "physically inactive" (KW) OR sitting (KW)	Published date: 1996 – 2020<br>Language: English
**Applied Social Sciences Index & Abstracts (ASSIA) Via ProQuest**<br><br>**Search date 03/12/2020**			Publication date: 1996-2020<br>English language
**Child development and adolescent studies Via EBSCOhost**<br><br>**Search date 30/11/2020**			Publication date: 1996 – 2020
**ERIC Via EBSCOhost**<br><br>**Search date 02/12/2020**			Date Published: Jan 1996 – 2020<br>English Language
**SPORTDiscus Via EBSCOhost**<br><br>**Search date 02/12/2020**			Publication Date: Jan 1996 – 2020<br>English Language

For peer review only - http://bmjopen.bmj.com/site/about/guidelines.xhtml

**APA PsycINFO 1967 to November week 3 2020 Via Ovid**<br><br>**Search date 25/11/2020**	"physical disabilit*" (KW) OR "physically disabled" (KW) OR "non-ambulant" (KW) OR "non-ambulatory" (KW) OR wheelchair* (KW) OR "limited mobility" (KW) OR "immobil*"(KW) OR "mobility impairment" (KW) OR "neuromusculoskeletal disability" (KW) (NB wouldn't accept "neuromusculoskeletal disabilit*") OR amputee* (KW) OR "cerebral pals*" OR diplegi* (KW) OR quadripleg* (KW) OR tetrapleg* (KW) OR "muscular dystroph*" OR "spina bifida" (KW) OR "spinal dysraphism" (KW) OR "neural tube defect*" (KW) OR parapleg* (KW) paralys* (KW) OR "spinal cord injur*" OR handicap* (KW) OR "hereditary motor sensory neuropath*" (KW) OR "charcot-marie-tooth" (KW) OR "spinal muscular atroph*" (KW)<br>OR "Physical disabilities (Attitudes toward)" (SH) (exp) OR Mobility aids (SH) (exp) OR Amputation (SH) (exp) OR Cerebral Palsy (SH) (exp) OR Quadriplegia (SH) (exp) OR Muscular Dystrophy (SH) (exp) OR Spina Bifida (SH) (exp) OR Paralysis (SH) (exp) OR Spinal Cord Injuries (SH) (exp)<br>OR Paragplegia (SH) (exp) OR Charcot-Marie-Tooth Disease (SH) (exp)<br><br>AND<br><br>minors OR pediatric OR paediatrics OR pediatrics OR paediatric OR child OR children OR baby OR childhood OR babies OR infant OR infants OR infancy OR school OR preschool OR adolescen* OR teen* OR youth OR "young adult*" OR "young person*" OR "young people" OR toddler* OR juvenile*	sedentary (KW) OR "screen time" (KW) OR screentime (KW) OR "screen-time" (KW) OR "screen use" (KW) OR "screen exposure" (KW) OR (television or TV) adj3 watch* OR "physical inactivity" (KW) OR "physically inactive" (KW) OR sitting (KW) OR Sedentary behavior (SH) (exp) OR Television viewing (SH) (exp) OR Television (SH) (exp) OR Screen Time (SH) (exp)	Publication Year: 1996 – current English language
**CINAHL Plus via EBSCOhost**<br><br>**Search date 19/11/2020**	"physical disabilit*" (KW) OR "physically disabled" (KW) OR "non-ambulant" (KW) OR "non-ambulatory" (KW) OR wheelchair* (KW) OR "limited mobility" (KW) OR "immobil*"(KW) OR "mobility impairment" (KW) OR "neuromusculoskeletal disabilit*" (KW) OR amputee* (KW) OR "cerebral pals*" OR diplegi* (KW) OR quadripleg* (KW) OR tetrapleg* (KW) OR "muscular dystroph*" OR "spina bifida" (KW) OR "spinal dysraphism" (KW) OR "neural tube defect*" (KW) OR parapleg* (KW) paralys* (KW) OR "spinal cord injur*" OR handicapped (KW) OR "hereditary motor sensory neuropath*" (KW) OR "charcot-marie-tooth" (KW) OR "spinal muscular atroph*" (KW) OR Immobility (SH) OR Wheelchairs (SH) (exp) OR Amputees (SH) (exp) OR Cerebral palsy (SH) OR Muscular Dystrophy (SH) (exp) OR Spina Bifida (SH) OR Neural Tube Defects (SH) (exp) OR Paraplegia (SH) (exp) OR Spastic Paraplegia, Hereditary (SH) OR Spinal Cord Injuries (SH) (exp) OR Quadriplegia (SH) (exp) OR Neuropathies, Hereditary	sedentary (KW) OR "screen time" (KW) OR screentime (KW) OR "screen-time" (KW) OR "screen use" (KW) OR "screen exposure" (KW) OR "(television or TV) near/3 watch* OR "physical inactivity" (KW) OR "physically inactive" (KW) OR Life Style, Sedentary (SH) (exp) OR Television (SH) OR Screen Time (SH) OR Sitting (SH)	Publication date: 1996 – 2020 Language: English

For peer review only - http://bmjopen.bmj.com/site/about/guidelines.xhtml

	motor and sensory (exp) OR Charcot-Marie-Tooth Disease (SH) OR Muscular Atrophy, Spinal (SH) (exp)<br><br>AND<br><br>minors (KW) OR pediatric (KW) OR paediatrics (KW) OR pediatric (KW) OR paediatric (KW) OR child (KW) OR children (KW) OR baby (KW) OR childhood (KW) OR babies (KW) OR infant (KW) OR infants (KW) OR infancy (KW) OR school (KW) OR preschool (KW) OR adolescen* (KW) OR teen* (KW) OR youth (KW) OR "young adult*" (KW) OR "young person*" (KW) OR "young people" (KW) OR toddler* (KW) OR juvenile* (KW) OR Infant, Newborn (SH) (exp) OR Minors (Legal) (SH) (exp) OR Child (SH) (exp) OR Adolescence (SH) (exp)		
**Cochrane Library**<br><br>**Via Cochrane library website**<br><br>**Search date 30/11/2020**	Searched title and abstract only for:<br>“physical disability” OR “physical disabilities” OR “physically disabled” OR “non-ambulant” OR “non-ambulatory” OR wheelchair* OR “limited mobility” OR immobilit* OR “mobility impairment” OR “neuromusculoskeletal disabilit*” OR “neuromusculoskeletal disability” OR amputee* OR “cerebral palsy” OR diplegi* OR quadripleg* OR tetrapleg* OR “muscular dystrophy” OR “spina bifida” OR “spinal dysraphism” OR “neural tube defect” OR “neural tube defects” OR parapleg* OR paralys* OR “spinal cord injury” OR “spinal cord injuries” OR handicap* OR “hereditary motor sensory neuropathy” OR “hereditary motor sensory neuropathies” OR “charcot-marie-tooth” OR “spinal muscular atrophy” OR “spinal muscular atrophies” OR<br>Mobility Limitation (MeSH) (exp) OR Amputees (MeSH) (exp) OR<br>OR Wheelchairs (MeSH) (exp) OR<br>Cerebral Palsy (MeSH) (exp)<br>OR Muscular Dystrophies (MeSH) (exp)<br>OR Spinal Dysraphism (MeSH) (exp)<br>OR Neural Tube Defects (MeSH) (exp) OR Spinal Cord Injuries (MeSH) OR Paraplegia (MeSH) (exp) OR Quadriplegia (MeSH) (exp) OR Paralysis (MeSH) (exp) OR Spastic Paraplegia, Hereditary (MeSH) (exp) OR Hereditary Sensory and Motor Neuropathy (MeSH) (exp) OR Charvot-Marie-Tooth Disease (MeSH) (exp) OR Muscular Atrohy, Spinal (MeSH) (exp)<br><br>AND<br><br>minors OR pediatric OR paediatrics OR pediatrics OR paediatric OR child OR children OR baby OR childhood OR babies OR infant OR infants OR infancy OR school OR preschool OR adolescen* OR teen* OR youth OR "young	Searched title and abstract only for:<br>sedentary (KW) OR “screen time" (KW) OR screentime (KW) OR "screen-time" (KW) OR "screen use" (KW) OR "screen exposure" (KW) OR (television or TV) near/3 watch* OR "physical inactivity" (KW) OR "physically inactive" (KW) OR Sedentary Behavior (MeSH) (exp) OR Television (MeSH) (exp) OR Sitting position (MeSH) (exp) OR Screen Time (MeSH) (exp)	Cochrane Library publication date:<br>Jan 1996 and Sep 2020<br><br>Content type:<br>Cochrane reviews<br>Cochrane trials

For peer review only

	adult" OR “young adults” OR "young person" OR "young people" OR toddler* OR juvenile*) OR Young Adult (MeSH) OR Adolescent (MeSH) OR (MeSH) OR Infant (MeSH) OR Child (MeSH)		
**EMBASE: Excerpta Medica via Ovid 1974 to Nov 23rd 2020**<br><br>**Search date 25/11/2020**	“physical disabilit*” (KW) OR “physically disabled” (KW) OR “non-ambulant” (KW) OR “non-ambulatory” (KW) OR wheelchair* (KW) OR “limited mobility” (KW) OR “immobil*”(KW) OR “mobility impairment” (KW) OR “neuromusculoskeletal disability” (KW) (NB wouldn’t accept “neuromusculoskeletal disabilit*” OR amputee* (KW) OR “cerebral pals*” OR diplegi* (KW) OR quadripleg* (KW) OR tetrapleg* (KW) OR “muscular dystroph*” OR “spina bifida” (KW) OR “spinal dysraphism” (KW) OR “neural tube defect*” (KW) OR parapleg* (KW) paralys* (KW) OR “spinal cord injur*” OR handicap* (KW) OR “hereditary motor sensory neuropath*” (KW) OR “charcot-marie-tooth” (KW) OR "spinal muscular atroph*" (KW) OR Physical disability (SH) (exp) OR Wheelchair (SH) (exp) OR Limited Mobility (SH) (exp) OR Cerebral palsy (SH) (exp) (KW) OR Amputee (SH) (exp) OR Muscular dystrophy (SH) (exp) OR Duchenne Muscular Dystrophy (SH) (exp) OR Spina Bifida (SH) exp) OR Spinal Dysraphism (SH) (exp) OR Neural Tube Defect (SH) (exp) OR Paraplegia (SH) (exp) OR Paralysis (SH) (exp) OR Spastic paraplegia (SH) (exp) OR Quadriplegia (SH) (exp) OR Spinal cord injury (SH) (exp) OR Hereditary Motor Sensory Neuropathy (SH) (exp) OR Muscular Atrophy, Spinal (SH) (exp)<br><br>AND<br><br>minors (KW) OR pediatric (KW) OR paediatrics (KW) OR pediatric (KW) OR paediatric (KW) OR child (KW) OR children (KW) OR baby (KW) OR childhood (KW) OR babies (KW) OR infant (KW) OR infants (KW) OR infancy (KW) OR school (KW) OR preschool (KW) OR adolescen* (KW) OR teen* (KW) OR youth (KW) OR "young adult*" (KW) OR "young person*" (KW) OR "young people" (KW) OR toddler* (KW) OR juvenile* (KW) OR Young Adult (SH) (exp) OR Adolescent (SH) (exp) OR (SH) (exp) OR Infant (SH) (exp) OR Child (SH) (exp)	sedentary (KW) OR “screen time" (KW) OR screentime (KW) OR "screen-time" (KW) OR "screen use" (KW) OR "screen exposure" (KW) OR (television or TV) adj3 watch* OR "physical inactivity" (KW) OR "physically inactive" (KW) OR Sedentary lifestyle (SH) (exp) OR Sedentary time (SH) (exp) OR Screen time (SH) (exp) OR Television (SH) (exp) OR Television viewing (SH) (exp) OR Sitting (SH) (exp) OR Physical Inactivity (SH) (exp)	Publication year: 1996 – current English language
**MEDLINE (R) 1946 – November Week 2 2020 Via Ovid**<br><br>**Search date: 20/11/2020**	“physical disabilit*” (KW) OR “physically disabled” (KW) OR “non-ambulant” (KW) OR “non-ambulatory” (KW) OR wheelchair* (KW) OR “limited mobility” (KW) OR “immobil*”(KW) OR “mobility impairment” (KW) OR “neuromusculoskeletal disabilit*” (KW) OR amputee* (KW) OR “cerebral pals*” OR diplegi* (KW) OR quadripleg* (KW) OR tetrapleg* (KW) OR “muscular dystroph*” OR “spina bifida” (KW) OR “spinal dysraphism” (KW) OR “neural tube defect*” (KW) OR parapleg* (KW) paralys* (KW) OR “spinal cord injur*”	sedentary (KW) OR “screen time" (KW) OR screentime (KW) OR "screen-time" (KW) OR "screen use" (KW) OR "screen exposure" (KW) OR (television or TV) adj3 watch* OR "physical	Publication Year: 1996 – 2020 English language

For peer review only - http://bmjopen.bmj.com/site/about/guidelines.xhtml

	OR handicap* (KW) OR “hereditary motor sensory neuropath*” (KW) OR “charcot-marie-tooth” (KW) OR "spinal muscular atroph*" (KW) OR Wheelchairs (SH) (exp) OR Mobility Limitation (SH) (exp) OR Cerebral palsy (SH) (exp) (KW) OR Amputees (SH) (exp) OR Muscular dystrophies (SH) (exp) OR Spinal Dysraphism (SH) (exp) (KW) OR Neural Tube Defects (SH) (exp) OR Paraplegia (SH) (exp) OR Paralysis (SH) (exp) OR Spastic paraplegia, hereditary (SH) (exp) OR Quadriplegia (SH) (exp) OR Spinal cord injuries (SH) (exp) OR Hereditary Sensory and Motor Neuropathy (SH) (exp) OR Charcot-Marie-Tooth Disease (SH) (exp) OR Muscular Atrophy, Spinal (SH) (exp)<br><br>AND<br><br>minors (KW) OR pediatric (KW) OR paediatrics (KW) OR pediatric (KW) OR paediatric (KW) OR child (KW) OR children (KW) OR baby (KW) OR childhood (KW) OR babies (KW) OR infant (KW) OR infants (KW) OR infancy (KW) OR school (KW) OR preschool (KW) OR adolescen* (KW) OR teen* (KW) OR youth (KW) OR "young adult*" (KW) OR "young person*" (KW) OR "young people" (KW) OR toddler* (KW) OR juvenile* (KW) OR Minors (SH) (exp) OR Child (SH) (exp) OR Child, Preschool (SH) (exp) OR Infant, Newborn (SH) (exp) OR Infant (SH) (exp) OR Adolescent (SH) (exp) OR Young Adult (SH) (exp)	inactivity" (KW) OR "physically inactive" (KW) Sedentary Behavior (SH) (exp) OR Television (SH) (exp) OR Screen Time (SH) (exp) OR Sitting Position (SH) (exp) OR	
**Nursing and Allied Health Database Via ProQuest**<br><br>**Search date 20/11/2020**	“physical disabilit*” (KW) OR “physically disabled” (KW) OR “non-ambulant” (KW) OR “non-ambulatory” (KW) OR wheelchair* (KW) OR “limited mobility” (KW) OR “immobil*”(KW) OR “mobility impairment” (KW) OR “neuromusculoskeletal disabilit*” (KW) OR amputee* (KW) OR “cerebral pals*” OR diplegi* (KW) OR quadripleg* (KW) OR tetrapleg* (KW) OR “muscular dystroph*” OR “spina bifida” (KW) OR “spinal dysraphism” (KW) OR “neural tube defect*” (KW) OR parapleg* (KW) paralys* (KW) OR “spinal cord injur*” OR handicap* (KW) OR “hereditary motor sensory neuropath*” (KW) OR “charcot-marie-tooth” (KW) OR "spinal muscular atroph*" (KW) OR Neural Tube Defects (MeSH) OR Spastic Paraplegia, Hereditary (MeSH) OR Spina Bifida Occulta (MeSH) OR Amputees (MeSH) OR Quadriplegia (MeSH) OR Spina Bifida Cystica (MeSH) OR Charcot-Marie-Tooth Disease (MeSH) OR Mobility Limitation (MeSH) OR Paraplegia (MeSH) OR Muscular Dystrophies (MeSH) OR Spinal Cord Injuries (MeSH) OR Wheelchairs (MeSH) OR Muscular Atrophy, Spinal (MeSH) OR Spinal Dysraphism (MeSH) OR Hereditary Sensory & Motor Neuropathy (MeSH) OR Muscular	sedentary (KW) OR “screen time” (KW) OR screentime (KW) OR "screen-time" (KW) OR "screen use" (KW) OR "screen exposure" (KW) OR (television or TV) NEAR/3 watch* (KW) OR "physical inactivity" (KW) OR "physically inactive" (KW) OR sitting (KW) OR Sedentary lifestyle (MeSH) OR Television (MeSH)	Publication date: 01/01/1996 – 20/11/2020 English language only

For peer review only - http://bmjopen.bmj.com/site/about/guidelines.xhtml

	Dystrophy, Duchenne (MeSH) OR Paralysis (MeSH) OR Cerebral Palsy (MeSH)<br><br>AND<br><br>minors (KW) OR pediatric (KW) OR paediatrics (KW) OR pediatric (KW) OR paediatric (KW) OR child (KW) OR children (KW) OR baby (KW) OR childhood (KW) OR babies (KW) OR infant (KW) OR infants (KW) OR infancy (KW) OR school (KW) OR preschool (KW) OR adolescen* (KW) OR teen* (KW) OR youth (KW) OR "young adult*" (KW) OR "young person*" (KW) OR "young people" (KW) OR toddler* (KW) OR juvenile* (KW) OR Young Adult (MeSH) OR Adolescent (MeSH) OR (MeSH) OR Infant (MeSH) OR Child (MeSH)		
**Scopus**<br><br>**Search date 25/11/2020**	Title and abstract search only (not key words).<br>"physical disability*" (KW) OR "physically disabled" (KW) OR "non-ambulant" (KW) OR "non-ambulatory" (KW) OR wheelchair* (KW) OR "limited mobility" (KW) OR "immobil*"(KW) OR "mobility impairment" (KW) OR "neuromusculoskeletal disabilit*" (KW) OR amputee* (KW) OR "cerebral pals*" OR diplegi* (KW) OR quadripleg* (KW) OR tetrapleg* (KW) OR "muscular dystroph*" OR "spina bifida" (KW) OR "spinal dysraphism" (KW) OR "neural tube defect*" (KW) OR parapleg* (KW) paralys* (KW) OR "spinal cord injur*" OR handicap* (KW) OR "hereditary motor sensory neuropath*" (KW) OR "charcot-marie-tooth" (KW) OR "spinal muscular atroph*" (KW)<br><br>AND<br><br>minors (KW) OR pediatric (KW) OR paediatrics (KW) OR pediatric (KW) OR paediatric (KW) OR child (KW) OR children (KW) OR baby (KW) OR childhood (KW) OR babies (KW) OR infant (KW) OR infants (KW) OR infancy (KW) OR school (KW) OR preschool (KW) OR adolescen* (KW) OR teen* (KW) OR youth (KW) OR "young adult*" (KW) OR "young person*" (KW) OR "young people" (KW) OR toddler* (KW) OR juvenile* (KW)	Title and abstract search only (not key words).<br>sedentary (KW) OR "screen time" (KW) OR screentime (KW) OR "screen-time" (KW) OR "screen use" (KW) OR "screen exposure" (KW) OR (television or TV) NEAR/3 watch* OR "physical inactivity" (KW) OR "physically inactive" (KW) OR sitting	Publication year: 1996-2020 English language
**Web of Science Core Collection**<br><br>**Search date 02/12/2020**	Searched Title and Abstract for:<br>"physical disabilit*" OR "physically disabled" OR "non-ambulant" OR "non-ambulatory" OR wheelchair* OR "limited mobility" OR immobil* OR "mobility impairment" OR "neuromusculoskeletal disabilit*" OR amputee* OR "cerebral pals*" OR diplegi* OR quadripleg* OR tetrapleg* OR "muscular dystroph*" OR "spina bifida" OR "spinal dysraphism" OR "neural tube defect*" OR parapleg* OR paralys* OR "spinal cord injur*" OR handicap* OR "hereditary motor sensory	Searched Title and Abstract for:<br>sedentary OR "screen time" OR screentime OR "screen-time" OR "screen use" OR "screen exposure" OR (television or TV) NEAR/3 watch* OR	Custom year range 1996-2020 English language

For peer review only

For peer review only - http://bmjopen.bmj.com/site/about/guidelines.xhtml

	neuropath*” OR “charcot-marie-tooth” OR “spinal muscular atroph*”<br><br>AND<br><br>minors OR pediatric OR paediatrics OR pediatrics OR paediatric OR child OR children OR baby OR childhood OR babies OR infant OR infants OR infancy OR school OR preschool OR adolescen* OR teen* OR youth OR "young adult*" OR "young person*" OR "young people" OR toddler* OR juvenile*	"physical inactivity" OR "physically inactive" OR sitting	

For peer review only

PRISMA-P (Preferred Reporting Items for Systematic review and Meta-Analysis Protocols) 2015 checklist: recommended items to address in a systematic review protocol*

Section and topic	Item No	Checklist item	Page Number
**ADMINISTRATIVE INFORMATION**			
Title:			
Identification	1a	Identify the report as a protocol of a systematic review	1
Update	1b	If the protocol is for an update of a previous systematic review, identify as such	
Registration	2	If registered, provide the name of the registry (such as PROSPERO) and registration number	11
Authors:			
Contact	3a	Provide name, institutional affiliation, e-mail address of all protocol authors; provide physical mailing address of corresponding author	1
Contributions	3b	Describe contributions of protocol authors and identify the guarantor of the review	
Amendments	4	If the protocol represents an amendment of a previously completed or published protocol, identify as such and list changes; otherwise, state plan for documenting important protocol amendments	NA
Support:			
Sources	5a	Indicate sources of financial or other support for the review	
Sponsor	5b	Provide name for the review funder and/or sponsor	
Role of sponsor or funder	5c	Describe roles of funder(s), sponsor(s), and/or institution(s), if any, in developing the protocol	12
**INTRODUCTION**			
Rationale	6	Describe the rationale for the review in the context of what is already known	3-4
Objectives	7	Provide an explicit statement of the question(s) the review will address with reference to participants, interventions, comparators, and outcomes (PICO)	4-6 using PCC rather than PICO
**METHODS**			
Eligibility criteria	8	Specify the study characteristics (such as PICO, study design, setting, time frame) and report characteristics (such as years considered, language, publication status) to be used as criteria for eligibility for the review	5-6
Information sources	9	Describe all intended information sources (such as electronic databases, contact with study authors, trial registers or other grey literature sources) with planned dates of coverage	4-6

For peer review only

Search strategy	10	Present draft of search strategy to be used for at least one electronic database, including planned limits, such that it could be repeated	6-8 and supplementary file
Study records:			
Data management	11a	Describe the mechanism(s) that will be used to manage records and data throughout the review	8
Selection process	11b	State the process that will be used for selecting studies (such as two independent reviewers) through each phase of the review (that is, screening, eligibility and inclusion in meta-analysis)	8-9
Data collection process	11c	Describe planned method of extracting data from reports (such as piloting forms, done independently, in duplicate), any processes for obtaining and confirming data from investigators	9-10
Data items	12	List and define all variables for which data will be sought (such as PICO items, funding sources), any pre-planned data assumptions and simplifications	9-10
Outcomes and prioritization	13	List and define all outcomes for which data will be sought, including prioritization of main and additional outcomes, with rationale	9-10
Risk of bias in individual studies	14	Describe anticipated methods for assessing risk of bias of individual studies, including whether this will be done at the outcome or study level, or both; state how this information will be used in data synthesis	8-9
Data synthesis	15a	Describe criteria under which study data will be quantitatively synthesised	10
	15b	If data are appropriate for quantitative synthesis, describe planned summary measures, methods of handling data and methods of combining data from studies, including any planned exploration of consistency (such as I^2, Kendall's τ)	Potential to be assessed as part of data analysis
	15c	Describe any proposed additional analyses (such as sensitivity or subgroup analyses, meta-regression)	NA
	15d	If quantitative synthesis is not appropriate, describe the type of summary planned	Appropriateness to be assessed as part of analysis
Meta-bias(es)	16	Specify any planned assessment of meta-bias(es) (such as publication bias across studies, selective reporting within studies)	NA
Confidence in cumulative evidence	17	Describe how the strength of the body of evidence will be assessed (such as GRADE)	8-10

*** It is strongly recommended that this checklist be read in conjunction with the PRISMA-P Explanation and Elaboration (cite when available) for important clarification on the items. Amendments to a review protocol should be tracked and dated. The copyright for PRISMA-P (including checklist) is held by the PRISMA-P Group and is distributed under a Creative Commons Attribution Licence 4.0.**

From: Shamseer L, Moher D, Clarke M, Ghersi D, Liberati A, Petticrew M, Shekelle P, Stewart L, PRISMA-P Group. Preferred reporting items for systematic review and meta-analysis protocols (PRISMA-P) 2015: elaboration and explanation. BMJ. 2015 Jan 2;349(jan02 1):g7647.

For peer review only

BMJ Open

Sedentary Behaviour in Non-Ambulant Children and Young People with Physical Disabilities: A Systematic Search and Review Protocol

Journal:	*BMJ Open*
Manuscript ID	bmjopen-2021-053077.R1
Article Type:	Protocol
Date Submitted by the Author:	17-Sep-2021
Complete List of Authors:	Bradbury, Marilyn; University of Birmingham, School of Sport, Exercise and Rehabilitation Sciences; Birmingham Community Healthcare NHS Foundation Trust, Research and Innovation<br>O'Brien, Ciara; University of Surrey, School of Psychology<br>Giles, Nathan<br>Fenton, Sally; University of Birmingham, School of Sport, Exercise and Rehabilitation Sciences<br>Neilson, Sue; University of Birmingham, School of Nursing<br>Duda, Joan; University of Birmingham, School of Sport, Exercise & Rehabilitation Sciences
**Primary Subject Heading**:	Rehabilitation medicine
Secondary Subject Heading:	Sports and exercise medicine
Keywords:	Developmental neurology & neurodisability < PAEDIATRICS, REHABILITATION MEDICINE, SOCIAL MEDICINE

SCHOLARONE™
Manuscripts

I, the Submitting Author has the right to grant and does grant on behalf of all authors of the Work (as defined in the below author licence), an exclusive licence and/or a non-exclusive licence for contributions from authors who are: i) UK Crown employees; ii) where BMJ has agreed a CC-BY licence shall apply, and/or iii) in accordance with the terms applicable for US Federal Government officers or employees acting as part of their official duties; on a worldwide, perpetual, irrevocable, royalty-free basis to BMJ Publishing Group Ltd ("BMJ") its licensees and where the relevant Journal is co-owned by BMJ to the co-owners of the Journal, to publish the Work in this journal and any other BMJ products and to exploit all rights, as set out in our licence.

The Submitting Author accepts and understands that any supply made under these terms is made by BMJ to the Submitting Author unless you are acting as an employee on behalf of your employer or a postgraduate student of an affiliated institution which is paying any applicable article publishing charge ("APC") for Open Access articles. Where the Submitting Author wishes to make the Work available on an Open Access basis (and intends to pay the relevant APC), the terms of reuse of such Open Access shall be governed by a Creative Commons licence – details of these licences and which Creative Commons licence will apply to this Work are set out in our licence referred to above.

Other than as permitted in any relevant BMJ Author's Self Archiving Policies, I confirm this Work has not been accepted for publication elsewhere, is not being considered for publication elsewhere and does not duplicate material already published. I confirm all authors consent to publication of this Work and authorise the granting of this licence.

For peer review only

TITLE: SEDENTARY BEHAVIOUR IN NON-AMBULANT CHILDREN AND YOUNG PEOPLE WITH PHYSICAL DISABILITIES: A SYSTEMATIC SEARCH AND REVIEW PROTOCOL

AUTHOR INFORMATION

Corresponding author: Marilyn Bradbury

Affiliations: School of Sport, Exercise and Rehabilitation Sciences, University of Birmingham, Birmingham Community Healthcare NHS Foundation Trust.

Postal address: Research and Innovation, Birmingham Community Healthcare NHS Foundation Trust, Trust Headquarters, 3 Priestley Wharf, 20 Holt Street, Birmingham, B7 4BN, United Kingdom

email: marilyn.bradbury1@nhs.net

ORCID ID: 0000-0001-6000-7229

Ciara O'Brien, School of Psychology, University of Surrey, Surrey, UK. ciara.o'brien@surrey.ac.uk

ORCID ID: 0000-0002-2215-6001

Nathan Giles, Lay member of the DoMore Study Steering Group. Birmingham, UK. NathanGiles20@outlook.com,

Sally A.M. Fenton, School of Sport, Exercise and Rehabilitation Sciences, University of Birmingham, Birmingham, UK S.A.M.Fenton@bham.ac.uk

Sue Neilson, School of Nursing, University of Birmingham, Birmingham, UK. S.J.Neilson@bham.ac.uk

Joan L. Duda, School of Sport, Exercise and Rehabilitation Sciences, University of Birmingham, Birmingham, UK J.L.DUDA@bham.ac.uk

Word count: 2, 576

For peer review only - http://bmjopen.bmj.com/site/about/guidelines.xhtml

ABSTRACT

Introduction:

Non-ambulant children and young people with physical disabilities are at high risk of experiencing negative health outcomes associated with sedentary time. A previous scoping review summarising evidence relating to sedentary behaviours of children with physical disabilities identified the need for validated methods of measuring physical activity of children who use wheelchairs and evaluation of interventions to reduce sedentary time. The scoping review did not assess the quality of evidence relating to this topic, therefore its validity remains unclear. No reviews focussing on non-ambulant children and young people up to the age of 25 years have been undertaken.

The objectives of this systematic search and review are to:

- Identify all peer reviewed articles relating to sedentary behaviour of non-ambulant children and young people.
- Categorise the articles according to study design and 4 sub questions relating to 1) measurement 2) patterns 3) associated risks and 4) interventions to reduce sedentary time or behaviour.
- Critically appraise quality of the articles using established critical appraisal tools.
- Summarise the evidence for each sub question. Describe its cumulative strength and identify knowledge gaps.

Methods and Analysis:

This protocol was developed using the PRISMA-P checklist. The research questions, inclusion / exclusion criteria and search terms have been developed a priori using the "Population, Concept and Context" framework. Online databases will be systematically searched to identify peer reviewed articles published between 1996 and 2021. Two reviewers will screen, categorise and critically appraise the articles. Data extraction and analysis will be verified by the second reviewer.

Results will be reported as a best evidence synthesis, with reference to the PRISMA checklist.

Ethics and Dissemination:

Ethical approval is not required. The review will be submitted to an appropriate peer reviewed journal.

Registration

The review is registered on the Open Science Framework database.

KEYWORDS

Sedentary behaviour, physical disability, children, young people, wheelchair, non-ambulant, systematic search and review.

ARTICLE SUMMARY

STRENGTHS AND LIMITATIONS OF THIS STUDY

For peer review only - http://bmjopen.bmj.com/site/about/guidelines.xhtml

- This will be the first review that systematically identifies evidence relating to sedentary behaviours of non-ambulant children and young people using a robust and comprehensive search strategy.
- This will be the first review relating to sedentary behaviour of children and young people with disabilities that includes an assessment of the quality of the included research.
- A wide age range is incorporated, allowing consideration of how sedentary behaviour changes on the journey from childhood into adulthood.
- A limitation is that only peer-reviewed literature will be included. The objective is to summarise the findings of high quality research and not to comprehensively identify all literature relating to the topic.
- A limitation is that only studies which are written in English will be included.

INTRODUCTION

Sedentary behaviour is defined as "any waking behaviour characterized by an energy expenditure ≤1.5 metabolic equivalents (METs) while in a sitting or reclining posture",[1]. A large body of evidence from a variety of study designs suggests that reducing sedentary time is associated with lower health risk in children and young people aged 5-17 years,[2]. This body of evidence includes studies measuring both sedentary time and specific behaviours such as television watching. In children, time spent watching television has been associated with increased body mass index, reduced physical fitness, low self-esteem and reduced academic achievement,[2].

Children with disabilities are more sedentary than their able bodied peers,[3]. Lower levels of gross motor function,[4-6] and increasing age,[6-8] , are linked to increased sedentary behaviours in children with disabilities. Young people who are non-ambulant (i.e., unable to walk, or can walk short distances using a body support walker ["therapeutic walking"]), are therefore at greatest risk of experiencing negative health outcomes associated with sedentary time. Young people in this group may have conditions such as (but not limited to) cerebral palsy, spina bifida, muscular dystrophy, irreversible traumatic injuries or rare conditions.

A scoping review of sedentary behaviour and children with physical disabilities has recently summarised research relating to measurement of sedentary time, sedentary behaviour patterns, and interventions to reduce or break up time spent sedentary,[3]. The review identified that there is a lack of research evaluating interventions to decrease the sedentary behaviours of children with physical disabilities. In addition, the need for research that focuses on improving our understanding of sedentary behaviour patterns in children who are non-ambulant, including the validity of using accelerometers to measure sedentary time in this group, was recognised. To date, no reviews incorporating a quality assessment of research relating to this topic or focussing specifically on non-ambulant children and young people have been undertaken. This review builds on that of Ganz et al., (2020),[3] by focusing specifically on non-ambulant children and young people, incorporating critical evaluation of the quality of the evidence, considering risks associated with sedentary time or behaviour for this population and searching a wider range of databases. Contemporary work that has been published since the searches for the scoping review were undertaken will also be captured. The present review is intended to inform the development of an intervention which aims to reduce sedentary behaviour in non-ambulant young people with long-term disabilities. The targeted age

For peer review only - http://bmjopen.bmj.com/site/about/guidelines.xhtml

range for the planned intervention includes young people aged 12 - 25 years. The present review will include research relating to a wide age range, from birth to 25 years. This extends the age range of the recent scoping review to include 18-25 year olds and summarises the evidence throughout children's development. Covering this wide age range provides a broad perspective that allows an in depth understanding of children's behaviour, taking into consideration how sedentary behaviour changes throughout the journey from childhood into adulthood. This captures key transition points that may have an impact on sedentary behaviour such as leaving school and moving from children's to adult's health services.

The objectives of the review are to:

1) Identify all peer reviewed journal articles relating to measurement of sedentary time, patterns of sedentary behaviour and interventions to decrease sedentary time or behaviours for non-ambulant children and young people.
2) Categorise the articles according to study design and 4 sub questions relating to 1) measurement 2) patterns 3) associated risks and 4) interventions to reduce sedentary time or behaviour for non-ambulant children and young people.
3) Critically appraise the quality of the articles using appropriate tools to evaluate the study design.
4) Produce a best evidence synthesis,[9, 10] which summarises the evidence for each sub question and describes its cumulative strength, based on the hierarchy of evidence and the quality ratings assigned following critical appraisal. Identify knowledge gaps for this specific population.

The overarching research question is "What is the quality of the evidence relating to sedentary behaviour in non-ambulant children and young people?". The sub-questions are:

1) Do validated measures of sedentary time or behaviour of non-ambulant children and young people exist?
2) Have patterns of sedentary time or behaviour of non-ambulant children and young people been described?
3) Have risks associated with sedentary time or behaviour been explored or quantified for non-ambulant children and young people?
4) What evidence-based interventions to reduce sedentary behaviour or decrease and break up sedentary time in this population have been evaluated and what is the strength of this evidence?

Grant and Booth,[10] have described the typology of the systematic search and review. They highlight how this type of review combines a systematic search method with a critical review analysis. This review typology is used to answer broad questions and often incorporates multiple study designs. The analysis results in a best evidence synthesis to describe what is known, recommendations for practice and limitations of the evidence. A systematic search and review typology is being adopted for this review because the topic is too broad to undertake a traditional systematic review, which should focus on only one specific question. The incorporation of several

For peer review only - http://bmjopen.bmj.com/site/about/guidelines.xhtml

For peer review only

sub-questions and the inclusion of studies from multiple paradigms with widely varying study designs makes a systematic review methodology inappropriate,[10].

METHODS AND ANALYSIS

Search Strategy

Databases searched by Ganz et al., (2020),[3] were CINAHL, MEDLINE, ERIC, EMBASE and SPORTDiscus. The electronic databases searched in this review include the following additional databases, on a variety of platforms to expand the breadth of the search: The Allied and Complementary Medicine Database (AMED)**,** APA PsycINFO**,** Applied Social Sciences Index & Abstracts (ASSIA), Child development and adolescent studies, CINAHL Plus, Cochrane Library**,** EMBASE: Excerpta, ERIC, MEDLINE (R), Nursing and Allied Health Database**,** Scopus, SPORTDiscus and Web of Science.

The searches for this review will be limited to publication dates between January 1996 – September 2021, as the majority of the relevant publications identified by Ganz et al.,[3] were published after 2013 (72%), with the earliest included paper being published in 1996. Searches were undertaken by Ganz et al.,[3] in November 2018, so new papers published between November 2018 and September 2021 will be screened in this review. Reference lists of eligible articles will also be screened for any relevant articles that may have been missed in the database searches.

Traditional tools used to produce search strategies in systematic reviews include the PICO tool (which is ideally suited to quantitative studies) and the SPIDER tool (which is suitable for qualitative and mixed methods research),[11]. In this review, quantitative, qualitative, mixed methods studies and reviews are all relevant. Therefore, the “Population, Concept and Context” (PCC) framework recommended for use in scoping reviews, has been adopted,[12]. The PCC framework is more appropriate than the PICO tool to capture the wide range of study designs associated with multiple sub-questions. The inclusion and exclusion criteria developed using the PCC framework is outlined in Table 1 below.

For peer review only - http://bmjopen.bmj.com/site/about/guidelines.xhtml

Table 1 Inclusion and exclusion criteria. PCC = Population, Concept, Context. GMFCS = Gross Motor Function Classification System.

PCC framework element	Inclusion	Exclusion
**Population**	Participants with long term physical disabilities.<br>AND<br>A proportion of the participants are non-ambulant. The proportion of non-ambulant participants is clear.<br>For participants with cerebral palsy, children and young people in Gross Motor Function Classification System (GMFCS) levels IV-V are considered non-ambulant.<br>AND<br>Article includes participants under 25 years.	Participants with intellectual disabilities, visual or hearing impairments and no physical disability.<br>OR<br>Participants with a temporary lack of ambulation due to injury or short-term illness.<br>OR<br>The article includes ambulant participants only, or the proportion of non-ambulant participants is unclear.<br>For participants with cerebral palsy, children and young people in GMFCS levels I-III are considered ambulant.<br>OR<br>All participants in the article are 25 and over
**Concept**	Article explores measurement of, patterns of, or risks related to sedentary behaviour.<br>OR<br>Article evaluates feasibility or effectiveness of an intervention to decrease or break up sedentary time.<br>NB any outcome measured in studies matching the inclusion criteria will be accepted.	Papers relating to physical activity that have not measured or explored patterns or correlates of sedentary time or behaviour specifically.
**Context**	Peer reviewed journal articles (quantitative, qualitative, mixed methods), scoping reviews, systematic reviews, theses / dissertations<br>AND<br>Published in the English language.<br>AND<br>Published between January 1996 and September 2021.	Grey literature, conference abstracts, conference proceedings, conference papers, conference review, protocols, commentaries, opinion articles, literature reviews (not systematic), narrative reviews, books or chapters.<br>OR<br>Published in languages other than English.<br>OR

view only

For peer review only - http://bmjopen.bmj.com/site/about/guidelines.xhtml

		Published before 1996 or after September 2021.

As quality rather than scope of the evidence is the focus of this review, grey literature has been excluded. The search strategy and examples of the search terms used in two of the databases (one which offers subject heading searches and a second which does not) are shown in Table 2. Please refer to the supplementary material (File 1) for the full search strategy. Development of the search strategy terms was guided by those adopted by Ganz et al., (2020),[3], however, additional terms have been included to ensure the search is specific to the overarching research question and the sub-questions outlined above. Iterative cycles of trying different terms were carried out in order to decide upon the terms that yielded the most appropriate results and to ensure the terms were suitable across the wide range of databases.

Table 2. Search strategy using PCC framework. exp = exploded; SH = subject heading; KW = keyword in title or abstract. NB where subject headings were not exploded, this is because this option was not available.

Database	Population	Concept	Limits applied
**AMED - The Allied and Complementary Medicine Database**<br><br>**Via EBSCOhost**	“physical disabilit*” (KW) OR “physically disabled” (KW) OR “non-ambulant” (KW) OR “non-ambulatory” (KW) OR wheelchair* (KW) OR “limited mobility” (KW) OR “immobil*”(KW) OR “mobility impairment” (KW) OR “neuromusculoskeletal disabilit*” (KW) OR amputee* (KW) OR “cerebral pals*” OR diplegi* (KW) OR quadripleg* (KW) OR tetrapleg* (KW) OR “muscular dystroph*” OR “spina bifida” (KW) OR “spinal dysraphism” (KW) OR “neural tube defect*” (KW) OR parapleg* (KW) paralys* (KW) OR “spinal cord injur*” OR handicap* (KW) OR “hereditary motor sensory neuropath*” (KW) OR “charcot-marie-tooth” (KW) OR "spinal muscular atroph*" (KW)<br><br>AND<br><br>minors (KW) OR pediatric (KW) OR paediatrics (KW) OR pediatric (KW) OR paediatric (KW) OR child (KW) OR children (KW) OR baby (KW) OR childhood (KW) OR babies (KW) OR infant (KW) OR infants (KW) OR infancy	sedentary (KW) OR “screen time" (KW) OR screentime (KW) OR "screen-time" (KW) OR "screen use" (KW) OR "screen exposure" (KW) OR (television or TV) NEAR/3 watch* OR "physical inactivity" (KW) OR "physically inactive" (KW) OR sitting (KW)	Published date: 1996 – 2021<br>Language: English

	(KW) OR school (KW) OR preschool (KW) OR adolescen* (KW) OR teen* (KW) OR youth (KW) OR "young adult*" (KW) OR "young person*" (KW) OR "young people" (KW) OR toddler* (KW) OR juvenile* (KW)		
**EMBASE: Excerpta Medica via Ovid**	"physical disabilit*" (KW) OR "physically disabled" (KW) OR "non-ambulant" (KW) OR "non-ambulatory" (KW) OR wheelchair* (KW) OR "limited mobility" (KW) OR "immobil*"(KW) OR "mobility impairment" (KW) OR "neuromusculoskeletal disability" (KW) (NB wouldn't accept "neuromusculoskeletal disabilit*" OR amputee* (KW) OR "cerebral pals*" OR diplegi* (KW) OR quadripleg* (KW) OR tetrapleg* (KW) OR "muscular dystroph*" OR "spina bifida" (KW) OR "spinal dysraphism" (KW) OR "neural tube defect*" (KW) OR parapleg* (KW) paralys* (KW) OR "spinal cord injur*" OR handicap* (KW) OR "hereditary motor sensory neuropath*" (KW) OR "charcot-marie-tooth" (KW) OR "spinal muscular atroph*" (KW) OR Physical disability (SH) (exp) OR Wheelchair (SH) (exp) OR Limited Mobility (SH) (exp) OR Cerebral palsy (SH) (exp) (KW) OR Amputee (SH) (exp) OR Muscular dystrophy (SH) (exp) OR Duchenne Muscular Dystrophy (SH) (exp) OR Spina Bifida (SH) exp) OR Spinal Dysraphism (SH) (exp) OR Neural Tube Defect (SH) (exp) OR Paraplegia (SH) (exp) OR Paralysis (SH) (exp) OR Spastic paraplegia (SH) (exp) OR Quadriplegia (SH) (exp) OR Spinal cord injury (SH) (exp) OR Hereditary Motor Sensory Neuropathy (SH) (exp) OR Muscular Atrophy, Spinal (SH) (exp)<br><br>AND<br><br>minors (KW) OR pediatric (KW) OR paediatrics (KW) OR pediatric (KW) OR paediatric (KW) OR child (KW) OR children (KW) OR baby (KW) OR childhood (KW) OR babies (KW) OR infant (KW) OR infants (KW) OR infancy (KW) OR school (KW) OR preschool (KW) OR adolescen* (KW) OR teen* (KW) OR youth (KW) OR "young adult*" (KW) OR "young person*" (KW) OR "young people" (KW) OR toddler* (KW) OR juvenile* (KW) OR Young Adult (SH) (exp) OR Adolescent (SH) (exp) OR (SH) (exp) OR Infant (SH) (exp) OR Child (SH) (exp)	sedentary (KW) OR "screen time" (KW) OR screentime (KW) OR "screen-time" (KW) OR "screen use" (KW) OR "screen exposure" (KW) OR (television or TV) adj3 watch* OR "physical inactivity" (KW) OR "physically inactive" (KW) OR Sedentary lifestyle (SH) (exp) OR Sedentary time (SH) (exp) OR Screen time (SH) (exp) OR Television (SH) (exp) OR Television viewing (SH) (exp) OR Sitting (SH) (exp) OR Physical Inactivity (SH) (exp)	Publication year: 1996 – current English language

Searches for each group of terms defining the "Population" and the "Concept" (Table 1) will be conducted on separate lines in the databases, with individual terms combined as shown in Table 2.

For peer review only - http://bmjopen.bmj.com/site/about/guidelines.xhtml

The results from these searches will then be combined using AND. The limits outlined in Table 2 were applied to the search results prior to exporting citations to Endnote reference management software,[13].

Final searches will be conducted in September 2021.Our planned timelines for the study are as follows: screening of papers for inclusion will be carried out between September and November 2021, appraisal of included articles and data extraction between December and April 2022 and production of the best evidence synthesis and writing of the review article between May and July 2022.

Management of Study Records

All citations from the individual databases searched will be exported into a bibliographic management software program,[13]. Records from the individual database searches will be combined and duplicates removed. The titles and abstracts will be screened for relevance by two independent reviewers using the inclusion and exclusion criteria outlined in Table 1. Articles will be rated as relevant, not relevant, or not known. Articles will be rated as not known if there is insufficient information in the abstract to decide its relevance, or if the reviewer is uncertain of whether or not it fits the inclusion criteria. Full text articles will be obtained for all articles deemed to be “relevant” or “not known” following screening. Any discrepancies between the reviewers in relation to the inclusion of articles will be discussed following review of the full text and a consensus agreed. A third reviewer will be available to review full text articles for which agreement cannot be reached between the first and second reviewers.

Assessing quality of the evidence.

The internal validity of each article will be assessed using an appropriate tool. Quality and risk of bias will, therefore be assessed at study, but not outcome level. Full text articles will be evaluated by two independent reviewers. An overall rating of the quality of each article (good, fair, poor or unclear) will be assigned by both reviewers, based on a risk of bias assessment using a collection of critical appraisal tools. If there are disagreements between the two reviewers that cannot be reconciled through discussion, the third reviewer will be consulted. Any articles rated as “unclear” by either reviewer will also be discussed by the two independent reviewers and if required referred to the third reviewer to rate the quality of the article as good, fair or poor.

The Cochrane handbook explains that studies at high risk of bias should be given reduced weight in comparison to studies at low risk of bias,[14]. The flaws that exist in studies with a high risk of bias cannot be disregarded in the best evidence synthesis we will produce. It is important to find a balance between bias and precision. Including all studies in the review, regardless of their quality offers a high level of precision but serious risk of bias. Only including high quality studies with low risk of bias reduces the precision of the evidence synthesis. The Cochrane Handbook suggests four strategies authors can select from to present their results meaningfully,[14]. These strategies relate to systematic reviews of interventions and therefore focus on management of quantitative data from randomised control trials in meta-analysis. The scoping review previously undertaken gives an indication of the designs of studies that are likely to be included in this review,[3]. Thirteen articles in the existing review included non-ambulant participants. Their designs were described as: cross-sectional or cross-sectional survey, longitudinal, pre-post test, prospective cohort and

For peer review only - http://bmjopen.bmj.com/site/about/guidelines.xhtml

validation studies. As they are not randomised control trials of interventions, the strategies recommended in the Cochrane handbook are therefore difficult to apply in this review. The strategy we will adopt is as follows; to minimise serious risk of bias, articles rated as being poor quality will be excluded from this review. Articles rated as good or fair quality will be accepted for data extraction and inclusion in the evidence synthesis but their quality rating will be taken into account.

Reviewers will independently categorise each article by sub question and design.

The work of Ganz et al.,[3] gives an indication of the types of critical appraisal tools that will be required to assess risk of bias. The following critical appraisal tools will therefore be used to evaluate the quality of these studies:

The Quality Assessment Tool for Observational Cohort and Cross-Sectional Studies,[15]

The Quality Assessment Tool for Before-After (Pre-Post) Studies With No Control Group,[15]

If additional systematic reviews, randomised control trials, mixed methods studies or qualitative studies are identified, the following tools will be used to assess quality and risk of bias:

The CASP Systematic Review Checklist,[16]

The Cochrane Collaboration's tool for assessing risk of bias in randomised trials,[17]

The Mixed Methods Appraisal Tool,[18]

The CASP Qualitative Checklist,[16]

Data Extraction

One reviewer will undertake data extraction for included articles. The second reviewer will check the accuracy of this data extraction. Table 3 shows the data items which will be extracted from each article. If the data items listed below are not included in the article, the corresponding author will be contacted twice to request this information. If no response is received, it will be marked as unavailable.

For peer review only

Table 3. Data extraction items.

Data Item								
Author (year) (ref)<br><br>Country of origin?	Participants*	Objectives	Design	Intervention (Sub-quesiton 3 studies only)	Methods**	Results (specific to sedentary behaviour)	Critical appraisal tool	Quality of article (good, fair, poor)
*Participant data will include total number of participants, number of non-ambulant participants, diagnosis, age range, mean age and standard deviation. GMFCS level / level of impairment of participants if available.<br>**Methods will include type of measure or data collection method (e.g. gas analysis, accelerometer make and model, patient report (survey, interview, validated patient reported outcome measure), protocol (e.g. placement of accelerometer, accelerometer cut point used, wear protocol), data reduction if applicable (e.g. cut-points), outcome variable used in study if applicable (e.g. Sedentary time, sedentary behaviour (total or min/day)								

Results

The search results will be presented in a PRISMA flowchart. The flowchart will show the number of articles identified, the number of duplications and articles included and excluded at each stage, the reasons for exclusions and number of articles excluded for each reason. Four tables will be produced to summarise the evidence from included articles, one for each of the sub questions, containing the data items described in Table 3.

Public and Patient Involvement

Three public and patient involvement representatives have reviewed this protocol and provided feedback. They will also follow the progress of the review, having oversight of and providing feedback on our processes of screening the papers, assessing quality of relevant articles and disseminating the results. One of these three representatives has also kindly agreed to act as the third reviewer.

Best evidence synthesis

Slavin,[9] describes how producing a best evidence synthesis allows reviewers propose conclusions or hypotheses about what we can learn from a body of evidence in relation to a particular topic in situations where there are studies carried out by a variety of research teams, involving different populations and methods to examine the same phenomenon. There may also be a lack of consensus in the conclusions of these studies. The best evidence synthesis consists of a critical examination of

For peer review only - http://bmjopen.bmj.com/site/about/guidelines.xhtml

evidence such as this, producing justified conclusions and transparently discussing any evidence that contradicts these conclusions.

In this review, the strength of the cumulative evidence for each sub question will be described by summarising what is known, for which age group and diagnoses, and with what level of confidence (considering the hierarchy of evidence and quality rating). Diagnosis, age of onset and cognitive abilities of the children included in the research is likely to vary widely and will be an important consideration when summarising what is known. An assessment of the heterogeneity of the evidence and whether there is any potential for evidence synthesis will be undertaken and described. Cultural and economic differences between countries will be an important consideration in relation to the external validity or transferability of findings from individual studies. Any knowledge gaps will be identified and potential directions for future research will be discussed for each sub question.

ETHICS AND DISSEMINATION

Ethical approval is not required to undertake this review. The review will be submitted for peer review in an appropriate journal.

REGISTRATION

The review has been registered on the Open Science Framework database. DOI: https://doi.org/10.17605/OSF.IO/SQXJB. Any protocol amendments will be recorded in the Open Science Framework database.

ACKNOWLEDGMENTS

The authors would like to thank public and patient involvement representatives Roger Leek and Jacqueline Hawkins, and librarians Lynne Harris and Abimbola Alayo for their contributions.

CONTRIBUTORSHIP STATEMENT

Authors and contributors listed in the acknowledgements made the following contributions:
Marilyn Bradbury: Conceptualisation, methodology, writing – original draft, writing - review and editing, visualisation, project administration, funding acquisition.
Dr Ciara O’Brien: Methodology, writing - review and editing, project administration.
Nathan Giles: Writing – review and editing, project administration.
Dr. Sally Fenton: Conceptualisation, methodology, writing - review and editing, visualisation.
Dr. Sue Neilson: Conceptualisation, methodology, writing - review and editing, visualisation.
Prof Joan Duda: Conceptualisation, methodology, writing - review and editing, visualisation.
Public and patient involvement representatives, Roger Leek and Jacqueline Hawkins: Writing – review and editing.

COMPETING INTERESTS STATEMENT

All authors have completed the ICMJE uniform disclosure form at www.icmje.org/coi_disclosure.pdf and declare: Marilyn Bradbury had financial support from the National Institute of Health Research for the submitted work; no financial relationships with any organisations that might have an interest in the submitted work in the previous three years; no other relationships or activities that could appear to have influenced the submitted work.

For peer review only - http://bmjopen.bmj.com/site/about/guidelines.xhtml

FUNDING

This work was supported by the National Institute for Health Research (NIHR), grant number ICA-CDRF-2018-04-ST2-021. The funder has not been involved in the development of this protocol.

REFERENCES

1. Sedentary Behaviour Research Network. Letter to the Editor: Standardized use of the terms "sedentary" and "sedentary behaviours". *Appl Physiol Nutr Metab* Online [eLetter]. 27 April 2012. https://doi.org/10.1139/h2012-024.
2. Tremblay MS, LeBlanc AG, Kho ME, et al. Systematic review of sedentary behaviour and health indicators in school-aged children and youth. *Int J Behav Nutr Phys Act*. 2011;8(1):98.
3. Ganz F, Hammam N, Pritchard L. Sedentary behavior and children with physical disabilities: a scoping review. *Disabil Rehabil*. 2020:1-13.
4. Oftedal S, Bell KL, Davies PS, et al. Sedentary and Active Time in Toddlers with and without Cerebral Palsy. *Med Sci Sports Exerc*. 2015;47(10):2076-83.
5. Shkedy Rabani A, Harries N, Namoora I, et al. Duration and patterns of habitual physical activity in adolescents and young adults with cerebral palsy. *Dev Med Child Neurol*. 2014;56(7):673-80.
6. Keawutan P, Bell KL, Oftedal S, et al. Longitudinal physical activity and sedentary behaviour in preschool-aged children with cerebral palsy across all functional levels. *Dev Med Child Neurol*. 2017.
7. Maher A, Williams T, Olds T, et al. Physical and sedentary activity in adolescents with cerebral palsy. *Dev Med Child Neurol*. 2007;49(6):450.
8. Luther BL. Food and physical activity choices to prevent overweight in children with spina bifida. *Dissertation Abstracts International: Section B: The Sciences and Engineering*. 2010;71(6-B):3597.
9. Slavin RE. Best evidence synthesis: An intelligent alternative to meta-analysis. *Journal of Clinical Epidemiology*. 1995;48(1):9-18.
10. Grant MJ, Booth A. A typology of reviews: an analysis of 14 review types and associated methodologies. *Health Info Libr J*. 2009;26(2):91-108.
11. Cooke A, Smith D, Booth A. Beyond PICO: the SPIDER tool for qualitative evidence synthesis. *Qual Health Res*. 2012;22(10):1435-43.
12. The Joanna Briggs Institute. The Joanna Briggs Institute reviewers' manual 2015: methodology for JBI scoping reviews: The Joanna Briggs Institute 2015: 10-11.
13. The Endnote Team. EndNote. EndNote X7 ed. Philadelphia, PA: Clarivate; 2013.
14. Boutron I, Page M, Higgins J, et al. Considering bias and conflicts of interest among the included studies. In: Higgins J, Thomas J, Chandler J, et al., eds. Cochrane Handbook for Systematic Reviews of Interventions Version 62 (updated February 2021): Cochrane 2021.
15. National Heart Lung and Blood Institute. Study Quality Assessment Tools: U.S Department of Health& Human Services; 2017 [Available from: https://www.nhlbi.nih.gov/health-topics/study-quality-assessment-tools. Date Accessed 01/04/2021.
16. Critical Appraisal Skills Programme. CASP Checklists: CASP UK; 2020 [Available from: http://www.casp-uk.net/casp-tools-checklists. Date Accessed 01/04/2021.
17. Higgins JPT, Altman DG, Gøtzsche PC, et al. The Cochrane Collaboration's tool for assessing risk of bias in randomised trials. *British Medical Journal*. 2011;343:d5928.
18. Hong QN, Fàbregues Feijóo S, Bartlett G, et al. The Mixed Methods Appraisal Tool (MMAT) version 2018 for information professionals and researchers. *Education for information*. 2018.

EXCLUSIVE LICENCE

For peer review only - http://bmjopen.bmj.com/site/about/guidelines.xhtml

I, the Submitting Author has the right to grant and does grant on behalf of all authors of the Work (as defined in the below author licence), an exclusive licence and/or a non-exclusive licence for contributions from authors who are: i) UK Crown employees; ii) where BMJ has agreed a CC-BY licence shall apply, and/or iii) in accordance with the terms applicable for US Federal Government officers or employees acting as part of their official duties; on a worldwide, perpetual, irrevocable, royalty-free basis to BMJ Publishing Group Ltd ("BMJ") its licensees and where the relevant Journal is co-owned by BMJ to the co-owners of the Journal, to publish the Work in BMJ Open and any other BMJ products and to exploit all rights, as set out in our licence.

The Submitting Author accepts and understands that any supply made under these terms is made by BMJ to the Submitting Author unless you are acting as an employee on behalf of your employer or a postgraduate student of an affiliated institution which is paying any applicable article publishing charge ("APC") for Open Access articles. Where the Submitting Author wishes to make the Work available on an Open Access basis (and intends to pay the relevant APC), the terms of reuse of such Open Access shall be governed by a Creative Commons licence – details of these licences and which Creative Commons licence will apply to this Work are set out in our licence referred to above.

For peer review only

Sedentary Behaviour in Non-Ambulant Children and Young People with Physical Disabilities: A Systematic Search and Review Protocol

Search strategy developed using “Population, Concept, Context” framework.

Database	Population	Concept	Limits applied
**AMED - The Allied and Complementary Medicine Database Via EBSCOhost**	“physical disabilit*” (KW) OR “physically disabled” (KW) OR “non-ambulant” (KW) OR “non-ambulatory” (KW) OR wheelchair* (KW) OR “limited mobility” (KW) OR “immobil*”(KW) OR “mobility impairment” (KW) OR “neuromusculoskeletal disabilit*” (KW) OR amputee* (KW) OR “cerebral pals*” OR diplegi* (KW) OR quadripleg* (KW) OR tetrapleg* (KW) OR “muscular dystroph*” OR “spina bifida” (KW) OR “spinal dysraphism” (KW) OR “neural tube defect*” (KW) OR parapleg* (KW) paralys* (KW) OR “spinal cord injur*” OR handicap* (KW) OR “hereditary motor sensory neuropath*” (KW) OR “charcot-marie-tooth” (KW) OR "spinal muscular atroph*" (KW)<br><br>AND<br><br>minors (KW) OR pediatric (KW) OR paediatrics (KW) OR pediatric (KW) OR paediatric (KW) OR child (KW) OR children (KW) OR baby (KW) OR childhood (KW) OR babies (KW) OR infant (KW) OR infants (KW) OR infancy (KW) OR school (KW) OR preschool (KW) OR adolescen* (KW) OR teen* (KW) OR youth (KW) OR "young adult*" (KW) OR "young person*" (KW) OR "young people" (KW) OR toddler* (KW) OR juvenile* (KW)	sedentary (KW) OR “screen time" (KW) OR screentime (KW) OR "screen-time" (KW) OR "screen use" (KW) OR "screen exposure" (KW) OR (television or TV) NEAR/3 watch* OR "physical inactivity" (KW) OR "physically inactive" (KW) OR sitting (KW)	Published date: 1996 – 2021<br>Language: English
**Applied Social Sciences Index & Abstracts (ASSIA) Via ProQuest**			Publication date: 1996-2021<br>English language
**Child development and adolescent studies Via EBSCOhost**			Publication date: 1996 – 2021
**ERIC Via EBSCOhost**			Date Published: Jan 1996 – 2021<br>English Language
**SPORTDiscus Via EBSCOhost**			Publication Date: Jan 1996 – 2021<br>English Language
**APA PsycINFO Via Ovid**	“physical disabilit*” (KW) OR “physically disabled” (KW) OR “non-ambulant” (KW) OR “non-ambulatory” (KW) OR wheelchair* (KW) OR “limited mobility” (KW) OR “immobil*”(KW) OR “mobility impairment” (KW) OR “neuromusculoskeletal disability” (KW) (NB wouldn’t accept “neuromusculoskeletal disabilit*”) OR amputee*	sedentary (KW) OR “screen time" (KW) OR screentime (KW) OR "screen-time" (KW) OR "screen use" (KW) OR	Publication Year: 1996 – 2021<br>English language

	(KW) OR “cerebral pals*” OR diplegi* (KW) OR quadripleg* (KW) OR tetrapleg* (KW) OR “muscular dystroph*” OR “spina bifida” (KW) OR “spinal dysraphism” (KW) OR “neural tube defect*” (KW) OR parapleg* (KW) paralys* (KW) OR “spinal cord injur*” OR handicap* (KW) OR “hereditary motor sensory neuropath*” (KW) OR “charcot-marie-tooth” (KW) OR "spinal muscular atroph*" (KW)<br>OR “Physical disabilities (Attitudes toward)” (SH) (exp) OR Mobility aids (SH) (exp) OR Amputation (SH) (exp) OR Cerebral Palsy (SH) (exp) OR Quadriplegia (SH) (exp) OR Muscular Dystrophy (SH) (exp) OR Spina Bifida (SH) (exp) OR Paralysis (SH) (exp) OR Spinal Cord Injuries (SH) (exp)<br>OR Paragplegia (SH) (exp) OR Charcot-Marie-Tooth Disease (SH) (exp)<br><br>AND<br><br>minors OR pediatric OR paediatrics OR pediatrics OR paediatric OR child OR children OR baby OR childhood OR babies OR infant OR infants OR infancy OR school OR preschool OR adolescen* OR teen* OR youth OR "young adult*" OR "young person*" OR "young people" OR toddler* OR juvenile*	"screen exposure" (KW) OR (television or TV) adj3 watch* OR "physical inactivity" (KW) OR "physically inactive" (KW) OR sitting (KW) OR Sedentary behavior (SH) (exp) OR Television viewing (SH) (exp) OR Television (SH) (exp) OR Screen Time (SH) (exp)	
**CINAHL Plus via EBSCOhost**	“physical disabilit*” (KW) OR “physically disabled” (KW) OR “non-ambulant” (KW) OR “non-ambulatory” (KW) OR wheelchair* (KW) OR “limited mobility” (KW) OR “immobil*”(KW) OR “mobility impairment” (KW) OR “neuromusculoskeletal disabilit*” (KW) OR amputee* (KW) OR “cerebral pals*” OR diplegi* (KW) OR quadripleg* (KW) OR tetrapleg* (KW) OR “muscular dystroph*” OR “spina bifida” (KW) OR “spinal dysraphism” (KW) OR “neural tube defect*” (KW) OR parapleg* (KW) paralys* (KW) OR “spinal cord injur*” OR handicapped (KW) OR “hereditary motor sensory neuropath*” (KW) OR “charcot-marie-tooth” (KW) OR "spinal muscular atroph*" (KW) OR Immobility (SH) OR Wheelchairs (SH) (exp) OR Amputees (SH) (exp) OR Cerebral palsy (SH) OR Muscular Dystrophy (SH) (exp) OR Spina Bifida (SH) OR Neural Tube Defects (SH) (exp) OR Paraplegia (SH) (exp) OR Spastic Paraplegia, Hereditary (SH) OR Spinal Cord Injuries (SH) (exp) OR Quadriplegia (SH) (exp) OR Neuropathies, Hereditary motor and sensory (exp) OR Charcot-Marie-Tooth Disease (SH) OR Muscular Atrophy, Spinal (SH) (exp)<br><br>AND<br><br>minors (KW) OR pediatric (KW) OR paediatrics (KW) OR pediatric (KW) OR paediatric (KW) OR child (KW) OR	sedentary (KW) OR “screen time” (KW) OR screentime (KW) OR "screen-time" (KW) OR "screen use" (KW) OR "screen exposure" (KW) OR "(television or TV) near/3 watch* OR "physical inactivity" (KW) OR "physically inactive" (KW) OR Life Style, Sedentary (SH) (exp) OR Television (SH) OR Screen Time (SH) OR Sitting (SH)	Publication date: 1996 – 2021 Language: English

For peer review only - http://bmjopen.bmj.com/site/about/guidelines.xhtml

	children (KW) OR baby (KW) OR childhood (KW) OR babies (KW) OR infant (KW) OR infants (KW) OR infancy (KW) OR school (KW) OR preschool (KW) OR adolescen* (KW) OR teen* (KW) OR youth (KW) OR "young adult*" (KW) OR "young person*" (KW) OR "young people" (KW) OR toddler* (KW) OR juvenile* (KW) OR Infant, Newborn (SH) (exp) OR Minors (Legal) (SH) (exp) OR Child (SH) (exp) OR Adolescence (SH) (exp)		
**Cochrane Library**<br><br>**Via Cochrane library website**	Searched title and abstract only for:<br>“physical disability” OR “physical disabilities” OR “physically disabled” OR “non-ambulant” OR “non-ambulatory” OR wheelchair* OR “limited mobility” OR immobilit* OR “mobility impairment” OR “neuromusculoskeletal disabilit*” OR “neuromusculoskeletal disability” OR amputee* OR “cerebral palsy” OR diplegi* OR quadripleg* OR tetrapleg* OR “muscular dystrophy” OR “spina bifida” OR “spinal dysraphism” OR “neural tube defect” OR “neural tube defects” OR parapleg* OR paralys* OR “spinal cord injury” OR “spinal cord injuries” OR handicap* OR “hereditary motor sensory neuropathy” OR “hereditary motor sensory neuropathies” OR “charcot-marie-tooth” OR “spinal muscular atrophy” OR “spinal muscular atrophies” OR<br>Mobility Limitation (MeSH) (exp) OR Amputees (MeSH) (exp) OR<br>OR Wheelchairs (MeSH) (exp) OR<br>Cerebral Palsy (MeSH) (exp)<br>OR Muscular Dystrophies (MeSH) (exp)<br>OR Spinal Dysraphism (MeSH) (exp)<br>OR Neural Tube Defects (MeSH) (exp) OR Spinal Cord Injuries (MeSH) OR Paraplegia (MeSH) (exp) OR Quadriplegia (MeSH) (exp) OR Paralysis (MeSH) (exp) OR Spastic Paraplegia, Hereditary (MeSH) (exp) OR Hereditary Sensory and Motor Neuropathy (MeSH) (exp) OR Charvot-Marie-Tooth Disease (MeSH) (exp) OR Muscular Atrohy, Spinal (MeSH) (exp)<br><br>AND<br><br>minors OR pediatric OR paediatrics OR pediatrics OR paediatric OR child OR children OR baby OR childhood OR babies OR infant OR infants OR infancy OR school OR preschool OR adolescen* OR teen* OR youth OR "young adult" OR “young adults” OR "young person" OR "young people" OR toddler* OR juvenile*) OR Young Adult (MeSH) OR Adolescent (MeSH) OR (MeSH) OR Infant (MeSH) OR Child (MeSH)	Searched title and abstract only for:<br>sedentary (KW) OR “screen time" (KW) OR screentime (KW) OR "screen-time" (KW) OR "screen use" (KW) OR "screen exposure" (KW) OR (television or TV) near/3 watch* OR "physical inactivity" (KW) OR "physically inactive" (KW) OR Sedentary Behavior (MeSH) (exp) OR Television (MeSH) (exp) OR Sitting position (MeSH) (exp) OR Screen Time (MeSH) (exp)	Cochrane Library publication date:<br>Jan 1996 and 2021<br><br>Content type:<br>Cochrane reviews<br>Cochrane trials
**EMBASE: Excerpta Medica via Ovid**	“physical disabilit*” (KW) OR “physically disabled” (KW) OR “non-ambulant” (KW) OR “non-ambulatory” (KW) OR wheelchair* (KW) OR “limited mobility” (KW) OR	sedentary (KW) OR “screen time" (KW) OR screentime (KW)	Publication year: 1996 – 2021

For peer review only - http://bmjopen.bmj.com/site/about/guidelines.xhtml

	"immobil*"(KW) OR "mobility impairment" (KW) OR "neuromusculoskeletal disability" (KW) (NB wouldn't accept "neuromusculoskeletal disabilit*" OR amputee* (KW) OR "cerebral pals*" OR diplegi* (KW) OR quadripleg* (KW) OR tetrapleg* (KW) OR "muscular dystroph*" OR "spina bifida" (KW) OR "spinal dysraphism" (KW) OR "neural tube defect*" (KW) OR parapleg* (KW) paralys* (KW) OR "spinal cord injur*" OR handicap* (KW) OR "hereditary motor sensory neuropath*" (KW) OR "charcot-marie-tooth" (KW) OR "spinal muscular atroph*" (KW) OR Physical disability (SH) (exp) OR Wheelchair (SH) (exp) OR Limited Mobility (SH) (exp) OR Cerebral palsy (SH) (exp) (KW) OR Amputee (SH) (exp) OR Muscular dystrophy (SH) (exp) OR Duchenne Muscular Dystrophy (SH) (exp) OR Spina Bifida (SH) exp) OR Spinal Dysraphism (SH) (exp) OR Neural Tube Defect (SH) (exp) OR Paraplegia (SH) (exp) OR Paralysis (SH) (exp) OR Spastic paraplegia (SH) (exp) OR Quadriplegia (SH) (exp) OR Spinal cord injury (SH) (exp) OR Hereditary Motor Sensory Neuropathy (SH) (exp) OR Muscular Atrophy, Spinal (SH) (exp)<br><br>AND<br><br>minors (KW) OR pediatric (KW) OR paediatrics (KW) OR pediatric (KW) OR paediatric (KW) OR child (KW) OR children (KW) OR baby (KW) OR childhood (KW) OR babies (KW) OR infant (KW) OR infants (KW) OR infancy (KW) OR school (KW) OR preschool (KW) OR adolescen* (KW) OR teen* (KW) OR youth (KW) OR "young adult*" (KW) OR "young person*" (KW) OR "young people" (KW) OR toddler* (KW) OR juvenile* (KW) OR Young Adult (SH) (exp) OR Adolescent (SH) (exp) OR (SH) (exp) OR Infant (SH) (exp) OR Child (SH) (exp)	OR "screen-time" (KW) OR "screen use" (KW) OR "screen exposure" (KW) OR (television or TV) adj3 watch* OR "physical inactivity" (KW) OR "physically inactive" (KW) OR Sedentary lifestyle (SH) (exp) OR Sedentary time (SH) (exp) OR Screen time (SH) (exp) OR Television (SH) (exp) OR Television viewing (SH) (exp) OR Sitting (SH) (exp) OR Physical Inactivity (SH) (exp)	English language
**MEDLINE (R) Via Ovid**	"physical disabilit*" (KW) OR "physically disabled" (KW) OR "non-ambulant" (KW) OR "non-ambulatory" (KW) OR wheelchair* (KW) OR "limited mobility" (KW) OR "immobil*"(KW) OR "mobility impairment" (KW) OR "neuromusculoskeletal disabilit*" (KW) OR amputee* (KW) OR "cerebral pals*" OR diplegi* (KW) OR quadripleg* (KW) OR tetrapleg* (KW) OR "muscular dystroph*" OR "spina bifida" (KW) OR "spinal dysraphism" (KW) OR "neural tube defect*" (KW) OR parapleg* (KW) paralys* (KW) OR "spinal cord injur*" OR handicap* (KW) OR "hereditary motor sensory neuropath*" (KW) OR "charcot-marie-tooth" (KW) OR "spinal muscular atroph*" (KW) OR Wheelchairs (SH) (exp) OR Mobility Limitation (SH) (exp) OR Cerebral palsy (SH) (exp) (KW) OR Amputees (SH) (exp) OR Muscular dystrophies (SH) (exp) OR Spinal Dysraphism (SH) (exp) (KW) OR Neural Tube Defects (SH) (exp) OR	sedentary (KW) OR "screen time" (KW) OR screentime (KW) OR "screen-time" (KW) OR "screen use" (KW) OR "screen exposure" (KW) OR (television or TV) adj3 watch* OR "physical inactivity" (KW) OR "physically inactive" (KW) Sedentary Behavior (SH) (exp) OR Television (SH) (exp) OR Screen Time (SH) (exp) OR	Publication Year: 1996 – 2021 English language

For peer review only - http://bmjopen.bmj.com/site/about/guidelines.xhtml

	Paraplegia (SH) (exp) OR Paralysis (SH) (exp) OR Spastic paraplegia, hereditary (SH) (exp) OR Quadriplegia (SH) (exp) OR Spinal cord injuries (SH) (exp) OR Hereditary Sensory and Motor Neuropathy (SH) (exp) OR Charcot-Marie-Tooth Disease (SH) (exp) OR Muscular Atrophy, Spinal (SH) (exp)<br><br>AND<br><br>minors (KW) OR pediatric (KW) OR paediatrics (KW) OR pediatric (KW) OR paediatric (KW) OR child (KW) OR children (KW) OR baby (KW) OR childhood (KW) OR babies (KW) OR infant (KW) OR infants (KW) OR infancy (KW) OR school (KW) OR preschool (KW) OR adolescen* (KW) OR teen* (KW) OR youth (KW) OR "young adult*" (KW) OR "young person*" (KW) OR "young people" (KW) OR toddler* (KW) OR juvenile* (KW) OR Minors (SH) (exp) OR Child (SH) (exp) OR Child, Preschool (SH) (exp) OR Infant, Newborn (SH) (exp) OR Infant (SH) (exp) OR Adolescent (SH) (exp) OR Young Adult (SH) (exp)	Sitting Position (SH) (exp) OR	
**Nursing and Allied Health Database Via ProQuest**	"physical disabilit*" (KW) OR "physically disabled" (KW) OR "non-ambulant" (KW) OR "non-ambulatory" (KW) OR wheelchair* (KW) OR "limited mobility" (KW) OR "immobil*"(KW) OR "mobility impairment" (KW) OR "neuromusculoskeletal disabilit*" (KW) OR amputee* (KW) OR "cerebral pals*" OR diplegi* (KW) OR quadripleg* (KW) OR tetrapleg* (KW) OR "muscular dystroph*" OR "spina bifida" (KW) OR "spinal dysraphism" (KW) OR "neural tube defect*" (KW) OR parapleg* (KW) paralys* (KW) OR "spinal cord injur*" OR handicap* (KW) OR "hereditary motor sensory neuropath*" (KW) OR "charcot-marie-tooth" (KW) OR "spinal muscular atroph*" (KW) OR Neural Tube Defects (MeSH) OR Spastic Paraplegia, Hereditary (MeSH) OR Spina Bifida Occulta (MeSH) OR Amputees (MeSH) OR Quadriplegia (MeSH) OR Spina Bifida Cystica (MeSH) OR Charcot-Marie-Tooth Disease (MeSH) OR Mobility Limitation (MeSH) OR Paraplegia (MeSH) OR Muscular Dystrophies (MeSH) OR Spinal Cord Injuries (MeSH) OR Wheelchairs (MeSH) OR Muscular Atrophy, Spinal (MeSH) OR Spinal Dysraphism (MeSH) OR Hereditary Sensory & Motor Neuropathy (MeSH) OR Muscular Dystrophy, Duchenne (MeSH) OR Paralysis (MeSH) OR Cerebral Palsy (MeSH)<br><br>AND<br><br>minors (KW) OR pediatric (KW) OR paediatrics (KW) OR pediatric (KW) OR paediatric (KW) OR child (KW) OR children (KW) OR baby (KW) OR childhood (KW) OR	sedentary (KW) OR "screen time" (KW) OR screentime (KW) OR "screen-time" (KW) OR "screen use" (KW) OR "screen exposure" (KW) OR (television or TV) NEAR/3 watch* (KW) OR "physical inactivity" (KW) OR "physically inactive" (KW) OR sitting (KW) OR Sedentary lifestyle (MeSH) OR Television (MeSH)	Publication date: 01/01/1996 – 2021 English language only

For peer review only - http://bmjopen.bmj.com/site/about/guidelines.xhtml

	babies (KW) OR infant (KW) OR infants (KW) OR infancy (KW) OR school (KW) OR preschool (KW) OR adolescen* (KW) OR teen* (KW) OR youth (KW) OR "young adult*" (KW) OR "young person*" (KW) OR "young people" (KW) OR toddler* (KW) OR juvenile* (KW) OR Young Adult (MeSH) OR Adolescent (MeSH) OR (MeSH) OR Infant (MeSH) OR Child (MeSH)		
**Scopus**	Title and abstract search only (not key words). “physical disability*” (KW) OR “physically disabled” (KW) OR “non-ambulant” (KW) OR “non-ambulatory” (KW) OR wheelchair* (KW) OR “limited mobility” (KW) OR “immobil*”(KW) OR “mobility impairment” (KW) OR “neuromusculoskeletal disabilit*” (KW) OR amputee* (KW) OR “cerebral pals*” OR diplegi* (KW) OR quadripleg* (KW) OR tetrapleg* (KW) OR “muscular dystroph*” OR “spina bifida” (KW) OR “spinal dysraphism” (KW) OR “neural tube defect*” (KW) OR parapleg* (KW) paralys* (KW) OR “spinal cord injur*” OR handicap* (KW) OR “hereditary motor sensory neuropath*” (KW) OR “charcot-marie-tooth” (KW) OR "spinal muscular atroph*" (KW)<br><br>AND<br><br>minors (KW) OR pediatric (KW) OR paediatrics (KW) OR pediatric (KW) OR paediatric (KW) OR child (KW) OR children (KW) OR baby (KW) OR childhood (KW) OR babies (KW) OR infant (KW) OR infants (KW) OR infancy (KW) OR school (KW) OR preschool (KW) OR adolescen* (KW) OR teen* (KW) OR youth (KW) OR "young adult*" (KW) OR "young person*" (KW) OR "young people" (KW) OR toddler* (KW) OR juvenile* (KW)	Title and abstract search only (not key words). sedentary (KW) OR “screen time" (KW) OR screentime (KW) OR "screen-time" (KW) OR "screen use" (KW) OR "screen exposure" (KW) OR (television or TV) NEAR/3 watch* OR "physical inactivity" (KW) OR "physically inactive" (KW) OR sitting	Publication year: 1996-2021 English language
**Web of Science Core Collection**	Searched Title and Abstract for: “physical disabilit*” OR “physically disabled” OR “non-ambulant” OR “non-ambulatory” OR wheelchair* OR “limited mobility” OR immobil* OR “mobility impairment” OR “neuromusculoskeletal disabilit*” OR amputee* OR “cerebral pals*” OR diplegi* OR quadripleg* OR tetrapleg* OR “muscular dystroph*” OR “spina bifida” OR “spinal dysraphism” OR “neural tube defect*” OR parapleg* OR paralys* OR “spinal cord injur*” OR handicap* OR “hereditary motor sensory neuropath*” OR “charcot-marie-tooth” OR “spinal muscular atroph*”<br><br>AND<br><br>minors OR pediatric OR paediatrics OR pediatrics OR paediatric OR child OR children OR baby OR childhood OR babies OR infant OR infants OR infancy OR school OR	Searched Title and Abstract for: sedentary OR “screen time" OR screentime OR "screen-time" OR "screen use" OR "screen exposure" OR (television or TV) NEAR/3 watch* OR "physical inactivity" OR "physically inactive" OR sitting	Custom year range 1996-2021 English language

For peer review only

	preschool OR adolescen* OR teen* OR youth OR "young adult*" OR "young person*" OR "young people" OR toddler* OR juvenile*		

For peer review only

For peer review only - http://bmjopen.bmj.com/site/about/guidelines.xhtml

PRISMA-P (Preferred Reporting Items for Systematic review and Meta-Analysis Protocols) 2015 checklist: recommended items to address in a systematic review protocol*

Section and topic	Item No	Checklist item	Page Number
**ADMINISTRATIVE INFORMATION**			
Title:			
Identification	1a	Identify the report as a protocol of a systematic review	1
Update	1b	If the protocol is for an update of a previous systematic review, identify as such	
Registration	2	If registered, provide the name of the registry (such as PROSPERO) and registration number	12
Authors:			
Contact	3a	Provide name, institutional affiliation, e-mail address of all protocol authors; provide physical mailing address of corresponding author	1, 12
Contributions	3b	Describe contributions of protocol authors and identify the guarantor of the review	
Amendments	4	If the protocol represents an amendment of a previously completed or published protocol, identify as such and list changes; otherwise, state plan for documenting important protocol amendments	NA
Support:			
Sources	5a	Indicate sources of financial or other support for the review	
Sponsor	5b	Provide name for the review funder and/or sponsor	
Role of sponsor or funder	5c	Describe roles of funder(s), sponsor(s), and/or institution(s), if any, in developing the protocol	12-13
**INTRODUCTION**			
Rationale	6	Describe the rationale for the review in the context of what is already known	3-5
Objectives	7	Provide an explicit statement of the question(s) the review will address with reference to participants, interventions, comparators, and outcomes (PICO)	4-7 using PCC rather than PICO
**METHODS**			
Eligibility criteria	8	Specify the study characteristics (such as PICO, study design, setting, time frame) and report characteristics (such as years considered, language, publication status) to be used as criteria for eligibility for the review	5-7
Information sources	9	Describe all intended information sources (such as electronic databases, contact with study authors, trial registers or other grey literature sources) with planned dates of coverage	4-9

For peer review only

Search strategy	10	Present draft of search strategy to be used for at least one electronic database, including planned limits, such that it could be repeated	7-8 and supplementary file 1
Study records:			
Data management	11a	Describe the mechanism(s) that will be used to manage records and data throughout the review	8-9
Selection process	11b	State the process that will be used for selecting studies (such as two independent reviewers) through each phase of the review (that is, screening, eligibility and inclusion in meta-analysis)	9
Data collection process	11c	Describe planned method of extracting data from reports (such as piloting forms, done independently, in duplicate), any processes for obtaining and confirming data from investigators	9-11
Data items	12	List and define all variables for which data will be sought (such as PICO items, funding sources), any pre-planned data assumptions and simplifications	10-11
Outcomes and prioritization	13	List and define all outcomes for which data will be sought, including prioritization of main and additional outcomes, with rationale	9-11
Risk of bias in individual studies	14	Describe anticipated methods for assessing risk of bias of individual studies, including whether this will be done at the outcome or study level, or both; state how this information will be used in data synthesis	9-12
Data synthesis	15a	Describe criteria under which study data will be quantitatively synthesised	11-12
	15b	If data are appropriate for quantitative synthesis, describe planned summary measures, methods of handling data and methods of combining data from studies, including any planned exploration of consistency (such as I^2, Kendall's τ)	Potential to be assessed as part of data analysis
	15c	Describe any proposed additional analyses (such as sensitivity or subgroup analyses, meta-regression)	NA
	15d	If quantitative synthesis is not appropriate, describe the type of summary planned	Appropriateness to be assessed as part of analysis
Meta-bias(es)	16	Specify any planned assessment of meta-bias(es) (such as publication bias across studies, selective reporting within studies)	NA
Confidence in cumulative evidence	17	Describe how the strength of the body of evidence will be assessed (such as GRADE)	9-12

*** It is strongly recommended that this checklist be read in conjunction with the PRISMA-P Explanation and Elaboration (cite when available) for important clarification on the items. Amendments to a review protocol should be tracked and dated.** **The copyright for PRISMA-P (including checklist) is held by the PRISMA-P Group and is distributed under a Creative Commons Attribution Licence 4.0.**

For peer review only

From: Shamseer L, Moher D, Clarke M, Ghersi D, Liberati A, Petticrew M, Shekelle P, Stewart L, PRISMA-P Group. Preferred reporting items for systematic review and meta-analysis protocols (PRISMA-P) 2015: elaboration and explanation. BMJ. 2015 Jan 2;349(jan02 1):g7647.

For peer review only

Sedentary Behaviour in Non-Ambulant Children and Young People with Physical Disabilities: A Systematic Search and Review Protocol

Journal:	*BMJ Open*
Manuscript ID	bmjopen-2021-053077.R2
Article Type:	Protocol
Date Submitted by the Author:	22-Oct-2021
Complete List of Authors:	Bradbury, Marilyn; Birmingham Community Healthcare NHS Foundation Trust, Research and Innovation; University of Birmingham, School of Sport, Exercise and Rehabilitation Sciences<br>O'Brien, Ciara; University of Surrey, School of Psychology<br>Giles, Nathan; Birmingham Community Healthcare NHS Foundation Trust, Public and Patient Involvement Representative (Volunteer)<br>Fenton, Sally; University of Birmingham, School of Sport, Exercise and Rehabilitation Sciences<br>Neilson, Sue; University of Birmingham, School of Nursing<br>Duda, Joan; University of Birmingham, School of Sport, Exercise & Rehabilitation Sciences
**Primary Subject Heading**:	Rehabilitation medicine
Secondary Subject Heading:	Sports and exercise medicine
Keywords:	Developmental neurology & neurodisability < PAEDIATRICS, REHABILITATION MEDICINE, SOCIAL MEDICINE

I, the Submitting Author has the right to grant and does grant on behalf of all authors of the Work (as defined in the below author licence), an exclusive licence and/or a non-exclusive licence for contributions from authors who are: i) UK Crown employees; ii) where BMJ has agreed a CC-BY licence shall apply, and/or iii) in accordance with the terms applicable for US Federal Government officers or employees acting as part of their official duties; on a worldwide, perpetual, irrevocable, royalty-free basis to BMJ Publishing Group Ltd ("BMJ") its licensees and where the relevant Journal is co-owned by BMJ to the co-owners of the Journal, to publish the Work in this journal and any other BMJ products and to exploit all rights, as set out in our licence.

The Submitting Author accepts and understands that any supply made under these terms is made by BMJ to the Submitting Author unless you are acting as an employee on behalf of your employer or a postgraduate student of an affiliated institution which is paying any applicable article publishing charge ("APC") for Open Access articles. Where the Submitting Author wishes to make the Work available on an Open Access basis (and intends to pay the relevant APC), the terms of reuse of such Open Access shall be governed by a Creative Commons licence – details of these licences and which Creative Commons licence will apply to this Work are set out in our licence referred to above.

Other than as permitted in any relevant BMJ Author's Self Archiving Policies, I confirm this Work has not been accepted for publication elsewhere, is not being considered for publication elsewhere and does not duplicate material already published. I confirm all authors consent to publication of this Work and authorise the granting of this licence.

For peer review only

TITLE: SEDENTARY BEHAVIOUR IN NON-AMBULANT CHILDREN AND YOUNG PEOPLE WITH PHYSICAL DISABILITIES: A SYSTEMATIC SEARCH AND REVIEW PROTOCOL

AUTHOR INFORMATION

Corresponding author: Marilyn Bradbury

Affiliations: Birmingham Community Healthcare NHS Foundation Trust, School of Sport, Exercise and Rehabilitation Sciences, University of Birmingham.

Postal address: Research and Innovation, Birmingham Community Healthcare NHS Foundation Trust, Trust Headquarters, 3 Priestley Wharf, 20 Holt Street, Birmingham, B7 4BN, United Kingdom

email: marilyn.bradbury1@nhs.net

ORCID ID: 0000-0001-6000-7229

Ciara O'Brien, School of Psychology, University of Surrey, Surrey, UK. ciara.o'brien@surrey.ac.uk

ORCID ID: 0000-0002-2215-6001

Nathan Giles, Lay member of the DoMore Study Steering Group. Birmingham, UK. NathanGiles20@outlook.com,

Sally A.M. Fenton, School of Sport, Exercise and Rehabilitation Sciences, University of Birmingham, Birmingham, UK S.A.M.Fenton@bham.ac.uk

Sue Neilson, School of Nursing, University of Birmingham, Birmingham, UK. S.J.Neilson@bham.ac.uk

Joan L. Duda, School of Sport, Exercise and Rehabilitation Sciences, University of Birmingham, Birmingham, UK J.L.DUDA@bham.ac.uk

Word count: 2, 576

For peer review only

For peer review only - http://bmjopen.bmj.com/site/about/guidelines.xhtml

ABSTRACT

Introduction:

Non-ambulant children and young people with physical disabilities are at high risk of experiencing negative health outcomes associated with sedentary time. A previous scoping review summarising evidence relating to sedentary behaviours of children with physical disabilities identified the need for validated methods of measuring physical activity of children who use wheelchairs and evaluation of interventions to reduce sedentary time. The scoping review did not assess the quality of evidence relating to this topic, therefore its validity remains unclear. No reviews focussing on non-ambulant children and young people up to the age of 25 years have been undertaken.

The objectives of this systematic search and review are to:

- Identify all peer reviewed articles relating to sedentary behaviour of non-ambulant children and young people.
- Categorise the articles according to study design and 4 sub questions relating to 1) measurement 2) patterns 3) associated risks and 4) interventions to reduce sedentary time or behaviour.
- Critically appraise quality of the articles using established critical appraisal tools.
- Summarise the evidence for each sub question. Describe its cumulative strength and identify knowledge gaps.

Methods and Analysis:

This protocol was developed using the PRISMA-P checklist. The research questions, inclusion / exclusion criteria and search terms have been developed a priori using the "Population, Concept and Context" framework. Online databases will be systematically searched to identify peer reviewed articles published between 1996 and 2021. Two reviewers will screen, categorise and critically appraise the articles. Data extraction and analysis will be verified by the second reviewer.

Results will be reported as a best evidence synthesis, with reference to the PRISMA checklist.

Ethics and Dissemination:

Ethical approval is not required. The review will be submitted to an appropriate peer reviewed journal.

Registration

The review is registered on the Open Science Framework database.

KEYWORDS

Sedentary behaviour, physical disability, children, young people, wheelchair, non-ambulant, systematic search and review.

ARTICLE SUMMARY

STRENGTHS AND LIMITATIONS OF THIS STUDY

For peer review only

For peer review only - http://bmjopen.bmj.com/site/about/guidelines.xhtml

- This review systematically identifies evidence relating to sedentary behaviours of non-ambulant children and young people using a robust and comprehensive search strategy.
- The review builds on previously published literature through the addition of an assessment of the quality of the included research.
- A wide age range is incorporated, allowing consideration of how sedentary behaviour changes on the journey from childhood into adulthood.
- A limitation is that only peer-reviewed literature will be included.
- A limitation is that only studies which are written in English will be included.

INTRODUCTION

Sedentary behaviour is defined as "any waking behaviour characterized by an energy expenditure ≤1.5 metabolic equivalents (METs) while in a sitting or reclining posture",[1]. A large body of evidence from a variety of study designs suggests that reducing sedentary time is associated with lower health risk in children and young people aged 5-17 years,[2]. This body of evidence includes studies measuring both sedentary time and specific behaviours such as television watching. In children, time spent watching television has been associated with increased body mass index, reduced physical fitness, low self-esteem and reduced academic achievement,[2].

Children with disabilities are more sedentary than their able bodied peers,[3]. Lower levels of gross motor function,[4-6] and increasing age,[6-8] , are linked to increased sedentary behaviours in children with disabilities. Young people who are non-ambulant (i.e., unable to walk, or can walk short distances using a body support walker ["therapeutic walking"]), are therefore at greatest risk of experiencing negative health outcomes associated with sedentary time. Young people in this group may have conditions such as (but not limited to) cerebral palsy, spina bifida, muscular dystrophy, irreversible traumatic injuries or rare conditions.

A scoping review of sedentary behaviour and children with physical disabilities has recently summarised research relating to measurement of sedentary time, sedentary behaviour patterns, and interventions to reduce or break up time spent sedentary,[3]. The review identified that there is a lack of research evaluating interventions to decrease the sedentary behaviours of children with physical disabilities. In addition, the need for research that focuses on improving our understanding of sedentary behaviour patterns in children who are non-ambulant, including the validity of using accelerometers to measure sedentary time in this group, was recognised. To date, no reviews incorporating a quality assessment of research relating to this topic or focussing specifically on non-ambulant children and young people have been undertaken. This review builds on that of Ganz et al., (2020),[3] by focusing specifically on non-ambulant children and young people, incorporating critical evaluation of the quality of the evidence, considering risks associated with sedentary time or behaviour for this population and searching a wider range of databases. Contemporary work that has been published since the searches for the scoping review were undertaken will also be captured. The present review is intended to inform the development of an intervention which aims to reduce sedentary behaviour in non-ambulant young people with long-term disabilities. The targeted age range for the planned intervention includes young people aged 12 - 25 years. The present review will include research relating to a wide age range, from birth to 25 years. This extends the age range of the recent scoping review to include 18-25 year olds and summarises the evidence throughout

For peer review only - http://bmjopen.bmj.com/site/about/guidelines.xhtml

children's development. Covering this wide age range provides a broad perspective that allows an in depth understanding of children's behaviour, taking into consideration how sedentary behaviour changes throughout the journey from childhood into adulthood. This captures key transition points that may have an impact on sedentary behaviour such as leaving school and moving from children's to adult's health services.

The objectives of the review are to:

1) Identify all peer reviewed journal articles relating to measurement of sedentary time, patterns of sedentary behaviour and interventions to decrease sedentary time or behaviours for non-ambulant children and young people.
2) Categorise the articles according to study design and 4 sub questions relating to 1) measurement 2) patterns 3) associated risks and 4) interventions to reduce sedentary time or behaviour for non-ambulant children and young people.
3) Critically appraise the quality of the articles using appropriate tools to evaluate the study design.
4) Produce a best evidence synthesis,[9, 10] which summarises the evidence for each sub question and describes its cumulative strength, based on the hierarchy of evidence and the quality ratings assigned following critical appraisal. Identify knowledge gaps for this specific population.

The overarching research question is "What is the quality of the evidence relating to sedentary behaviour in non-ambulant children and young people?". The sub-questions are:

1) Do validated measures of sedentary time or behaviour of non-ambulant children and young people exist?
2) Have patterns of sedentary time or behaviour of non-ambulant children and young people been described?
3) Have risks associated with sedentary time or behaviour been explored or quantified for non-ambulant children and young people?
4) What evidence-based interventions to reduce sedentary behaviour or decrease and break up sedentary time in this population have been evaluated and what is the strength of this evidence?

Grant and Booth,[10] have described the typology of the systematic search and review. They highlight how this type of review combines a systematic search method with a critical review analysis. This review typology is used to answer broad questions and often incorporates multiple study designs. The analysis results in a best evidence synthesis to describe what is known, recommendations for practice and limitations of the evidence. A systematic search and review typology is being adopted for this review because the topic is too broad to undertake a traditional systematic review, which should focus on only one specific question. The incorporation of several sub-questions and the inclusion of studies from multiple paradigms with widely varying study designs makes a systematic review methodology inappropriate,[10].

METHODS AND ANALYSIS

For peer review only - http://bmjopen.bmj.com/site/about/guidelines.xhtml

Search Strategy

Databases searched by Ganz et al., (2020),[3] were CINAHL, MEDLINE, ERIC, EMBASE and SPORTDiscus. The electronic databases searched in this review include the following additional databases, on a variety of platforms to expand the breadth of the search: The Allied and Complementary Medicine Database (AMED), APA PsycINFO, Applied Social Sciences Index & Abstracts (ASSIA), Child development and adolescent studies, CINAHL Plus, Cochrane Library, EMBASE: Excerpta, ERIC, MEDLINE (R), Nursing and Allied Health Database, Scopus, SPORTDiscus and Web of Science.

The searches for this review will be limited to publication dates between January 1996 – September 2021, as the majority of the relevant publications identified by Ganz et al.,[3] were published after 2013 (72%), with the earliest included paper being published in 1996. Searches were undertaken by Ganz et al.,[3] in November 2018, so new papers published between November 2018 and September 2021 will be screened in this review. Reference lists of eligible articles will also be screened for any relevant articles that may have been missed in the database searches.

Traditional tools used to produce search strategies in systematic reviews include the PICO tool (which is ideally suited to quantitative studies) and the SPIDER tool (which is suitable for qualitative and mixed methods research),[11]. In this review, quantitative, qualitative, mixed methods studies and reviews are all relevant. Therefore, the "Population, Concept and Context" (PCC) framework recommended for use in scoping reviews, has been adopted,[12]. The PCC framework is more appropriate than the PICO tool to capture the wide range of study designs associated with multiple sub-questions. The inclusion and exclusion criteria developed using the PCC framework is outlined in Table 1 below.

For peer review only

For peer review only - http://bmjopen.bmj.com/site/about/guidelines.xhtml

Table 1 Inclusion and exclusion criteria. PCC = Population, Concept, Context. GMFCS = Gross Motor Function Classification System.

PCC framework element	Inclusion	Exclusion
**Population**	Participants with long term physical disabilities.<br>AND<br>A proportion of the participants are non-ambulant. The proportion of non-ambulant participants is clear.<br>For participants with cerebral palsy, children and young people in Gross Motor Function Classification System (GMFCS) levels IV-V are considered non-ambulant.<br>AND<br>Article includes participants under 25 years.	Participants with intellectual disabilities, visual or hearing impairments and no physical disability.<br>OR<br>Participants with a temporary lack of ambulation due to injury or short-term illness.<br>OR<br>The article includes ambulant participants only, or the proportion of non-ambulant participants is unclear.<br>For participants with cerebral palsy, children and young people in GMFCS levels I-III are considered ambulant.<br>OR<br>All participants in the article are 25 and over
**Concept**	Article explores measurement of, patterns of, or risks related to sedentary behaviour.<br>OR<br>Article evaluates feasibility or effectiveness of an intervention to decrease or break up sedentary time.<br>NB any outcome measured in studies matching the inclusion criteria will be accepted.	Papers relating to physical activity that have not measured or explored patterns or correlates of sedentary time or behaviour specifically.
**Context**	Peer reviewed journal articles (quantitative, qualitative, mixed methods), scoping reviews, systematic reviews, theses / dissertations<br>AND<br>Published in the English language.<br>AND<br>Published between January 1996 and September 2021.	Grey literature, conference abstracts, conference proceedings, conference papers, conference review, protocols, commentaries, opinion articles, literature reviews (not systematic), narrative reviews, books or chapters.<br>OR<br>Published in languages other than English.<br>OR

view only

For peer review only - http://bmjopen.bmj.com/site/about/guidelines.xhtml

		Published before 1996 or after September 2021.

As quality rather than scope of the evidence is the focus of this review, grey literature has been excluded. The search strategy and examples of the search terms used in two of the databases (one which offers subject heading searches and a second which does not) are shown in Table 2. Please refer to the supplementary material (File 1) for the full search strategy. Development of the search strategy terms was guided by those adopted by Ganz et al., (2020),[3], however, additional terms have been included to ensure the search is specific to the overarching research question and the sub-questions outlined above. Iterative cycles of trying different terms were carried out in order to decide upon the terms that yielded the most appropriate results and to ensure the terms were suitable across the wide range of databases.

Table 2. Search strategy using PCC framework. exp = exploded; SH = subject heading; KW = keyword in title or abstract. NB where subject headings were not exploded, this is because this option was not available.

Database	Population	Concept	Limits applied
**AMED - The Allied and Complementary Medicine Database**<br><br>**Via EBSCOhost**	“physical disabilit*” (KW) OR “physically disabled” (KW) OR “non-ambulant” (KW) OR “non-ambulatory” (KW) OR wheelchair* (KW) OR “limited mobility” (KW) OR “immobil*”(KW) OR “mobility impairment” (KW) OR “neuromusculoskeletal disabilit*” (KW) OR amputee* (KW) OR “cerebral pals*” OR diplegi* (KW) OR quadripleg* (KW) OR tetrapleg* (KW) OR “muscular dystroph*” OR “spina bifida” (KW) OR “spinal dysraphism” (KW) OR “neural tube defect*” (KW) OR parapleg* (KW) paralys* (KW) OR “spinal cord injur*” OR handicap* (KW) OR “hereditary motor sensory neuropath*” (KW) OR “charcot-marie-tooth” (KW) OR "spinal muscular atroph*" (KW)<br><br>AND<br><br>minors (KW) OR pediatric (KW) OR paediatrics (KW) OR pediatric (KW) OR paediatric (KW) OR child (KW) OR children (KW) OR baby (KW) OR childhood (KW) OR babies (KW) OR infant (KW) OR infants (KW) OR infancy	sedentary (KW) OR “screen time" (KW) OR screentime (KW) OR "screen-time" (KW) OR "screen use" (KW) OR "screen exposure" (KW) OR (television or TV) NEAR/3 watch* OR "physical inactivity" (KW) OR "physically inactive" (KW) OR sitting (KW)	Published date: 1996 – 2021<br>Language: English

For peer review only - http://bmjopen.bmj.com/site/about/guidelines.xhtml

	(KW) OR school (KW) OR preschool (KW) OR adolescen* (KW) OR teen* (KW) OR youth (KW) OR "young adult*" (KW) OR "young person*" (KW) OR "young people" (KW) OR toddler* (KW) OR juvenile* (KW)		
**EMBASE: Excerpta Medica via Ovid**	"physical disabilit*" (KW) OR "physically disabled" (KW) OR "non-ambulant" (KW) OR "non-ambulatory" (KW) OR wheelchair* (KW) OR "limited mobility" (KW) OR "immobil*"(KW) OR "mobility impairment" (KW) OR "neuromusculoskeletal disability" (KW) (NB wouldn't accept "neuromusculoskeletal disabilit*" OR amputee* (KW) OR "cerebral pals*" OR diplegi* (KW) OR quadripleg* (KW) OR tetrapleg* (KW) OR "muscular dystroph*" OR "spina bifida" (KW) OR "spinal dysraphism" (KW) OR "neural tube defect*" (KW) OR parapleg* (KW) paralys* (KW) OR "spinal cord injur*" OR handicap* (KW) OR "hereditary motor sensory neuropath*" (KW) OR "charcot-marie-tooth" (KW) OR "spinal muscular atroph*" (KW) OR Physical disability (SH) (exp) OR Wheelchair (SH) (exp) OR Limited Mobility (SH) (exp) OR Cerebral palsy (SH) (exp) (KW) OR Amputee (SH) (exp) OR Muscular dystrophy (SH) (exp) OR Duchenne Muscular Dystrophy (SH) (exp) OR Spina Bifida (SH) exp) OR Spinal Dysraphism (SH) (exp) OR Neural Tube Defect (SH) (exp) OR Paraplegia (SH) (exp) OR Paralysis (SH) (exp) OR Spastic paraplegia (SH) (exp) OR Quadriplegia (SH) (exp) OR Spinal cord injury (SH) (exp) OR Hereditary Motor Sensory Neuropathy (SH) (exp) OR Muscular Atrophy, Spinal (SH) (exp)<br><br>AND<br><br>minors (KW) OR pediatric (KW) OR paediatrics (KW) OR pediatric (KW) OR paediatric (KW) OR child (KW) OR children (KW) OR baby (KW) OR childhood (KW) OR babies (KW) OR infant (KW) OR infants (KW) OR infancy (KW) OR school (KW) OR preschool (KW) OR adolescen* (KW) OR teen* (KW) OR youth (KW) OR "young adult*" (KW) OR "young person*" (KW) OR "young people" (KW) OR toddler* (KW) OR juvenile* (KW) OR Young Adult (SH) (exp) OR Adolescent (SH) (exp) OR (SH) (exp) OR Infant (SH) (exp) OR Child (SH) (exp)	sedentary (KW) OR "screen time" (KW) OR screentime (KW) OR "screen-time" (KW) OR "screen use" (KW) OR "screen exposure" (KW) OR (television or TV) adj3 watch* OR "physical inactivity" (KW) OR "physically inactive" (KW) OR Sedentary lifestyle (SH) (exp) OR Sedentary time (SH) (exp) OR Screen time (SH) (exp) OR Television (SH) (exp) OR Television viewing (SH) (exp) OR Sitting (SH) (exp) OR Physical Inactivity (SH) (exp)	Publication year: 1996 – current English language

Searches for each group of terms defining the "Population" and the "Concept" (Table 1) will be conducted on separate lines in the databases, with individual terms combined as shown in Table 2.

For peer review only - http://bmjopen.bmj.com/site/about/guidelines.xhtml

The results from these searches will then be combined using AND. The limits outlined in Table 2 were applied to the search results prior to exporting citations to Endnote reference management software,[13].

Final searches will be conducted in September 2021.Our planned timelines for the study are as follows: screening of papers for inclusion will be carried out between September and November 2021, appraisal of included articles and data extraction between December and April 2022 and production of the best evidence synthesis and writing of the review article between May and July 2022.

Management of Study Records

All citations from the individual databases searched will be exported into a bibliographic management software program,[13]. Records from the individual database searches will be combined and duplicates removed. The titles and abstracts will be screened for relevance by two independent reviewers using the inclusion and exclusion criteria outlined in Table 1. Articles will be rated as relevant, not relevant, or not known. Articles will be rated as not known if there is insufficient information in the abstract to decide its relevance, or if the reviewer is uncertain of whether or not it fits the inclusion criteria. Full text articles will be obtained for all articles deemed to be “relevant” or “not known” following screening. Any discrepancies between the reviewers in relation to the inclusion of articles will be discussed following review of the full text and a consensus agreed. A third reviewer will be available to review full text articles for which agreement cannot be reached between the first and second reviewers.

Assessing quality of the evidence.

The internal validity of each article will be assessed using an appropriate tool. Quality and risk of bias will, therefore be assessed at study, but not outcome level. Full text articles will be evaluated by two independent reviewers. An overall rating of the quality of each article (good, fair, poor or unclear) will be assigned by both reviewers, based on a risk of bias assessment using a collection of critical appraisal tools. If there are disagreements between the two reviewers that cannot be reconciled through discussion, the third reviewer will be consulted. Any articles rated as “unclear” by either reviewer will also be discussed by the two independent reviewers and if required referred to the third reviewer to rate the quality of the article as good, fair or poor.

The Cochrane handbook explains that studies at high risk of bias should be given reduced weight in comparison to studies at low risk of bias,[14]. The flaws that exist in studies with a high risk of bias cannot be disregarded in the best evidence synthesis we will produce. It is important to find a balance between bias and precision. Including all studies in the review, regardless of their quality offers a high level of precision but serious risk of bias. Only including high quality studies with low risk of bias reduces the precision of the evidence synthesis. The Cochrane Handbook suggests four strategies authors can select from to present their results meaningfully,[14]. These strategies relate to systematic reviews of interventions and therefore focus on management of quantitative data from randomised control trials in meta-analysis. The scoping review previously undertaken gives an indication of the designs of studies that are likely to be included in this review,[3]. Thirteen articles in the existing review included non-ambulant participants. Their designs were described as: cross-sectional or cross-sectional survey, longitudinal, pre-post test, prospective cohort and

For peer review only - http://bmjopen.bmj.com/site/about/guidelines.xhtml

validation studies. As they are not randomised control trials of interventions, the strategies recommended in the Cochrane handbook are therefore difficult to apply in this review. The strategy we will adopt is as follows; to minimise serious risk of bias, articles rated as being poor quality will be excluded from this review. Articles rated as good or fair quality will be accepted for data extraction and inclusion in the evidence synthesis but their quality rating will be taken into account.

Reviewers will independently categorise each article by sub question and design.

The work of Ganz et al.,[3] gives an indication of the types of critical appraisal tools that will be required to assess risk of bias. The following critical appraisal tools will therefore be used to evaluate the quality of these studies:

The Quality Assessment Tool for Observational Cohort and Cross-Sectional Studies,[15]

The Quality Assessment Tool for Before-After (Pre-Post) Studies With No Control Group,[15]

If additional systematic reviews, randomised control trials, mixed methods studies or qualitative studies are identified, the following tools will be used to assess quality and risk of bias:

The CASP Systematic Review Checklist,[16]

The Cochrane Collaboration’s tool for assessing risk of bias in randomised trials,[17]

The Mixed Methods Appraisal Tool,[18]

The CASP Qualitative Checklist,[16]

Data Extraction

One reviewer will undertake data extraction for included articles. The second reviewer will check the accuracy of this data extraction. Table 3 shows the data items which will be extracted from each article. If the data items listed below are not included in the article, the corresponding author will be contacted twice to request this information. If no response is received, it will be marked as unavailable.

For peer review only

Table 3. Data extraction items.

Data Item								
Author (year) (ref)<br><br>Country of origin?	Participants*	Objectives	Design	Intervention (Sub-quesiton 3 studies only)	Methods**	Results (specific to sedentary behaviour)	Critical appraisal tool	Quality of article (good, fair, poor)
*Participant data will include total number of participants, number of non-ambulant participants, diagnosis, age range, mean age and standard deviation. GMFCS level / level of impairment of participants if available.<br>**Methods will include type of measure or data collection method (e.g. gas analysis, accelerometer make and model, patient report (survey, interview, validated patient reported outcome measure), protocol (e.g. placement of accelerometer, accelerometer cut point used, wear protocol), data reduction if applicable (e.g. cut-points), outcome variable used in study if applicable (e.g. Sedentary time, sedentary behaviour (total or min/day)								

Results

The search results will be presented in a PRISMA flowchart. The flowchart will show the number of articles identified, the number of duplications and articles included and excluded at each stage, the reasons for exclusions and number of articles excluded for each reason. Four tables will be produced to summarise the evidence from included articles, one for each of the sub questions, containing the data items described in Table 3.

Public and Patient Involvement

Three public and patient involvement representatives have reviewed this protocol and provided feedback. They will also follow the progress of the review, having oversight of and providing feedback on our processes of screening the papers, assessing quality of relevant articles and disseminating the results. One of these three representatives has also kindly agreed to act as the third reviewer.

Best evidence synthesis

Slavin,[9] describes how producing a best evidence synthesis allows reviewers propose conclusions or hypotheses about what we can learn from a body of evidence in relation to a particular topic in situations where there are studies carried out by a variety of research teams, involving different populations and methods to examine the same phenomenon. There may also be a lack of consensus in the conclusions of these studies. The best evidence synthesis consists of a critical examination of

For peer review only - http://bmjopen.bmj.com/site/about/guidelines.xhtml

evidence such as this, producing justified conclusions and transparently discussing any evidence that contradicts these conclusions.

In this review, the strength of the cumulative evidence for each sub question will be described by summarising what is known, for which age group and diagnoses, and with what level of confidence (considering the hierarchy of evidence and quality rating). Diagnosis, age of onset and cognitive abilities of the children included in the research is likely to vary widely and will be an important consideration when summarising what is known. An assessment of the heterogeneity of the evidence and whether there is any potential for evidence synthesis will be undertaken and described. Cultural and economic differences between countries will be an important consideration in relation to the external validity or transferability of findings from individual studies. Any knowledge gaps will be identified and potential directions for future research will be discussed for each sub question.

ETHICS AND DISSEMINATION

Ethical approval is not required to undertake this review. The review will be submitted for peer review in an appropriate journal.

REGISTRATION

The review has been registered on the Open Science Framework database. DOI: https://doi.org/10.17605/OSF.IO/SQXJB. Any protocol amendments will be recorded in the Open Science Framework database.

ACKNOWLEDGMENTS

The authors would like to thank public and patient involvement representatives Roger Leek and Jacqueline Hawkins, and librarians Lynne Harris and Abimbola Alayo for their contributions.

CONTRIBUTORSHIP STATEMENT

Authors and contributors listed in the acknowledgements made the following contributions:
Marilyn Bradbury: Conceptualisation, methodology, writing – original draft, writing - review and editing, visualisation, project administration, funding acquisition.
Dr Ciara O'Brien: Methodology, writing - review and editing, project administration.
Nathan Giles: Writing – review and editing, project administration.
Dr. Sally Fenton: Conceptualisation, methodology, writing - review and editing, visualisation.
Dr. Sue Neilson: Conceptualisation, methodology, writing - review and editing, visualisation.
Prof Joan Duda: Conceptualisation, methodology, writing - review and editing, visualisation.
Public and patient involvement representatives, Roger Leek and Jacqueline Hawkins: Writing – review and editing.

COMPETING INTERESTS STATEMENT

All authors have completed the ICMJE uniform disclosure form at www.icmje.org/coi_disclosure.pdf and declare: Marilyn Bradbury had financial support from the National Institute of Health Research for the submitted work; no financial relationships with any organisations that might have an interest in the submitted work in the previous three years; no other relationships or activities that could appear to have influenced the submitted work.

For peer review only - http://bmjopen.bmj.com/site/about/guidelines.xhtml

For peer review only

FUNDING

This work was supported by the National Institute for Health Research (NIHR), grant number ICA-CDRF-2018-04-ST2-021. The funder has not been involved in the development of this protocol.

Marilyn Bradbury, Clinical Doctoral Research Fellow, ICA-CDRF-2018-04-ST2-021 is funded by Health Education England (HEE) / National Institute for Health Research (NIHR) for this research project. The views expressed in this publication are those of the author(s) and not necessarily those of the NIHR, (Partner Name), NHS or the UK Department of Health and Social Care.

REFERENCES

1. Sedentary Behaviour Research Network. Letter to the Editor: Standardized use of the terms "sedentary" and "sedentary behaviours". *Appl Physiol Nutr Metab* Online [eLetter]. 27 April 2012. https://doi.org/10.1139/h2012-024.
2. Tremblay MS, LeBlanc AG, Kho ME, et al. Systematic review of sedentary behaviour and health indicators in school-aged children and youth. *Int J Behav Nutr Phys Act*. 2011;8(1):98.
3. Ganz F, Hammam N, Pritchard L. Sedentary behavior and children with physical disabilities: a scoping review. *Disabil Rehabil*. 2020:1-13.
4. Oftedal S, Bell KL, Davies PS, et al. Sedentary and Active Time in Toddlers with and without Cerebral Palsy. *Med Sci Sports Exerc*. 2015;47(10):2076-83.
5. Shkedy Rabani A, Harries N, Namoora I, et al. Duration and patterns of habitual physical activity in adolescents and young adults with cerebral palsy. *Dev Med Child Neurol*. 2014;56(7):673-80.
6. Keawutan P, Bell KL, Oftedal S, et al. Longitudinal physical activity and sedentary behaviour in preschool-aged children with cerebral palsy across all functional levels. *Dev Med Child Neurol*. 2017.
7. Maher A, Williams T, Olds T, et al. Physical and sedentary activity in adolescents with cerebral palsy. *Dev Med Child Neurol*. 2007;49(6):450.
8. Luther BL. Food and physical activity choices to prevent overweight in children with spina bifida. *Dissertation Abstracts International: Section B: The Sciences and Engineering*. 2010;71(6-B):3597.
9. Slavin RE. Best evidence synthesis: An intelligent alternative to meta-analysis. *Journal of Clinical Epidemiology*. 1995;48(1):9-18.
10. Grant MJ, Booth A. A typology of reviews: an analysis of 14 review types and associated methodologies. *Health Info Libr J*. 2009;26(2):91-108.
11. Cooke A, Smith D, Booth A. Beyond PICO: the SPIDER tool for qualitative evidence synthesis. *Qual Health Res*. 2012;22(10):1435-43.
12. The Joanna Briggs Institute. The Joanna Briggs Institute reviewers' manual 2015: methodology for JBI scoping reviews: The Joanna Briggs Institute 2015: 10-11.
13. The Endnote Team. EndNote. EndNote X7 ed. Philadelphia, PA: Clarivate; 2013.
14. Boutron I, Page M, Higgins J, et al. Considering bias and conflicts of interest among the included studies. In: Higgins J, Thomas J, Chandler J, et al., eds. Cochrane Handbook for Systematic Reviews of Interventions Version 62 (updated February 2021): Cochrane 2021.
15. National Heart Lung and Blood Institute. Study Quality Assessment Tools: U.S Department of Health& Human Services; 2017 [Available from: https://www.nhlbi.nih.gov/health-topics/study-quality-assessment-tools. Date Accessed 01/04/2021.
16. Critical Appraisal Skills Programme. CASP Checklists: CASP UK; 2020 [Available from: http://www.casp-uk.net/casp-tools-checklists. Date Accessed 01/04/2021.

For peer review only - http://bmjopen.bmj.com/site/about/guidelines.xhtml

17. Higgins JPT, Altman DG, Gøtzsche PC, et al. The Cochrane Collaboration's tool for assessing risk of bias in randomised trials. *British Medical Journal*. 2011;343:d5928.
18. Hong QN, Fàbregues Feijóo S, Bartlett G, et al. The Mixed Methods Appraisal Tool (MMAT) version 2018 for information professionals and researchers. *Education for information*. 2018.

EXCLUSIVE LICENCE

I, the Submitting Author has the right to grant and does grant on behalf of all authors of the Work (as defined in the below author licence), an exclusive licence and/or a non-exclusive licence for contributions from authors who are: i) UK Crown employees; ii) where BMJ has agreed a CC-BY licence shall apply, and/or iii) in accordance with the terms applicable for US Federal Government officers or employees acting as part of their official duties; on a worldwide, perpetual, irrevocable, royalty-free basis to BMJ Publishing Group Ltd ("BMJ") its licensees and where the relevant Journal is co-owned by BMJ to the co-owners of the Journal, to publish the Work in BMJ Open and any other BMJ products and to exploit all rights, as set out in our licence.

The Submitting Author accepts and understands that any supply made under these terms is made by BMJ to the Submitting Author unless you are acting as an employee on behalf of your employer or a postgraduate student of an affiliated institution which is paying any applicable article publishing charge ("APC") for Open Access articles. Where the Submitting Author wishes to make the Work available on an Open Access basis (and intends to pay the relevant APC), the terms of reuse of such Open Access shall be governed by a Creative Commons licence – details of these licences and which Creative Commons licence will apply to this Work are set out in our licence referred to above.

For peer review only

Sedentary Behaviour in Non-Ambulant Children and Young People with Physical Disabilities: A Systematic Search and Review Protocol

Search strategy developed using “Population, Concept, Context” framework.

<table>
<tr><th>Database</th><th>Population</th><th>Concept</th><th>Limits applied</th></tr>
<tr><td>AMED - The Allied and Complementary Medicine Database
Via EBSCOhost</td><td rowspan="5">“physical disabilit*” (KW) OR “physically disabled” (KW) OR “non-ambulant” (KW) OR “non-ambulatory” (KW) OR wheelchair* (KW) OR “limited mobility” (KW) OR “immobil*”(KW) OR “mobility impairment” (KW) OR “neuromusculoskeletal disabilit*” (KW) OR amputee* (KW) OR “cerebral pals*” OR diplegi* (KW) OR quadripleg* (KW) OR tetrapleg* (KW) OR “muscular dystroph*” OR “spina bifida” (KW) OR “spinal dysraphism” (KW) OR “neural tube defect*” (KW) OR parapleg* (KW) paralys* (KW) OR “spinal cord injur*” OR handicap* (KW) OR “hereditary motor sensory neuropath*” (KW) OR “charcot-marie-tooth” (KW) OR "spinal muscular atroph*" (KW)

AND

minors (KW) OR pediatric (KW) OR paediatrics (KW) OR pediatric (KW) OR paediatric (KW) OR child (KW) OR children (KW) OR baby (KW) OR childhood (KW) OR babies (KW) OR infant (KW) OR infants (KW) OR infancy (KW) OR school (KW) OR preschool (KW) OR adolescen* (KW) OR teen* (KW) OR youth (KW) OR "young adult*" (KW) OR "young person*" (KW) OR "young people" (KW) OR toddler* (KW) OR juvenile* (KW)</td><td rowspan="5">sedentary (KW) OR “screen time" (KW) OR screentime (KW) OR "screen-time" (KW) OR "screen use" (KW) OR "screen exposure" (KW) OR (television or TV) NEAR/3 watch* OR "physical inactivity" (KW) OR "physically inactive" (KW) OR sitting (KW)</td><td>Published date: 1996 – 2021
Language: English</td></tr>
<tr><td>Applied Social Sciences Index & Abstracts (ASSIA)
Via ProQuest</td><td>Publication date: 1996-2021
English language</td></tr>
<tr><td>Child development and adolescent studies
Via EBSCOhost</td><td>Publication date: 1996 – 2021</td></tr>
<tr><td>ERIC
Via EBSCOhost</td><td>Date Published: Jan 1996 – 2021
English Language</td></tr>
<tr><td>SPORTDiscus
Via EBSCOhost</td><td>Publication Date: Jan 1996 – 2021
English Language</td></tr>
<tr><td>APA PsycINFO
Via Ovid</td><td>“physical disabilit*” (KW) OR “physically disabled” (KW) OR “non-ambulant” (KW) OR “non-ambulatory” (KW) OR wheelchair* (KW) OR “limited mobility” (KW) OR “immobil*”(KW) OR “mobility impairment” (KW) OR “neuromusculoskeletal disability” (KW) (NB wouldn’t accept “neuromusculoskeletal disabilit*”) OR amputee*</td><td>sedentary (KW) OR “screen time" (KW) OR screentime (KW) OR "screen-time" (KW) OR "screen use" (KW) OR</td><td>Publication Year: 1996 – 2021
English language</td></tr>
</table>

For peer review only - http://bmjopen.bmj.com/site/about/guidelines.xhtml

	(KW) OR “cerebral pals*” OR diplegi* (KW) OR quadripleg* (KW) OR tetrapleg* (KW) OR “muscular dystroph*” OR “spina bifida” (KW) OR “spinal dysraphism” (KW) OR “neural tube defect*” (KW) OR parapleg* (KW) paralys* (KW) OR “spinal cord injur*” OR handicap* (KW) OR “hereditary motor sensory neuropath*” (KW) OR “charcot-marie-tooth” (KW) OR "spinal muscular atroph*" (KW)<br>OR “Physical disabilities (Attitudes toward)” (SH) (exp) OR Mobility aids (SH) (exp) OR Amputation (SH) (exp) OR Cerebral Palsy (SH) (exp) OR Quadriplegia (SH) (exp) OR Muscular Dystrophy (SH) (exp) OR Spina Bifida (SH) (exp) OR Paralysis (SH) (exp) OR Spinal Cord Injuries (SH) (exp)<br>OR Paragplegia (SH) (exp) OR Charcot-Marie-Tooth Disease (SH) (exp)<br><br>AND<br><br>minors OR pediatric OR paediatrics OR pediatrics OR paediatric OR child OR children OR baby OR childhood OR babies OR infant OR infants OR infancy OR school OR preschool OR adolescen* OR teen* OR youth OR "young adult*" OR "young person*" OR "young people" OR toddler* OR juvenile*	"screen exposure" (KW) OR (television or TV) adj3 watch* OR "physical inactivity" (KW) OR "physically inactive" (KW) OR sitting (KW) OR Sedentary behavior (SH) (exp) OR Television viewing (SH) (exp) OR Television (SH) (exp) OR Screen Time (SH) (exp)	
**CINAHL Plus via EBSCOhost**	“physical disabilit*” (KW) OR “physically disabled” (KW) OR “non-ambulant” (KW) OR “non-ambulatory” (KW) OR wheelchair* (KW) OR “limited mobility” (KW) OR “immobil*”(KW) OR “mobility impairment” (KW) OR “neuromusculoskeletal disabilit*” (KW) OR amputee* (KW) OR “cerebral pals*” OR diplegi* (KW) OR quadripleg* (KW) OR tetrapleg* (KW) OR “muscular dystroph*” OR “spina bifida” (KW) OR “spinal dysraphism” (KW) OR “neural tube defect*” (KW) OR parapleg* (KW) paralys* (KW) OR “spinal cord injur*” OR handicapped (KW) OR “hereditary motor sensory neuropath*” (KW) OR “charcot-marie-tooth” (KW) OR "spinal muscular atroph*" (KW) OR Immobility (SH) OR Wheelchairs (SH) (exp) OR Amputees (SH) (exp) OR Cerebral palsy (SH) OR Muscular Dystrophy (SH) (exp) OR Spina Bifida (SH) OR Neural Tube Defects (SH) (exp) OR Paraplegia (SH) (exp) OR Spastic Paraplegia, Hereditary (SH) OR Spinal Cord Injuries (SH) (exp) OR Quadriplegia (SH) (exp) OR Neuropathies, Hereditary motor and sensory (exp) OR Charcot-Marie-Tooth Disease (SH) OR Muscular Atrophy, Spinal (SH) (exp)<br><br>AND<br><br>minors (KW) OR pediatric (KW) OR paediatrics (KW) OR pediatric (KW) OR paediatric (KW) OR child (KW) OR	sedentary (KW) OR “screen time” (KW) OR screentime (KW) OR "screen-time" (KW) OR "screen use" (KW) OR "screen exposure" (KW) OR "(television or TV) near/3 watch* OR "physical inactivity" (KW) OR "physically inactive" (KW) OR Life Style, Sedentary (SH) (exp) OR Television (SH) OR Screen Time (SH) OR Sitting (SH)	Publication date: 1996 – 2021<br>Language: English

For peer review only - http://bmjopen.bmj.com/site/about/guidelines.xhtml

	children (KW) OR baby (KW) OR childhood (KW) OR babies (KW) OR infant (KW) OR infants (KW) OR infancy (KW) OR school (KW) OR preschool (KW) OR adolescen* (KW) OR teen* (KW) OR youth (KW) OR "young adult*" (KW) OR "young person*" (KW) OR "young people" (KW) OR toddler* (KW) OR juvenile* (KW) OR Infant, Newborn (SH) (exp) OR Minors (Legal) (SH) (exp) OR Child (SH) (exp) OR Adolescence (SH) (exp)		
**Cochrane Library**<br><br>**Via Cochrane library website**	Searched title and abstract only for:<br>"physical disability" OR "physical disabilities" OR "physically disabled" OR "non-ambulant" OR "non-ambulatory" OR wheelchair* OR "limited mobility" OR immobilit* OR "mobility impairment" OR "neuromusculoskeletal disabilit*" OR "neuromusculoskeletal disability" OR amputee* OR "cerebral palsy" OR diplegi* OR quadripleg* OR tetrapleg* OR "muscular dystrophy" OR "spina bifida" OR "spinal dysraphism" OR "neural tube defect" OR "neural tube defects" OR parapleg* OR paralys* OR "spinal cord injury" OR "spinal cord injuries" OR handicap* OR "hereditary motor sensory neuropathy" OR "hereditary motor sensory neuropathies" OR "charcot-marie-tooth" OR "spinal muscular atrophy" OR "spinal muscular atrophies" OR<br>Mobility Limitation (MeSH) (exp) OR Amputees (MeSH) (exp) OR<br>OR Wheelchairs (MeSH) (exp) OR<br>Cerebral Palsy (MeSH) (exp)<br>OR Muscular Dystrophies (MeSH) (exp)<br>OR Spinal Dysraphism (MeSH) (exp)<br>OR Neural Tube Defects (MeSH) (exp) OR Spinal Cord Injuries (MeSH) OR Paraplegia (MeSH) (exp) OR Quadriplegia (MeSH) (exp) OR Paralysis (MeSH) (exp) OR Spastic Paraplegia, Hereditary (MeSH) (exp) OR Hereditary Sensory and Motor Neuropathy (MeSH) (exp) OR Charvot-Marie-Tooth Disease (MeSH) (exp) OR Muscular Atrohy, Spinal (MeSH) (exp)<br><br>AND<br><br>minors OR pediatric OR paediatrics OR pediatrics OR paediatric OR child OR children OR baby OR childhood OR babies OR infant OR infants OR infancy OR school OR preschool OR adolescen* OR teen* OR youth OR "young adult" OR "young adults" OR "young person" OR "young people" OR toddler* OR juvenile*) OR Young Adult (MeSH) OR Adolescent (MeSH) OR (MeSH) OR Infant (MeSH) OR Child (MeSH)	Searched title and abstract only for:<br>sedentary (KW) OR "screen time" (KW) OR screentime (KW) OR "screen-time" (KW) OR "screen use" (KW) OR "screen exposure" (KW) OR (television or TV) near/3 watch* OR "physical inactivity" (KW) OR "physically inactive" (KW) OR Sedentary Behavior (MeSH) (exp) OR Television (MeSH) (exp) OR Sitting position (MeSH) (exp) OR Screen Time (MeSH) (exp)	Cochrane Library publication date: Jan 1996 and 2021<br><br>Content type: Cochrane reviews Cochrane trials
**EMBASE: Excerpta Medica via Ovid**	"physical disabilit*" (KW) OR "physically disabled" (KW) OR "non-ambulant" (KW) OR "non-ambulatory" (KW) OR wheelchair* (KW) OR "limited mobility" (KW) OR	sedentary (KW) OR "screen time" (KW) OR screentime (KW)	Publication year: 1996 – 2021

For peer review only - http://bmjopen.bmj.com/site/about/guidelines.xhtml

	“immobil*”(KW) OR “mobility impairment” (KW) OR “neuromusculoskeletal disability” (KW) (NB wouldn’t accept “neuromusculoskeletal disabilit*” OR amputee* (KW) OR “cerebral pals*” OR diplegi* (KW) OR quadripleg* (KW) OR tetrapleg* (KW) OR “muscular dystroph*” OR “spina bifida” (KW) OR “spinal dysraphism” (KW) OR “neural tube defect*” (KW) OR parapleg* (KW) paralys* (KW) OR “spinal cord injur*” OR handicap* (KW) OR “hereditary motor sensory neuropath*” (KW) OR “charcot-marie-tooth” (KW) OR "spinal muscular atroph*" (KW) OR Physical disability (SH) (exp) OR Wheelchair (SH) (exp) OR Limited Mobility (SH) (exp) OR Cerebral palsy (SH) (exp) (KW) OR Amputee (SH) (exp) OR Muscular dystrophy (SH) (exp) OR Duchenne Muscular Dystrophy (SH) (exp) OR Spina Bifida (SH) exp) OR Spinal Dysraphism (SH) (exp) OR Neural Tube Defect (SH) (exp) OR Paraplegia (SH) (exp) OR Paralysis (SH) (exp) OR Spastic paraplegia (SH) (exp) OR Quadriplegia (SH) (exp) OR Spinal cord injury (SH) (exp) OR Hereditary Motor Sensory Neuropathy (SH) (exp) OR Muscular Atrophy, Spinal (SH) (exp)<br><br>AND<br><br>minors (KW) OR pediatric (KW) OR paediatrics (KW) OR pediatric (KW) OR paediatric (KW) OR child (KW) OR children (KW) OR baby (KW) OR childhood (KW) OR babies (KW) OR infant (KW) OR infants (KW) OR infancy (KW) OR school (KW) OR preschool (KW) OR adolescen* (KW) OR teen* (KW) OR youth (KW) OR "young adult*" (KW) OR "young person*" (KW) OR "young people" (KW) OR toddler* (KW) OR juvenile* (KW) OR Young Adult (SH) (exp) OR Adolescent (SH) (exp) OR (SH) (exp) OR Infant (SH) (exp) OR Child (SH) (exp)	OR "screen-time" (KW) OR "screen use" (KW) OR "screen exposure" (KW) OR (television or TV) adj3 watch* OR "physical inactivity" (KW) OR "physically inactive" (KW) OR Sedentary lifestyle (SH) (exp) OR Sedentary time (SH) (exp) OR Screen time (SH) (exp) OR Television (SH) (exp) OR Television viewing (SH) (exp) OR Sitting (SH) (exp) OR Physical Inactivity (SH) (exp)	English language
**MEDLINE (R) Via Ovid**	“physical disabilit*” (KW) OR “physically disabled” (KW) OR “non-ambulant” (KW) OR “non-ambulatory” (KW) OR wheelchair* (KW) OR “limited mobility” (KW) OR “immobil*”(KW) OR “mobility impairment” (KW) OR “neuromusculoskeletal disabilit*” (KW) OR amputee* (KW) OR “cerebral pals*” OR diplegi* (KW) OR quadripleg* (KW) OR tetrapleg* (KW) OR “muscular dystroph*” OR “spina bifida” (KW) OR “spinal dysraphism” (KW) OR “neural tube defect*” (KW) OR parapleg* (KW) paralys* (KW) OR “spinal cord injur*” OR handicap* (KW) OR “hereditary motor sensory neuropath*” (KW) OR “charcot-marie-tooth” (KW) OR "spinal muscular atroph*" (KW) OR Wheelchairs (SH) (exp) OR Mobility Limitation (SH) (exp) OR Cerebral palsy (SH) (exp) (KW) OR Amputees (SH) (exp) OR Muscular dystrophies (SH) (exp) OR Spinal Dysraphism (SH) (exp) (KW) OR Neural Tube Defects (SH) (exp) OR	sedentary (KW) OR “screen time” (KW) OR screentime (KW) OR "screen-time" (KW) OR "screen use" (KW) OR "screen exposure" (KW) OR (television or TV) adj3 watch* OR "physical inactivity" (KW) OR "physically inactive" (KW) Sedentary Behavior (SH) (exp) OR Television (SH) (exp) OR Screen Time (SH) (exp) OR	Publication Year: 1996 – 2021 English language

For peer review only - http://bmjopen.bmj.com/site/about/guidelines.xhtml

	Paraplegia (SH) (exp) OR Paralysis (SH) (exp) OR Spastic paraplegia, hereditary (SH) (exp) OR Quadriplegia (SH) (exp) OR Spinal cord injuries (SH) (exp) OR Hereditary Sensory and Motor Neuropathy (SH) (exp) OR Charcot-Marie-Tooth Disease (SH) (exp) OR Muscular Atrophy, Spinal (SH) (exp)<br><br>AND<br><br>minors (KW) OR pediatric (KW) OR paediatrics (KW) OR pediatric (KW) OR paediatric (KW) OR child (KW) OR children (KW) OR baby (KW) OR childhood (KW) OR babies (KW) OR infant (KW) OR infants (KW) OR infancy (KW) OR school (KW) OR preschool (KW) OR adolescen* (KW) OR teen* (KW) OR youth (KW) OR "young adult*" (KW) OR "young person*" (KW) OR "young people" (KW) OR toddler* (KW) OR juvenile* (KW) OR Minors (SH) (exp) OR Child (SH) (exp) OR Child, Preschool (SH) (exp) OR Infant, Newborn (SH) (exp) OR Infant (SH) (exp) OR Adolescent (SH) (exp) OR Young Adult (SH) (exp)	Sitting Position (SH) (exp) OR	
**Nursing and Allied Health Database Via ProQuest**	“physical disabilit*” (KW) OR “physically disabled” (KW) OR “non-ambulant” (KW) OR “non-ambulatory” (KW) OR wheelchair* (KW) OR “limited mobility” (KW) OR “immobil*”(KW) OR “mobility impairment” (KW) OR “neuromusculoskeletal disabilit*” (KW) OR amputee* (KW) OR “cerebral pals*” OR diplegi* (KW) OR quadripleg* (KW) OR tetrapleg* (KW) OR “muscular dystroph*” OR “spina bifida” (KW) OR “spinal dysraphism” (KW) OR “neural tube defect*” (KW) OR parapleg* (KW) paralys* (KW) OR “spinal cord injur*” OR handicap* (KW) OR “hereditary motor sensory neuropath*” (KW) OR “charcot-marie-tooth” (KW) OR "spinal muscular atroph*" (KW) OR Neural Tube Defects (MeSH) OR Spastic Paraplegia, Hereditary (MeSH) OR Spina Bifida Occulta (MeSH) OR Amputees (MeSH) OR Quadriplegia (MeSH) OR Spina Bifida Cystica (MeSH) OR Charcot-Marie-Tooth Disease (MeSH) OR Mobility Limitation (MeSH) OR Paraplegia (MeSH) OR Muscular Dystrophies (MeSH) OR Spinal Cord Injuries (MeSH) OR Wheelchairs (MeSH) OR Muscular Atrophy, Spinal (MeSH) OR Spinal Dysraphism (MeSH) OR Hereditary Sensory & Motor Neuropathy (MeSH) OR Muscular Dystrophy, Duchenne (MeSH) OR Paralysis (MeSH) OR Cerebral Palsy (MeSH)<br><br>AND<br><br>minors (KW) OR pediatric (KW) OR paediatrics (KW) OR pediatric (KW) OR paediatric (KW) OR child (KW) OR children (KW) OR baby (KW) OR childhood (KW) OR	sedentary (KW) OR “screen time” (KW) OR screentime (KW) OR "screen-time" (KW) OR "screen use" (KW) OR "screen exposure" (KW) OR (television or TV) NEAR/3 watch* (KW) OR "physical inactivity" (KW) OR "physically inactive" (KW) OR sitting (KW) OR Sedentary lifestyle (MeSH) OR Television (MeSH)	Publication date: 01/01/1996 – 2021 English language only

For peer review only - http://bmjopen.bmj.com/site/about/guidelines.xhtml

	babies (KW) OR infant (KW) OR infants (KW) OR infancy (KW) OR school (KW) OR preschool (KW) OR adolescen* (KW) OR teen* (KW) OR youth (KW) OR "young adult*" (KW) OR "young person*" (KW) OR "young people" (KW) OR toddler* (KW) OR juvenile* (KW) OR Young Adult (MeSH) OR Adolescent (MeSH) OR (MeSH) OR Infant (MeSH) OR Child (MeSH)		
**Scopus**	Title and abstract search only (not key words).<br>“physical disability*” (KW) OR “physically disabled” (KW) OR “non-ambulant” (KW) OR “non-ambulatory” (KW) OR wheelchair* (KW) OR “limited mobility” (KW) OR “immobil*”(KW) OR “mobility impairment” (KW) OR “neuromusculoskeletal disabilit*” (KW) OR amputee* (KW) OR “cerebral pals*” OR diplegi* (KW) OR quadripleg* (KW) OR tetrapleg* (KW) OR “muscular dystroph*” OR “spina bifida” (KW) OR “spinal dysraphism” (KW) OR “neural tube defect*” (KW) OR parapleg* (KW) paralys* (KW) OR “spinal cord injur*” OR handicap* (KW) OR “hereditary motor sensory neuropath*” (KW) OR “charcot-marie-tooth” (KW) OR "spinal muscular atroph*" (KW)<br><br>AND<br><br>minors (KW) OR pediatric (KW) OR paediatrics (KW) OR pediatric (KW) OR paediatric (KW) OR child (KW) OR children (KW) OR baby (KW) OR childhood (KW) OR babies (KW) OR infant (KW) OR infants (KW) OR infancy (KW) OR school (KW) OR preschool (KW) OR adolescen* (KW) OR teen* (KW) OR youth (KW) OR "young adult*" (KW) OR "young person*" (KW) OR "young people" (KW) OR toddler* (KW) OR juvenile* (KW)	Title and abstract search only (not key words).<br>sedentary (KW) OR “screen time" (KW) OR screentime (KW) OR "screen-time" (KW) OR "screen use" (KW) OR "screen exposure" (KW) OR (television or TV) NEAR/3 watch* OR "physical inactivity" (KW) OR "physically inactive" (KW) OR sitting	Publication year: 1996-2021 English language
**Web of Science Core Collection**	Searched Title and Abstract for:<br>“physical disabilit*” OR “physically disabled” OR “non-ambulant” OR “non-ambulatory” OR wheelchair* OR “limited mobility” OR immobil* OR “mobility impairment” OR “neuromusculoskeletal disabilit*” OR amputee* OR “cerebral pals*” OR diplegi* OR quadripleg* OR tetrapleg* OR “muscular dystroph*” OR “spina bifida” OR “spinal dysraphism” OR “neural tube defect*” OR parapleg* OR paralys* OR “spinal cord injur*” OR handicap* OR “hereditary motor sensory neuropath*” OR “charcot-marie-tooth” OR “spinal muscular atroph*”<br><br>AND<br><br>minors OR pediatric OR paediatrics OR pediatrics OR paediatric OR child OR children OR baby OR childhood OR babies OR infant OR infants OR infancy OR school OR	Searched Title and Abstract for:<br>sedentary OR “screen time" OR screentime OR "screen-time" OR "screen use" OR "screen exposure" OR (television or TV) NEAR/3 watch* OR "physical inactivity" OR "physically inactive" OR sitting	Custom year range 1996-2021 English language

For peer review only - http://bmjopen.bmj.com/site/about/guidelines.xhtml

	preschool OR adolescen* OR teen* OR youth OR "young adult*" OR "young person*" OR "young people" OR toddler* OR juvenile*		

For peer review only

PRISMA-P (Preferred Reporting Items for Systematic review and Meta-Analysis Protocols) 2015 checklist: recommended items to address in a systematic review protocol*

Section and topic	Item No	Checklist item	Page Number
**ADMINISTRATIVE INFORMATION**			
Title:			
Identification	1a	Identify the report as a protocol of a systematic review	1
Update	1b	If the protocol is for an update of a previous systematic review, identify as such	
Registration	2	If registered, provide the name of the registry (such as PROSPERO) and registration number	12
Authors:			
Contact	3a	Provide name, institutional affiliation, e-mail address of all protocol authors; provide physical mailing address of corresponding author	1, 12
Contributions	3b	Describe contributions of protocol authors and identify the guarantor of the review	
Amendments	4	If the protocol represents an amendment of a previously completed or published protocol, identify as such and list changes; otherwise, state plan for documenting important protocol amendments	NA
Support:			
Sources	5a	Indicate sources of financial or other support for the review	
Sponsor	5b	Provide name for the review funder and/or sponsor	
Role of sponsor or funder	5c	Describe roles of funder(s), sponsor(s), and/or institution(s), if any, in developing the protocol	12-13
**INTRODUCTION**			
Rationale	6	Describe the rationale for the review in the context of what is already known	3-5
Objectives	7	Provide an explicit statement of the question(s) the review will address with reference to participants, interventions, comparators, and outcomes (PICO)	4-7 using PCC rather than PICO
**METHODS**			
Eligibility criteria	8	Specify the study characteristics (such as PICO, study design, setting, time frame) and report characteristics (such as years considered, language, publication status) to be used as criteria for eligibility for the review	5-7
Information sources	9	Describe all intended information sources (such as electronic databases, contact with study authors, trial registers or other grey literature sources) with planned dates of coverage	4-9

For peer review only

Search strategy	10	Present draft of search strategy to be used for at least one electronic database, including planned limits, such that it could be repeated	7-8 and supplementary file 1
Study records:			
Data management	11a	Describe the mechanism(s) that will be used to manage records and data throughout the review	8-9
Selection process	11b	State the process that will be used for selecting studies (such as two independent reviewers) through each phase of the review (that is, screening, eligibility and inclusion in meta-analysis)	9
Data collection process	11c	Describe planned method of extracting data from reports (such as piloting forms, done independently, in duplicate), any processes for obtaining and confirming data from investigators	9-11
Data items	12	List and define all variables for which data will be sought (such as PICO items, funding sources), any pre-planned data assumptions and simplifications	10-11
Outcomes and prioritization	13	List and define all outcomes for which data will be sought, including prioritization of main and additional outcomes, with rationale	9-11
Risk of bias in individual studies	14	Describe anticipated methods for assessing risk of bias of individual studies, including whether this will be done at the outcome or study level, or both; state how this information will be used in data synthesis	9-12
Data synthesis	15a	Describe criteria under which study data will be quantitatively synthesised	11-12
	15b	If data are appropriate for quantitative synthesis, describe planned summary measures, methods of handling data and methods of combining data from studies, including any planned exploration of consistency (such as I^2, Kendall's τ)	Potential to be assessed as part of data analysis
	15c	Describe any proposed additional analyses (such as sensitivity or subgroup analyses, meta-regression)	NA
	15d	If quantitative synthesis is not appropriate, describe the type of summary planned	Appropriateness to be assessed as part of analysis
Meta-bias(es)	16	Specify any planned assessment of meta-bias(es) (such as publication bias across studies, selective reporting within studies)	NA
Confidence in cumulative evidence	17	Describe how the strength of the body of evidence will be assessed (such as GRADE)	9-12

*** It is strongly recommended that this checklist be read in conjunction with the PRISMA-P Explanation and Elaboration (cite when available) for important clarification on the items. Amendments to a review protocol should be tracked and dated. The copyright for PRISMA-P (including checklist) is held by the PRISMA-P Group and is distributed under a Creative Commons Attribution Licence 4.0.**

For peer review only

From: Shamseer L, Moher D, Clarke M, Ghersi D, Liberati A, Petticrew M, Shekelle P, Stewart L, PRISMA-P Group. Preferred reporting items for systematic review and meta-analysis protocols (PRISMA-P) 2015: elaboration and explanation. BMJ. 2015 Jan 2;349(jan02 1):g7647.

For peer review only
